# Bilayer-folded lamellar mesophase induced by random polymer sequence

**DOI:** 10.1038/s41467-022-30122-z

**Published:** 2022-05-04

**Authors:** Minjoong Shin, Hayeon Kim, Geonhyeong Park, Jongmin Park, Hyungju Ahn, Dong Ki Yoon, Eunji Lee, Myungeun Seo

**Affiliations:** 1 Department of Chemistry, Korea Advanced Institute of Science and Technology (KAIST), Daejeon, 34141 Republic of Korea; 2 School of Materials Science and Engineering, Gwangju Institute of Science and Technology (GIST), Gwangju, 61005 Republic of Korea; 3 Pohang Accelerator Laboratory (PAL), Pohang, 37673 Republic of Korea; 4 Graduate School of Nanoscience and Technology, KAIST, Daejeon, 34141 Republic of Korea; 5 KAIST Institute for the Nanocentury, KAIST, Daejeon, 34141 Republic of Korea

**Keywords:** Polymers, Liquid crystals, Polymers

## Abstract

Randomness is perceived in two different extremes, in macroscopic homogeneity and local heterogeneity, but apparently far away from order. Here we show that a periodic order spontaneously arises from a binary random copolymer when self-assembly occurs in an ensemble containing > 10^15^ possible chain sequences. A Bernoullian distribution of hydrophilic and hydrophobic side chains grafted onto a linear backbone was constructed by random copolymerization. When the polymer chains associate in water, a sequence matching problem occurs because of the drastic heterogeneity in sequence: this is believed to generate local curvature mismatches which deviate from the ensemble-averaged interfacial curvature. Periodic folding of the self-assembled bilayer stabilizes the curvature instability as recurring hinges. Reminiscent of chain-folded lamellae found in polymer crystallization, this new liquid crystalline mesophase, characterized as bilayer-folded lamellae, manifests itself as an anisotropically alignable birefringent hydrogel with structural hierarchy across multiple length scales.

## Abstract (web summary)

Randomness is perceived in two different extremes, in macroscopic homogeneity and local heterogeneity, but apparently far away from order. Here, the authors show that a periodic order can spontaneously arise from an ensemble of binary random copolymer sequences to induce recurrent folding of a self-assembled bilayer structure in water.

## Introduction

Flipping a coin with a constant bias results in a discrete probability distribution called the Bernoulli distribution^1,2^. When the coin toss is unbiased, a random binary sequence with equal probability is obtained. In polymerization, a Bernoulli distribution occurs when a propagating center for polymerization has no preference towards the addition of two different monomers^3^. As a zeroth-order Markov process, the probability for the addition only depends on the monomer feed composition. The resulting additions are consecutively encoded in the polymer chain sequence and fixed in a covalent manner.

**Fig. 1:**
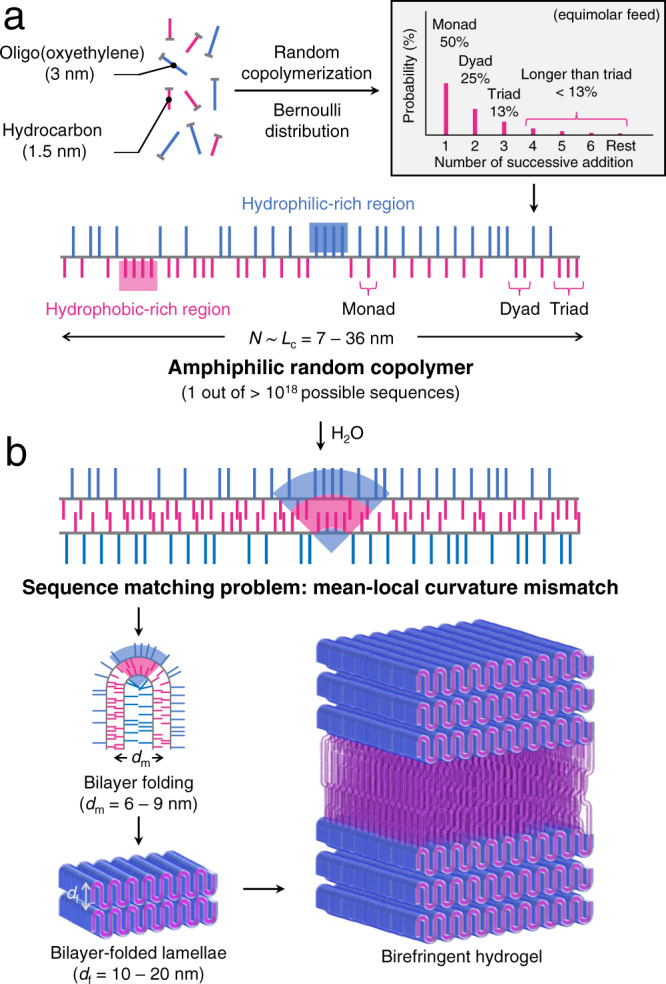
Bilayer folding of an amphiphilic random copolymer in an aqueous solution. **a** The random copolymerization of monomers containing hydrophilic oligo(oxyethylene) and hydrophobic hydrocarbon groups yields an amphiphilic random copolymer. Theoretical sequence-length distribution of the hydrophobic groups along the polymer backbone is given for an equimolar feed^3^, and a hypothetical chain sequence out of many possible arrangements (exponential to *N*) is depicted for *N* = 60. *L*_c_ represents the contour length of the backbone, which is proportional to *N*. **b** The amphiphilic random copolymer self-assembles in water into a bilayer with an interlayer spacing of *d*_m_. The random sequence forces to generate numerous mismatches in local hydrophilic-phobic balance. A hinge will be generated by folding where the local curvature noticeably mismatches the mean packing parameter, and this results in the bilayer-folded lamella. They stack further with a spacing of *d*_f_, connecting the unfolded domains, and turning the whole solution into an anisotropic hydrogel. For simplicity, the bilayers in the folded domains are drawn to be defect-free following the adjacent reentry model in chain-folded crystals.

Amphiphilic random copolymers are produced by the copolymerization of two monomers, which possess hydrophilic and hydrophobic side chains (Fig. 1a). The chain microstructure is quite complex, containing substantial hydrophilic- and phobic-rich local regions generated by the successive addition of a single monomer^3^. This segmental heterogeneity, comparable to intrinsically disordered proteins, has recently been exploited using amphiphilic random copolymers to mimic protein functions such as transmembrane transport and protein stabilization^4,5^. The sequence also drastically varies chain by chain, as the number of possible arrangements for the side chains increases exponentially with the degree of polymerization (*N*). Nearly every macromolecule features a uniquely different sequence at high *N*.

When they self-assemble in water, driven by the association of hydrophobic side chains, their collective behavior seems to follow a mean-field description, smearing heterogeneity in the sequence^6,7^. The average composition of the side chains determines the mean interfacial curvature as the ensemble average^8^. Since the self-assembling length scale is related to the size of the side chains, small pendants facilitate access to the sub-10 nm regime, just as in molecular self-assembly^9^. This is in contrast to amphiphilic block copolymers consisting of hydrophilic and hydrophobic segments, where the characteristic length follows the *N* of the backbone^10^.

Here, we report that the local heterogeneity in the random sequence is manifested at the mesoscopic level by the spontaneous folding of the self-assembled structure. X-ray studies indicate that packing of the alkyl side chains (3–4 angstroms in distance) develops a micellar bilayer, which forms a multilamellar structure separated by a water layer, with a spacing of 6–7 nm between the bilayers. Remarkably, a strong lamellar order with spacings of 10–20 nm develops perpendicular to the bilayer packing. This higher order only appears above a critical *N* and the spacing falls below the extended length of the polymer backbone (contour length *L*_c_), indicating that the bilayer (and the backbone) should periodically fold. While bilayer structures are ubiquitous, from cell membranes to soap bubbles, this has not been known since bending requires additives and energy against the thermodynamically preferred single curvature^11–13^.

We hypothesize that the folding originates with the sequence matching problem in the self-assembly process, because of the drastic heterogeneity among the polymer chains (Fig. 1b). At high *N*, it is nearly impossible to pair perfectly complementary chains in the bilayer configuration without local curvature mismatch. Hinge formation can mitigate the high local curvature deviating from the mean planar geometry. While living organisms also utilize compositional asymmetry to dynamically bend lipid bilayer membranes^13^, the covalently constructed random sequence leads to recurrent folding as a thermodynamically stable state at high concentration. The folded layer further stacks vertically analogous to chain-folded lamellae in semicrystalline polymers^14^. Like the crystalline morphology, the whole phase is thought to consist of the folded domains connected with unfolded regions as “amorphous” domains. The whole solution is characterized as a birefringent, liquid crystalline-like, viscoelastic gel: this new lamellar mesophase, labeled *L*_f_, is distinct from a fluidic multilamellar phase (*L*_α_) observed at high concentrations and the lamellar hydrogel phase (*L*_α,g_) found in a high-water regime^15^.

## Results

### Bilayer-folded lamellar phase

**Fig. 2:**
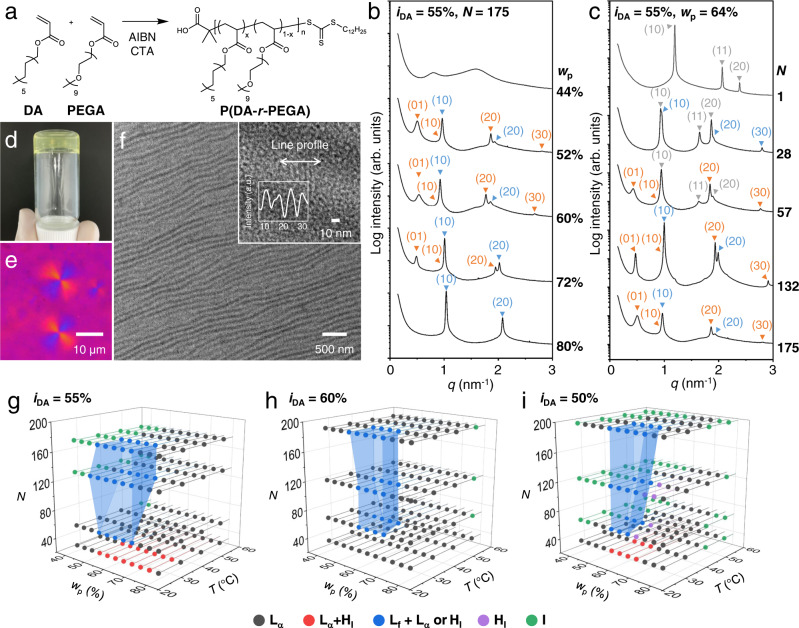
Phase behavior of the amphiphilic random copolymer in water. **a** Synthetic route to P(DA-*r*-PEGA) by RAFT copolymerization. **b** SAXS data of P(DA_96_-*r*-PEGA_75_) as a function of polymer weight fraction (*w*_p_). The data obtained at room temperature are plotted. Peak positions marked with orange and blue triangles are assigned to the structure factors from the bilayer-folded lamellae and the unfolded lamellae. **c** SAXS data of P(DA-*r*-PEGA)s with *i*_DA_ = 55% and different *N*. Concentration is fixed at 64 wt%. The SAXS pattern of C12E9 is also shown as a reference at *N* = 1. Peak positions marked with gray triangles are assigned to the structure factors from the hexagonal geometry. **d** Photo of 64 wt% aqueous solution of P(DA_73_-*r*-PEGA_59_) at room temperature showing the gel state. Yellow color comes from the trithiocarbonate moiety included at the chain end. **e** Polarized optical micrograph of spherulites obtained upon slow cooling (0.5 °C/min) of the 64 wt% solution of P(DA_73_-*r*-PEGA_59_) from the isotropic state at 55 °C. **f** Cross-sectional TEM image of the vitrified solution of P(DA_73_-*r*-PEGA_59_) at 60 wt% concentration. The solution contained 5 wt% of sodium silicate and 0.2 M HCl. The microtomed film was further stained with RuO_4_. **g**–**i** Phase diagrams for the polymers with *i*_DA_ = 55% (**g**), 60% (**h**), and 50% (**i**) as a function of *w*_p_, *N*, and temperature (*T*). The following phases were identified by SAXS analysis: *L*_α_ (fluidic multilamellar, gray); *H*_I_ (hexagonal, violet); *L*_α_ + *H*_I_ (red); *L*_f_ (bilayer-folded lamellar) + *L*_α or_ *H*_I_ (blue); *I* (isotropic, green). Blue shade indicates the phase boundary for the *L*_f_ + *L*_α or_ *H*_I_ phase.

The amphiphilic random copolymers were synthesized by radical copolymerization of dodecyl acrylate (DA) containing a hydrocarbon side chain and oligo(oxyethylene) acrylate with 9 repeating units (PEGA) (Fig. 2a). Reversible addition-fragmentation chain transfer (RAFT) polymerization^16^ was employed to control *N* with narrow distribution. The molecular dimensions of the side chains in the resulting P(DA-*r*-PEGA) are very comparable to C12E9 nonionic surfactant^17^. All polymerizations gave >86% conversion (see Supplementary Figs. 1–5 and Supplementary Table 1 for synthetic details). DA and PEGA were randomly copolymerized throughout the whole polymerization, so the polymer composition could be adjusted by the feed ratio (Supplementary Fig. 6). Well-controlled polymers with dispersities (*Ð*) in the range of 1.16–1.41 were obtained with varying *N* (35–198) and three DA molar fractions (*i*_DA_ = 50, 55, and 60%). Increasing *i*_DA_ further led to poor solubility in water. P(DA-*r*-PEGA)s with *i*_DA_ of 20–50% have been reported to fold into micelles in dilute condition^8^. Liquid crystalline mesophases were not observed from polymers with *i*_DA_ = 40%. We also synthesized a polymethacrylate version of the amphiphilic random copolymer and also methacrylate/acrylate copolymers^18^ to investigate the effect of the backbone stiffness and randomness in sequence (Supplementary Figs. 1, 7 and 8 and Supplementary Table 1).

In the one-dimensional (1D) small-angle X-ray scattering (SAXS) patterns of the polymer aqueous solutions, the appearance of a new, distinct scattering peak in the lower scattering vector *q* labeled (01) in orange in Fig. 2(b, c), marks the presence of the bilayer-folded *L*_f_ phase. Representative SAXS data of the polymer solutions (*N* = 175, *i*_DA_ = 55%) obtained at room temperature are shown in Fig. 2(b) for five concentrations (see Supplementary Figs. 9–13 for the whole SAXS data in the full concentration range). At 44 wt%, the solution is in the isotropic phase with disordered micellar packing. The SAXS pattern at 80 wt% showing (*h*0) diffraction peaks can be clearly assigned to the multilamellar *L*_α_ phase (Supplementary Fig. 14). From the scattering peak position ((10) in blue), the spacing between the micellar bilayers (*d*_m_ = 2π/*q*_(10)_) is estimated to be 6.0 nm. This length scale is comparable to the side chain-driven lamellar packing of amphiphilic random copolymers bearing crystalline octadecyl pendants in the solid state^9^. With increasing polymer concentration (i.e., decreasing water content), the oligo(oxyethylene) chains shrink and the alkyl chains adopt a tighter packing structure. This results in the decrease in *d*_m_ as reflected by the peak shift to a higher *q*. Despite the high polymer content, the solution is fluidic at 80 wt% concentration (Supplementary Fig. 15). Differential scanning calorimetry measurements indicate that the alkyl chains are in the amorphous state at room temperature (Supplementary Fig. 16). Wide-angle X-ray diffraction suggests the alkyl chains are intercalated to some extent and perpendicular to the micellar lamellae without noticeable chain tilt (Supplementary Fig. 17). With increasing concentration, population of hexagonal arrangements gradually increases with the suppression of orthorhombic packing^19^. The polymers were disordered in the absence of water.

In contrast, solutions in the *L*_f_ phase from 52 to 72 wt% form gels and do not flow (Fig. 2d): they are a viscoelastic solid, as storage modulus (*G’*) is higher than loss modulus (*G*”) in the full frequency range (Supplementary Fig. S18). *G’* is also about one order of magnitude higher than the *L*_α_ phase. Three scattering functions with higher harmonics are identified in this concentration regime. The scattering peak at *q* ~ 0.5 nm^−1^ corresponds to stacking of the bilayer-folded lamellae with spacing of *d*_f_ = 11.4–13.4 nm. The *d*_f_/*d*_m_ ratio is not an integer and in the range of 1.59 to 2.09. The contour length at *N* = 175 is estimated to exceed 40 nm, ruling out the extended chain lamellar morphology.

Two different micellar lamellae are present in the *L*_f_ phase as evidenced by the splitting of the (20) peak. Upon heating, only one micellar lamellar structure persists above 45 °C and the bilayer-folded lamellae vanish (Supplementary Fig. S19). The solution becomes isotropic above 55 °C. Cooling restores the original scattering pattern; the *L*_f_ phase is thermodynamically stable. Formation of spherulites upon slow cooling infers radial growth of the bilayer-folded lamellar domains (Fig. 2e and Supplementary Fig. 20). Only line defects are observed from samples outside of the *L*_f_ phase. Transmission electron microscopy (TEM) imaging of the vitrified gel suggests that the folded domains aligned under shear grows horizontally over a large area (Fig. 2f and Supplementary Fig. 21 for a low-magnification image). The layer structure with an average thickness of 30 nm is clearly visualized, presumably originating from contrast between the folded and unfolded regions. Imaging the micellar packing was challenging at the cryo-TEM resolution limit. While the length scale discernable from the inset coincides with the micellar spacing (~6 nm), the micellar order may not be fully preserved in the imaging condition.

The *L*_f_ phase was also found in the polymer solutions with *N* of 57 and 132 (Fig. 2c). A shorter polymer (*N* = 28) and C12E9 (*N* = 1) did not generate the *L*_f_ phase, supporting the conclusion that folding is a backbone length-dependent process. Figure 2g summarizes the phase behavior of the P(DA-*r*-PEGA) solutions as a function of *N*, temperature, and the weight concentration (*w*_p_). P(DA-*r*-PEGA)s with different hydrophobic contents of *i*_DA_ = 60 and 50% also exhibit the *L*_f_ phase, characterized by birefringent gel formation, the emergence of the SAXS peak beyond the micellar length scale, and spherulite formation upon slow cooling (see Fig. 2(h, i) for their phase diagrams, and also Supplementary Figs. 22–S37 for the full characterization data).

**Fig. 3:**
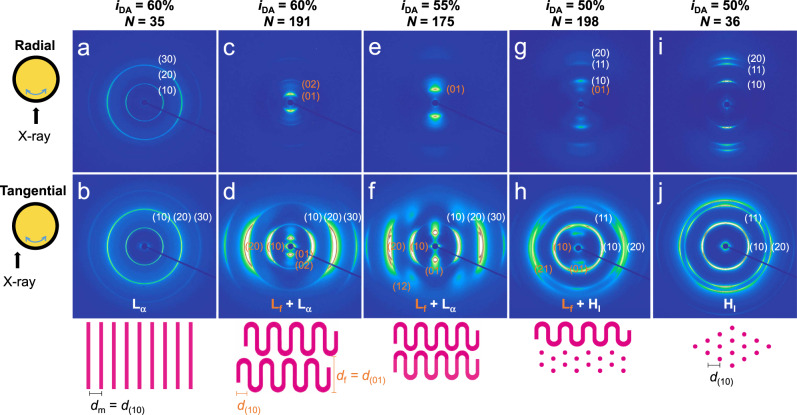
2D SAXS patterns of shear-aligned polymer solutions at 64 wt% concentration. Scatterings obtained along the radial (**a**, **c**, **e**, **g**, **i**) and tangential (**b**, **d**, **f**, **h**, **j**) directions are shown. *i*_DA_ and *N* values are indicated on top. Interpretation of the scattering patterns in real space is schematically given on the bottom, showing the hydrophobic domains in pink.

Figure 3 shows two-dimensional (2D) SAXS patterns of the 64 wt% solutions aligned under oscillatory shear (see Supplementary Figs. 38–S40 for the data at different compositions and *N*). At *i*_DA_ = 60% and *N* = 35, the *L*_α_ phase is evident lacking specific orientation (Fig. 3(a, b)). With increasing *N*, intense spots arise at low *q* followed by higher-order scatterings, signaling the *L*_f_ phase (Fig. 3(c, d)). The strongly aligned scatterings along the meridian axis in both radial and tangential directions correspond to the well-developed stacks of the bilayer-folded lamellae. The intense scattering intensities are also consistent with the discontinuous bilayer structure in the vertical direction rather than undulation. This is distinct from the sawtooth pattern found in the ripple phase (*P*_β’_) produced by in-plane modulation^20^. Scattering features from the hinges are not discernable implying their irregular arrangement. The micellar lamellae exhibit equatorial arcs along the tangential direction as they develop vertically into the bilayer-folded lamellae. TEM investigation also supports the presence of the folded lamellar domains (Supplementary Fig. 41). Consistent with the 1D SAXS data, the (20) peak is split indicating the coexistence of folded and unfolded micellar lamellae; one with longer *d*_m_ is assigned to be folded that disappears in the *L*_α_ phase. The folding is thicker than the *i*_DA_ = 55% case and *d*_f_ exceeds 20 nm at higher concentrations. The backbone is thought to be folded more frequently as the polymer composition becomes more symmetric.

The scattering pattern of the solution with *i*_DA_ = 55% shares most characteristics with the *i*_DA_ = 60% data (Fig. 3(e, f)). The lamellae can unfold under high strain (Supplementary Fig. 42). Interestingly, the (12) scattering spot manifests an out-of-layer correlation between the folded lamellae (Supplementary Fig. 43). It is also possible that herringbone-like arranged bilayers may contribute to the scattering. The *L*_f_ phase from the *i*_DA_ = 60% sample lacks the correlation. When the hydrophobic content is further decreased to *i*_DA_ = 50%, the mean interfacial curvature increases and the *L*_f_ phase coexists with a hexagonal *H*_I_ phase at large *N* of 198 (Fig. 3(g, h)). This supports that the overall composition of the side chains primarily controls the average packing parameter. Considering the small *d*_f_ (~10 nm) and the *d*_f_/*d*_m_ ratio (~1.6), the folded lamellae seem to be thin. Reducing *N* restores the classical *H*_I_ phase, reproducing the dependence of the *L*_f_ phase on *N* (Fig. 3(i, j)). All the solutions converge to the *L*_α_ phase at 80 wt% without folding regardless of *N*, probably to reduce the surface area of the micellar assemblies and maximize interlayer interaction.

### Origin of folding

**Fig. 4:**
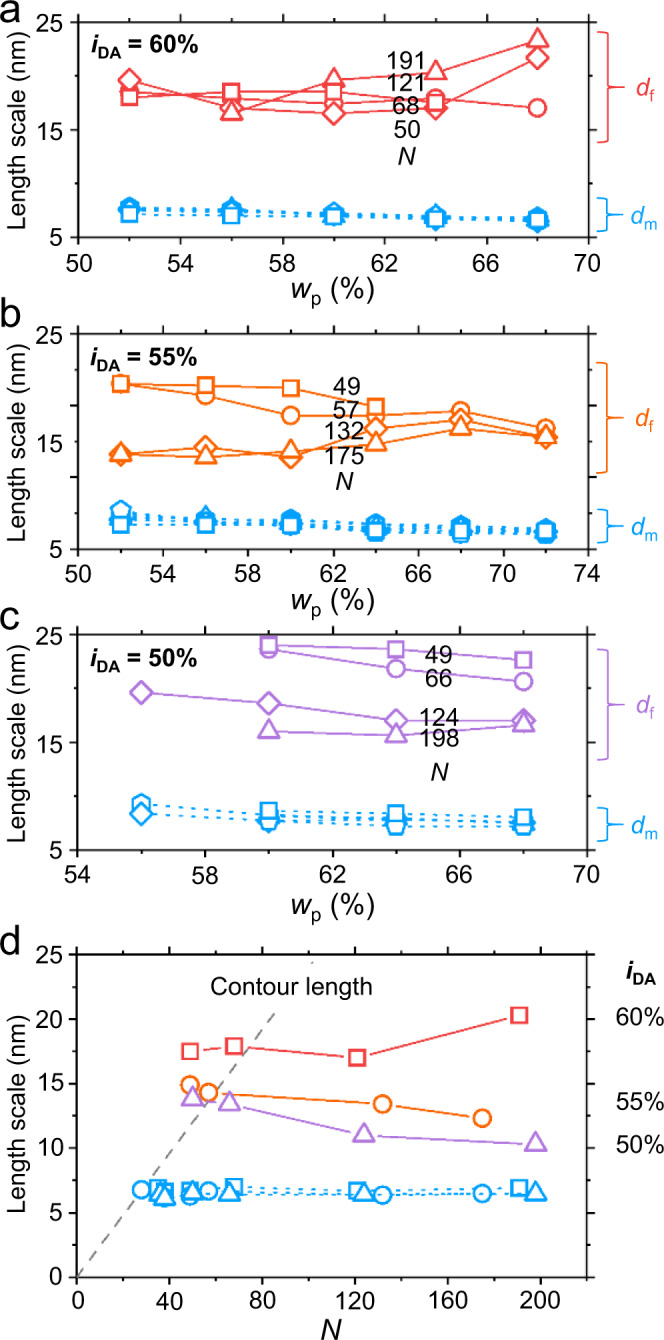
Length scale of folding as a function of *i*_DA_, *w*_p_, and *N*. **a**–**c** Micellar lamellar spacing *d*_m_ and folded lamellar spacing *d*_f_ as a function of *w*_p_. **a**: *i*_DA_ = 60%; **b**: 55%; **c**: 50%. Squares, circles, triangles, and diamonds represent samples with different *N*, in increasing order. **d** *d*_m_ and *d*_f_ as a function of *N* at *w*_p_ = 64%. Contour length is shown as a dashed line. Squares, circles, and triangles denote *i*_DA_ of 60%, 55%, and 50%, respectively.

These scattering features may be compared to binary superlattice systems via multicomponent self-assembly showing two different and crystallographically related length scales^21,22^. We emphasize that the *L*_f_ phase arises from a homogeneous aqueous solution of a single amphiphilic random copolymer, and folding creates a new length scale that does not correlate to the micellar dimension (Fig. 4(a–c)). Compared to the *L*_α,g_ phase in which the poly(oxyethylene)-tethered lipid additive aggregates via in-plane phase separation and stabilizes the high local curvature in the bilayer^15^, a distinct feature of our observation is that the *L*_f_ phase appears as a thermodynamically stable phase without any additive or localized external force. The *L*_f_ phase is even produced when a THF solution of P(DA-*r*-PEGA) is dialyzed against water and concentrated. Mixing the polymers with *i*_DA_ = 50 and 60% in equal amounts does not reproduce the phase behavior of *i*_DA_ = 55% (Supplementary Fig. 44). A gradient copolymer of DA and PEGA synthesized by continuous feeding of DA into the PEGA-containing polymerization mixture self-assembles in a completely different manner, rather resembling PDA-b-PEGA diblock copolymer by showing a strong principal scattering peak at low *q* (Supplementary Figs. 45 and 46 and Supplementary Table 2). Interestingly, when the composition gradient is reduced below a threshold, the side chain-driven self-assembly behavior is restored up to some extent and the *L*_f_ phase appears (Supplementary Fig. 46). The diblock copolymer-like length scaling was also found in the neat copolymers of dodecyl methacrylate (DMA) with PEGA and also DA with oligo(oxyethylene) methacrylate (PEGMA), because the large difference between the reactivity ratios^3,8,18^ produces a strong composition gradient in the sequence (Supplementary Figs. 47 and 48). These characteristics highlight the importance of the sequence distribution of the side chains encoded in the course of polymerization. We speculate that the hydrophilic and hydrophobic side chains should be distributed sufficiently randomly to facilitate the formation of the micellar bilayer, not backbone length-dependent self-assembly. Even when the micellar bilayer can be formed, random heterogeneity in the chain sequence seems to be more effective for the bilayer folding rather than concentrated local curvature mismatches in the gradient sequence.

The amphiphilic random copolymer may be considered a semiflexible chain stiffened by the side chains. Steric congestion between neighboring side chains grafted per every repeating unit elongates the backbone as observed in bottlebrush polymers^23^. This may be related to the preference of the lamellar packing over the hexagonal cylindrical arrangement with increasing *N*. With increasing *N* further, however, entropic loss caused by chain stretching predicts that the backbone should be folded above the critical length to relieve the penalty^24,25^. Such hairpin folding has been observed in main chain liquid crystalline polyesters^25,26^. This picture is consistent with the fully elongated conformation of shorter polymers with lower *N*, which generally develop ordered mesophases at lower concentration but do not show the *L*_f_ phase. We find the average number of hairpins per chain is in the range of 1–4 at *i*_DA_ = 55 and 60%, which is also comparable to the literature^26^. We also note that the amphiphilic random copolymer with the stiffer polymethacrylate backbone did not produce the *L*_f_ phase, despite forming the multilamellar *L*_α_ phase (Supplementary Fig. 49).

We tried to locate the critical *N* that corresponds to the onset of folding. For all three *i*_DA_ fractions we investigated in this study, SAXS features of the *L*_f_ phase started to appear when *N* ~ 50 (Fig. 4d and Supplementary Figs. 50–53). At this critical *N*, the contour length coincides with the doubled persistent length of C12E9 wormlike micelles (~14 nm)^27,28^. Recent bottlebrush simulations suggest that the persistent length of the backbone is comparable to the radius of the bottlebrush^29^. Roughly assuming 0.5 *d*_m_ as the radius, the persistent length of P(DA-*r*-PEGA) corresponds to ~3.5 nm in the disordered state, which would much smaller than that in the micellar state. We interpret that a backbone length above the critical *N* is required to retain the complete reversal of the micellar bilayer direction. Considering the hinges formed by folding to be line defects, we relate the bending rigidity *κ* to the local curvature at the onset of *L*_α _– *L*_f_ transition by equating the Helfrich elastic energy with the entropy gain following the equation given by Warriner et al.:^15^

1 $$\kappa =\frac{\sqrt{2}{k}_{{{{{{\rm{B}}}}}}}T}{\pi {d}_{{{{{{\rm{m}}}}}}}\left(C-{C}_{0}\right)}$$

where *C* (=2/*d*_m_) and *C*_0_ represent the local curvature at the hinge and the mean curvature, respectively. Localization of hydrophobic- and hydrophilic-rich polymer species at the hinges to stabilize the high local curvature is not likely, as their relative populations decrease with increasing *N* in random copolymerization^30^. *C* is weakly sensitive to *N* and *i*_DA_ (Fig. 4).

We propose that DA- and PEGA-rich local sequences are responsible for folding (Fig. 1b). Limited interpair interaction between local sequences has been also attributed to the origin of liquid crystalline ordering in random-sequence DNA oligomer solutions^31^. Assuming recurrent folding, their populations should be balanced to increase the folding density and reduce the persistent length (~*d*_f_). Calculation of the sequence-length distributions^3^ suggests fewer sequence pairs will generate high local curvature, by deviating from the symmetric composition, resulting in the decreased (*C* – *C*_0_). This qualitatively captures the *d*_f_ dependence on *i*_DA_, because *κ* is linearly proportional to the persistent length^27,28^.

## Discussion

The *L*_f_ phase can be readily aligned by shear and accommodate external guests suggesting its use for patterning and templating. We envision that hierarchically structured, creased sheet-like nanomaterials may be prepared based on the *L*_f_ phase with increased surface area, connectivity, and modified mechanical responses^32–34^.

## Methods

### Materials

1-Dodecanthiol, carbon disulfide, potassium phosphate, 2-bromo-2-methylpropionic acid, and acetone were purchased from Sigma-Aldrich (St. Louis, MO) and used for the synthesis of 2-(dodecylthiocarbonothioylthio)-2-methylpropionic acid as a CTA for the RAFT copolymerization of acrylate monomers. The other CTA, 4-cyano-4-[(dodecylsulfanylthiocarbonyl)sulfanyl]pentanoic acid, was purchased from Sigma-Aldrich and used for the RAFT copolymerizations when the copolymerization mixture contained a methacrylate monomer. Azobisisobutyronitrile (AIBN, 98%) was purchased from Junsei (Tokyo, Japan) and purified by recrystallization in methanol. Poly(ethylene glycol) methyl ether acrylate (PEGA), dodecyl acrylate (DA), poly(ethylene glycol) methyl ether methacrylate (PEGMA), and dodecyl methacrylate (DMA) were purchased from Sigma-Aldrich and TCI (Tokyo, Japan). They were purified by passing through a basic alumina column prior to polymerization. HPLC grade toluene was purchased from Burdick & Jackson (Morristown, NJ) and used as a polymerization solvent after purification using a solvent purification system (C&T International, Suwon, Korea).

### Synthesis of 2-(dodecylthiocarbonothioylthio)-2-methylpropionic acid

The compound was synthesized according to a procedure reported in the literature^35^. A solution of potassium phosphate was prepared in 1 L three-necked round bottom flask with acetone under nitrogen atmosphere. 1-Dodecanthiol (20.2 g, 100 mmol) and carbon disulfide (20.6 g, 270 mmol) were added in the flask and stirred at room temperature for 20 min. A solution of 2-bromo-2-methylpropionic acid (15.0 g, 90 mmol) in acetone was added to the mixture and stirred at room temperature for 16 h. After removing the solvent using rotary evaporator, the mixture was extracted with water and dichloromethane. The mixture was purified by recrystallization in hexane. The product was obtained by evaporating the solvent under reduced pressure (27.2 g, 80%). Supplementary Fig. 2 shows a ^1^H nuclear magnetic resonance (NMR) spectrum of the resulting product. ^1^H NMR (400 MHz, CDCl_3_): *δ* 3.31–3.22 (m, 2H), 1.71 (s, 6H), 1.65 (t, *J* = 7.4 Hz, 2H), 1.35 (d, *J* = 7.5 Hz, 2H), 1.23 (s, 16H), 0.91–0.81 (m, 3H).

### RAFT copolymerization of PEGA and DA

Synthetic procedure for P(DA_96_-*r*-PEGA_79_) with *i*_DA_ = 55% is given as an example. A homogeneous solution of DA (7.93 g, 33.0 mmol), PEGA (13.0 g, 27.0 mmol), CTA (0.12 g, 0.33 mmol) and AIBN (0.0054 g, 0.033 mmol) was prepared in toluene (25 mL). The mixture was then transferred into an ampoule, degassed via three cycles of freeze-pump-thaw, and flame-sealed under vacuum. After heating at 60 °C for 12 h, the ampoule was cooled to room temperature and then opened to stop the polymerization. The mixture was dialyzed against methanol using a Spectra/por membrane (Spectrum, CA) with a molecular weight cutoff of 3.5 kg mol^−1^. The product was obtained by evaporating the solvent under reduced pressure (18.0 g, 86%). Supplementary Fig. 3 shows ^1^H NMR spectrum of the resulting polymer. Degree of polymerization (*N*) was calculated by conversion of each monomer determined by ^1^H NMR spectroscopy.

P(DA-*r*-PEGA)s with different *N* and compositions were synthesized by varying the [monomer]:[CTA] ratio and also the [DA]:[PEGA] composition in the feed. Supplementary Figs. 4 and 5 show their ^1^H NMR spectra and SEC traces, respectively. Supplementary Table 1 summarizes characterization details of the synthesized polymers in this study. For P(DA_62_-*r*-PEGA_62_), we monitored the polymerization kinetics by ^1^H NMR spectroscopy (Supplementary Fig. 6). Identical consumption rates were observed for both DA and PEGA following a first-order kinetics, supporting random copolymerization behavior in a controlled manner via RAFT mechanism.

### RAFT copolymerization of PEG(M)A and D(M)A

Synthetic procedure for P(DMA_106_-*r*-PEGMA_86_) with *i*_DMA_ = 55% is given as an example. A homogeneous solution of DMA (2.45 g, 9.63 mmol), PEGMA (3.94 g, 7.88 mmol), CTA (0.034 g, 0.083 mmol) and AIBN (0.0013 g, 0.0082 mmol) was prepared in toluene (5 mL). The identical polymerization and workup procedures described above were employed to yield the product (5.7 g, 89%). P(DMA-*co*-PEGA) and P(DA-*co*-PEGMA) were synthesized in the same manner, and their characterization data are presented in Supplementary Figs. 7 and 8 and Table 1.

### Gradient copolymerization of PEGA and DA

Synthetic procedure for P(DA_91_-*grad*-PEGA_92_) (0:100) is given as an example. A homogeneous solution of PEGA (7.58 g, 15.8 mmol), CTA (0.06 g, 0.17 mmol) and AIBN (0.0027 g, 0.017 mmol) was prepared in toluene (13 mL). The mixture was then transferred into a Schlenk flask. After 1 h of argon purging, the mixture was heated to 60 °C. Then DA (4.63 g, 19.3 mmol) was continuously injected into the mixture by syringe at a rate of 0.01 mL min^−1^. After 12 h, the flask was cooled to room temperature and then opened to stop the polymerization. The product was precipitated in hexane and obtained by evaporating the solvent under reduced pressure (9.9 g, 81%).

P(DA-*grad*-PEGA)s with different extents of the composition gradient were synthesized by varying the composition of the initial polymerization mixture and also the feed. Synthetic details are summarized in Supplementary Table 2, along with their characterization data in Supplementary Fig. 45.

### Characterization

^1^H nuclear magnetic resonance (NMR) spectroscopy was performed on a Bruker Avance 400 MHz spectrometer (Billerica, MA) using CDCl_3_ as the solvent. The residual NMR solvent peak was used as an internal reference. Size exclusion chromatography (SEC) was performed in *N*,*N*-dimethylformamide (DMF) containing 0.05 M of LiBr at 45 °C with a flow rate of 1 mL min^−1^ on an Agilent 1260 Infinity system (Santa Clara, CA). The instrument is equipped with a 1260 refractive index detector. A PSS GRAM (Mainz, Germany) analytical 100 Å column with a molar mass range 300–60,000 g mol^–1^ and two PSS GRAM analytical 10,000 Å columns with a molar mass range 10,000–50,000,000 g mol^–1^ are used in series. The molar masses of the polymers were calculated relative to linear poly(methyl methacrylate) (PMMA) standards obtained from Agilent Technologies.

### Solution preparation

Homogeneous aqueous solutions of P(DA-*r*-PEGA) were prepared in deionized water at a concentration range of 40–80% by polymer weight. The solutions were mixed on a vortex machine for 5 min, and then kept at 60 °C overnight.

### X-ray scattering measurements

Small- and wide-angle X-ray scattering (SAXS/WAXS) measurements were performed at the 9A U-SAXS beamline at Pohang Accelerator Laboratory (PAL). For transmission SAXS measurements, wavelength (*λ*) of 0.621 Å (19.95 keV) and sample-to-detector distance (SDD) of 2.0 m were used. P(DA-*r*-PEGA) solutions sealed in hermetic DSC pans were exposed to X-ray. Scattering intensity was monitored by a Rayonix SX165 CCD detector with 2048 × 2048 pixels. The two-dimensional scattering patterns were azimuthally integrated to afford one-dimensional profiles presented as scattering vector (*q*) versus scattered intensity, where the magnitude of scattering vector is given by *q* = (4*π*/*λ*)sin *θ*. Scattering angles were corrected by the scattering peak from a pre-calibrated SEBS specimen as a reference. Temperature was varied by using a heating sample stage equipped with a Lakeshore temperature controller in the range of 25 °C to 60 °C.

For transmission WAXS measurements, *λ* of 0.621 Å (19.79 keV) and SDD of 0.23 m were used. P(DA-*r*-PEGA) solutions loaded into 1.5 mm quartz capillaries (Hampton Research, CA) were exposed to X-ray in a vacuum chamber. Scattering intensity was monitored by a Rayonix SX165 CCD detector with 2048 × 2048 pixels. The two-dimensional scattering patterns were azimuthally integrated to afford one-dimensional profiles presented as *q*. Scattering angles were corrected by the scattering peak from a pre-calibrated sucrose specimen as a reference.

For the SAXS measurements under an oscillatory shear flow, *λ* of 0.621 Å (19.95 keV) and SDD of 2.0 m were used. An Anton Paar rheometer Model MCR 502 (Graz, Austria) with a polycarbonate cup was used to apply the oscillatory shear. The polycarbonate coquette cell has an outer diameter of 11.0 mm, and the outer stator has an inner diameter of 10.0 mm to a shear gap of 1.0 mm. The scattering patterns from sheared samples from radial and tangential direction were measured by varying strain (*γ*) from 0% to 1000% with angular frequency (*ω*) of 10 rad/s.

### Differential scanning calorimetry (DSC)

Thermal transitions of P(DA-*r*-PEGA) solutions were investigated on a TA Instruments DSC Q20 (New Castle, DE) using a scan rate of 10 °C/min under N_2_ atmosphere. Thermograms were recorded during the second heating cycle in the temperature range of −60 to 70 °C

### Cryo-transmission electron microscopy (cryo-TEM)

TEM specimens were prepared as follows. The P(DA-*r*-PEGA) solution was sheared by 20G syringe at room temperature, and then quickly quenched in liquid nitrogen for 10 s. Leica EM UC7 ultramicrotome (Vienna, Austria) was used to section the vitrified solution, which was equipped with a glass knife at a cutting rate of 3 mm/s. The block face for the sample preparation was about 200 × 200 µm. The ultrathin sections (~50 nm thick) at −70 to −68 °C were transferred to formvar/carbon-coated copper grid 200 mesh (Electron Microscopy Sciences, Hatfield, PA)^36^. In order to improve mass contrast, the thin films were exposed to staining agent vapor (0.5 wt% aqueous RuO_4_) vapor for 1 h. The specimen was observed with a JEM-2010 (JEOL Ltd., Tokyo, Japan) operating at 200 kV and the images were taken by a bottom-mounted Tangra CCD camera (Olympus Soft Imaging Solutions, Münster, Germany). The data were analyzed with imaging software RADIUS (Olympus Soft Imaging Solutions, Münster, Germany).

### Dynamic oscillatory shear measurement

The oscillatory shear measurements were performed using an Anton Paar rheometer Model MCR 302 with a plate and plate geometry. The volume of the loaded sample was 0.5 mL, and the gap between the plates was 0.5 mm. Storage (*G*’) and loss (*G*”) moduli were measured in the *ω* range of 0.1–100 rad s^−1^ at *γ* = 1%. Strain sweep measurements were performed by varying *γ* from 0.01% to 1%.

### Polarized optical microscopy analysis

Pristine glass substrates (25 × 37.5 mm^2^) were cleaned with acetone, ethanol, and deionized water to remove any impurities. P(DA-*r*-PEGA) solutions were loaded on the glass and covered with another glass to spread samples uniformly. The samples were aligned by applying a shear force to sandwiched cells in one direction. The optical texture of the samples was observed with a Nikon LV100POL polarized optical microscope (Nikon, Japan) with a full-wave plate (*λ* = 530 nm) inserted at 45° with respect to the polarizers. A Nikon DS‐Ri1 CCD camera was used to record the images. Temperature was controlled by using a Linkam LTS420 heating stage and a TMS94 temperature controller (Linkam Scientific, UK). To observe spherulite formation, the samples were heated to 65 °C to reach the isotropic phase. After keeping at 65 °C for about 5 min, the samples were then slowly cooled to 25 °C with the rate of 0.5 °C/min.

## Supplementary information

Supplementary Information

## Data Availability

All data are available in the main text or the Supplementary Materials.
