# Supplementary material for: Bilayer-folded lamellar mesophase induced by random polymer sequence
Source: Nat Commun. 2022 May 4;13:2433. doi: 10.1038/s41467-022-30122-z (PMC9068626; doi:10.1038/s41467-022-30122-z)
Supplement: Supplementary file 1 — Supplementary Information [file 41467_2022_30122_MOESM1_ESM.pdf]

Supplementary Information

Bilayer-folded lamellar mesophase induced by random polymer sequence

Minjoong Shin,[1] *Hayeon Kim,*[2] *Geonhyeong Park,*[1] *Jongmin Park,*[1] *Hyungju Ahn,*[3] *Dong Ki Yoon,*[1,4,5] *Eunji Lee,*[2] *Myungeun Seo*[1,5]*

[1]Department of Chemistry, Korea Advanced Institute of Science and Technology (KAIST); Daejeon 34141, Republic of Korea;

[2] School of Materials Science and Engineering, Gwangju Institute of Science and Technology (GIST); Gwangju 61005, Republic of Korea;

[3] Pohang Accelerator Laboratory (PAL); Pohang 37673, Republic of Korea;

[4] Graduate School of Nanoscience and Technology, KAIST; Daejeon 34141, Republic of Korea;

[5] KAIST Institute for the Nanocentury, KAIST; Daejeon 34141, Republic of Korea;

*To whom correspondence should be addressed.

E-mail: seomyungeun@kaist.ac.kr

This PDF file includes:

Supplementary Figs. 1 – 53

Supplementary Tables 1 – 2

S1. Polymer Synthesis

Supplementary Table 1. Characterization of P(DA-*r*-PEGA) and P(D(M)A-*co*-PEG(M)A)s

P(DA-*r*-PEGA)							
Sample name	Feed composition [DA]:[PEGA]	Conv[a].$_{DA}$ (%)	Conv[a].$_{PEGA}$ (%)	$M_{n,theo}$[a] (kg/mol)	i_{DA}[b] (mol %)	$M_{n,SEC}$[c] (kg/mol)	*Đ*
P(DA_{21}-*r*-$PEGA_{14}$)	[21]:[14]	98	98	12	60	9	1.26
P(DA_{29}-*r*-$PEGA_{20}$)	[30]:[20]	98	99	17	60	14	1.30
P(DA_{41}-*r*-$PEGA_{27}$)	[42]:[28]	97	96	23	60	16	1.23
P(DA_{73}-*r*-$PEGA_{48}$)	[84]:[56]	87	88	41	61	28	1.25
P(DA_{115}-*r*-$PEGA_{76}$)	[126]:[84]	91	90	64	60	41	1.41
P(DA_{16}-*r*-$PEGA_{12}$)	[17]:[13]	96	96	12	55	7	1.28
P(DA_{21}-*r*-$PEGA_{17}$)	[22]:[18]	96	97	14	55	11	1.28
P(DA_{27}-*r*-$PEGA_{21}$)	[28]:[22]	98	99	18	55	14	1.31
P(DA_{31}-*r*-$PEGA_{26}$)	[33]:[27]	95	95	24	55	14	1.20
P(DA_{73}-*r*-$PEGA_{59}$)	[77]:[63]	95	95	46	55	28	1.24
P(DA_{96}-*r*-$PEGA_{79}$)	[99]:[81]	97	97	74	55	33	1.29
P(DA_{18}-*r*-$PEGA_{18}$)	[18]:[18]	98	98	13	51	9	1.20
P(DA_{25}-*r*-$PEGA_{25}$)	[25]:[25]	98	99	18	50	15	1.34
P(DA_{33}-*r*-$PEGA_{33}$)	[35]:[35]	96	95	26	50	16	1.16
P(DA_{62}-*r*-$PEGA_{62}$)	[70]:[70]	89	89	51	50	26	1.20
P(DA_{99}-*r*-$PEGA_{99}$)	[105]:[105]	94	94	76	50	38	1.26
P(D(M)A-*co*-PEG(M)A)							
Sample name	Feed composition [D(M)A]:[PEG(M)A]	Conv[a].$_{D(M)A}$ (%)	Conv[a].$_{PEG(M)A}$ (%)	$M_{n,theo}$[a] (kg/mol)	i_{DA}[b] (mol %)	$M_{n,SEC}$[c] (kg/mol)	*Đ*
P(DMA_{106}-*r*-$PEGMA_{86}$)	[116]:[95]	91	91	70	55	61	1.63
P(DMA_{144}-*co*-$PEGA_{84}$)	[116]:[95]	98	88	67	65	47	1.47
P(DA_{106}-*co*-$PEGMA_{95}$)	[116]:[95]	91	99	73	53	63	2.07

[a]Determined by ^{1}H NMR spectroscopy

[b]Determined by ^{1}H NMR spectroscopy

[c]Determined by SEC analysis based on linear PMMA standards using DMF (LiBr) as an eluent

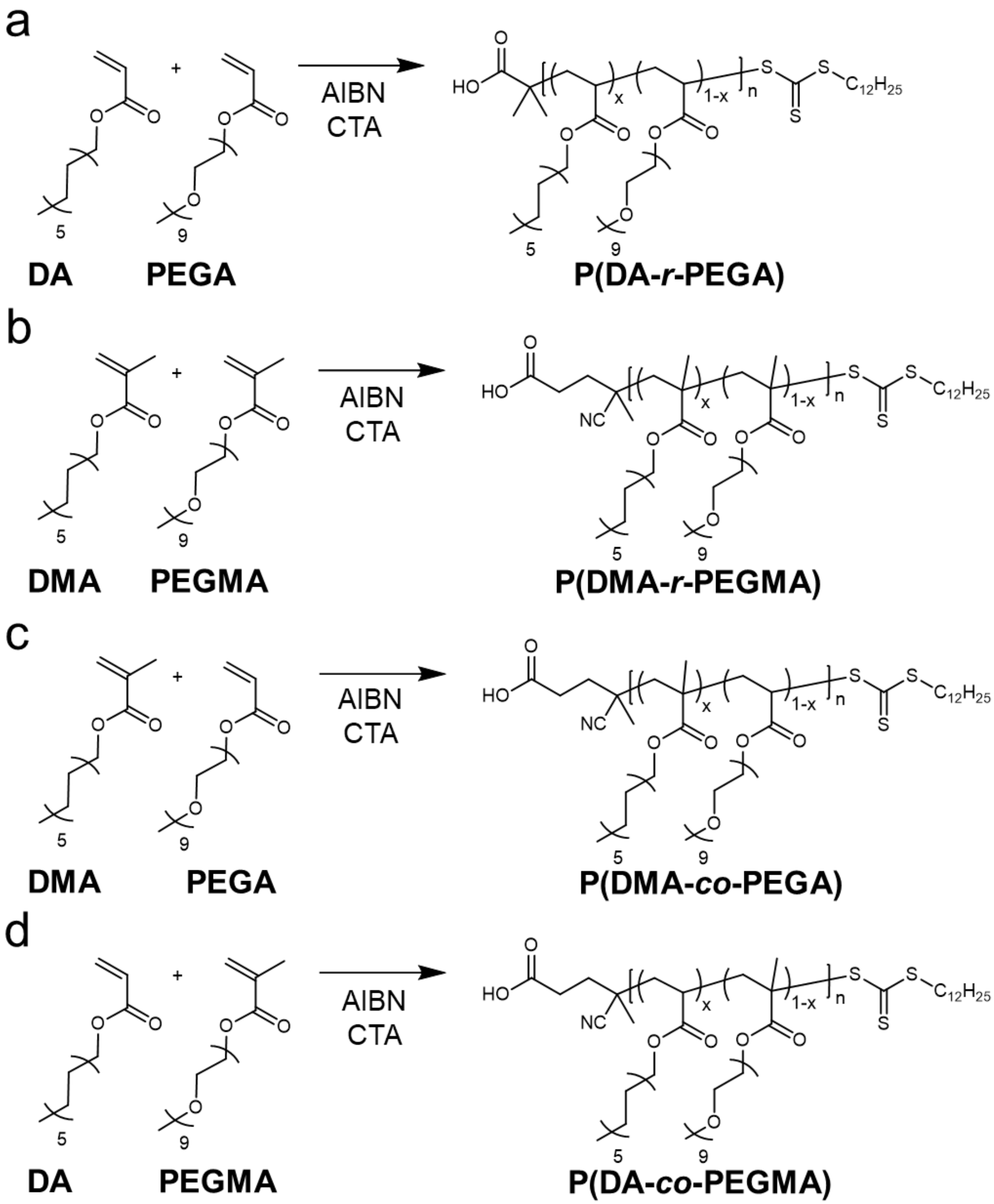

Supplementary Fig. 1. Synthetic routes to amphiphilic copolymers synthesized in this study. (a) P(DA-*r*-PEGA). (b) P(DMA-*r*-PEGMA). (c) P(DMA-*co*-PEGA). (d) P(DA-*co*-PEGMA).

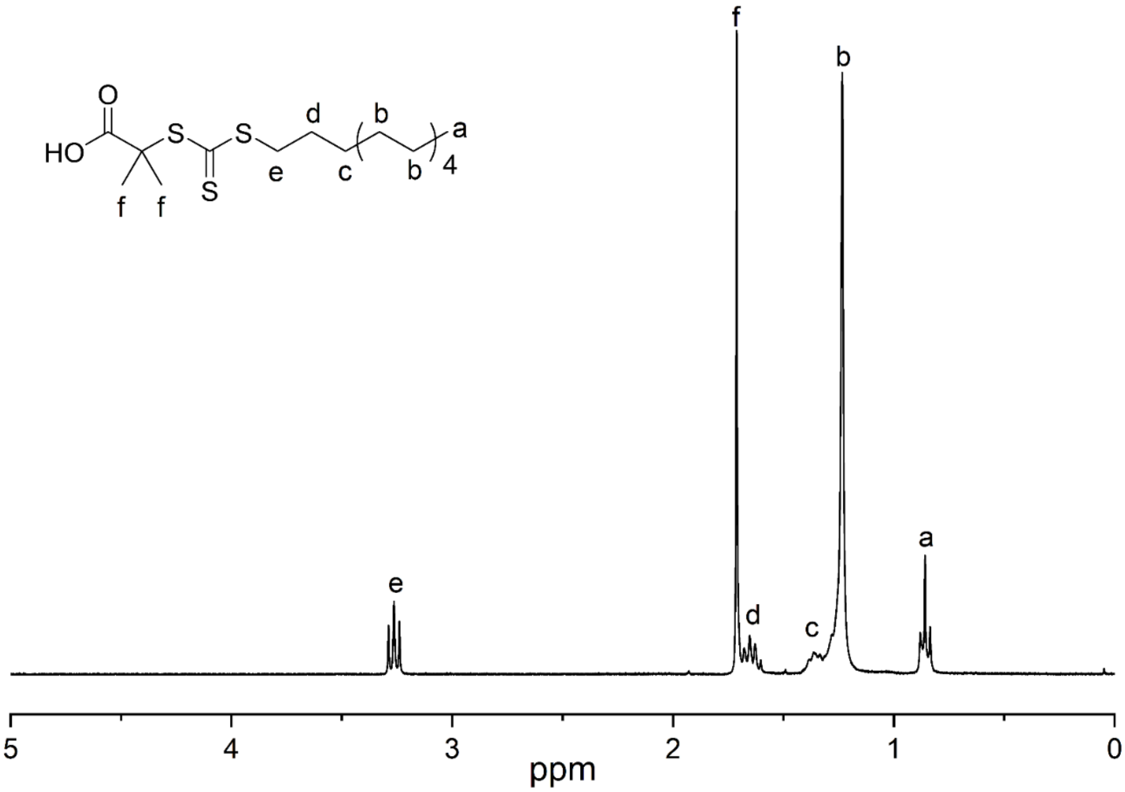

Supplementary Fig. 2. ^{1}H NMR spectrum of 2-(dodecylthiocarbonothioylthio)-2-methylpropionic acid (400 MHz, $CDCl_3$, 20 °C).

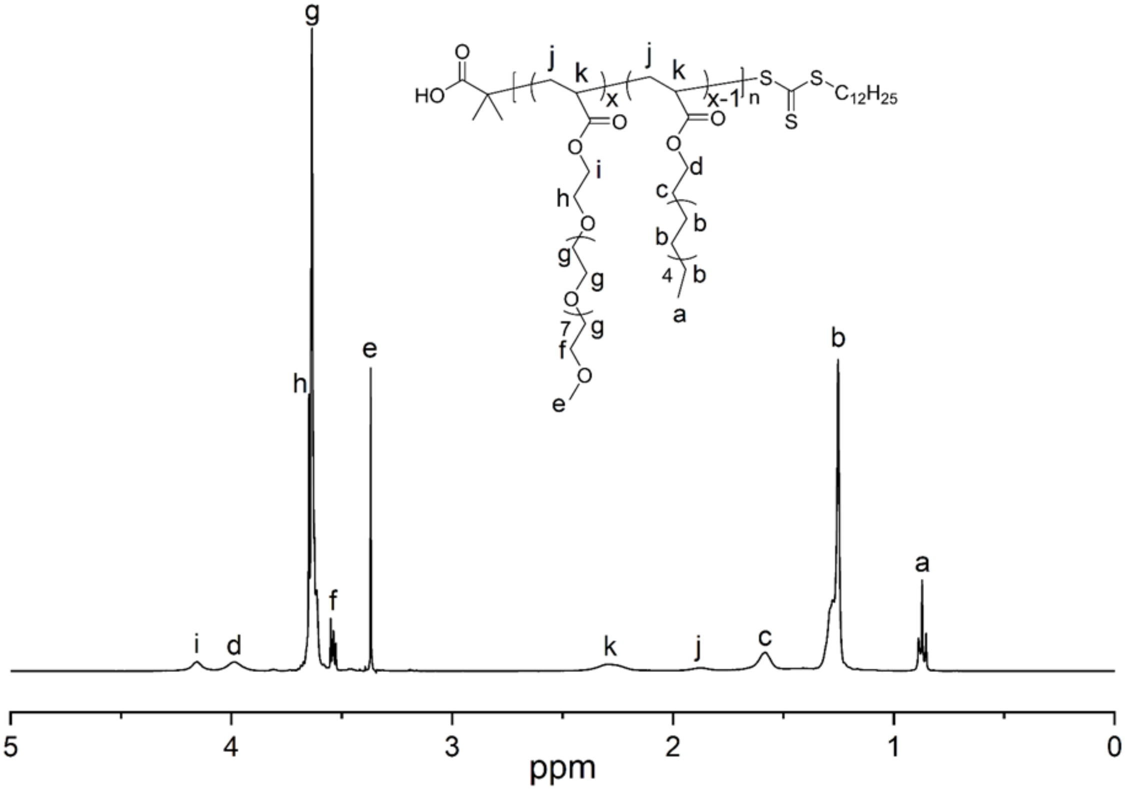

Supplementary Fig. 3. Representative ^{1}H NMR spectrum of P(DA_{96}-*r*-$PEGA_{79}$) with i_{DA} = 55% (400 MHz, $CDCl_3$, 20 °C).

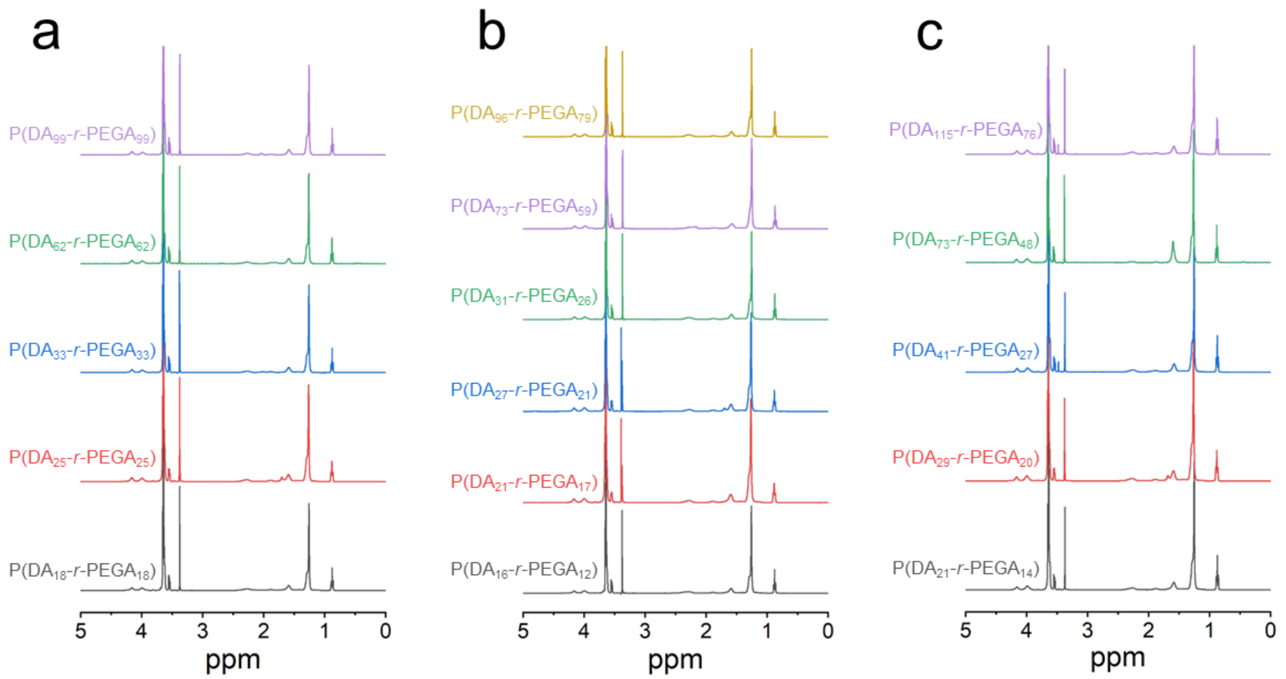

Supplementary Fig. 4. ^{1}H NMR spectra of P(DA$_x$-*r*-PEGA$_y$)s (400 MHz, $CDCl_3$, 20 °C). (a) i_{DA} = 50%. (b) i_{DA} = 55%. (c) i_{DA} = 60%.

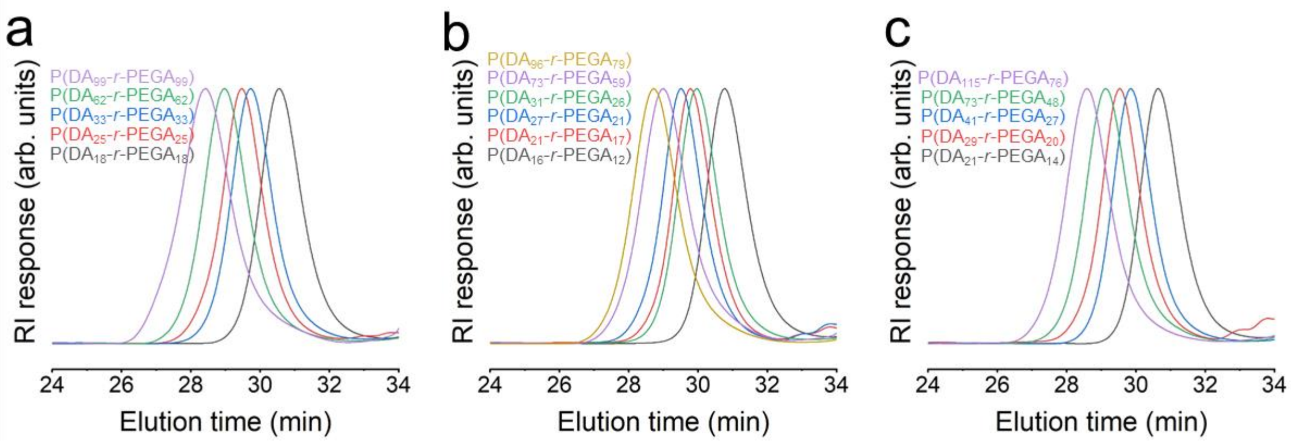

Supplementary Fig. 5. SEC traces of P(DA$_x$-*r*-PEGA$_y$)s (DMF with 0.05 M LiBr, 1 mL min^{-1}, 45 °C). (a) i_{DA} = 50%. (b) i_{DA} = 55%. (c) i_{DA} = 60%.

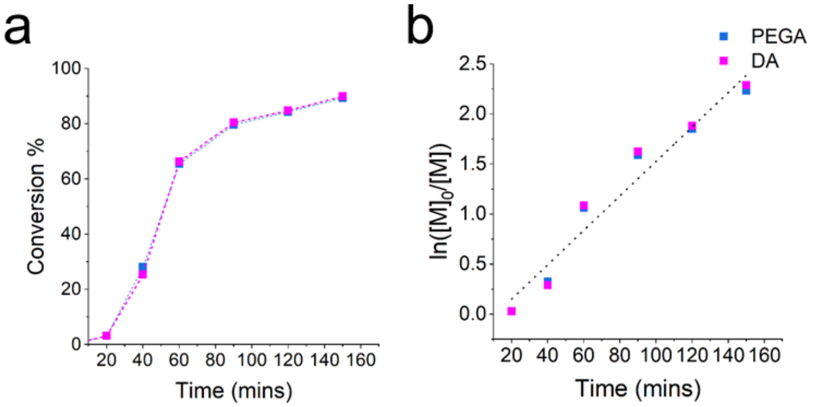

Supplementary Fig. 6. Copolymerization kinetics of DA and PEGA for P(DA$_{62}$-*r*-PEGA$_{62}$). (a) Conversion over time. (b) First-order kinetic plot.

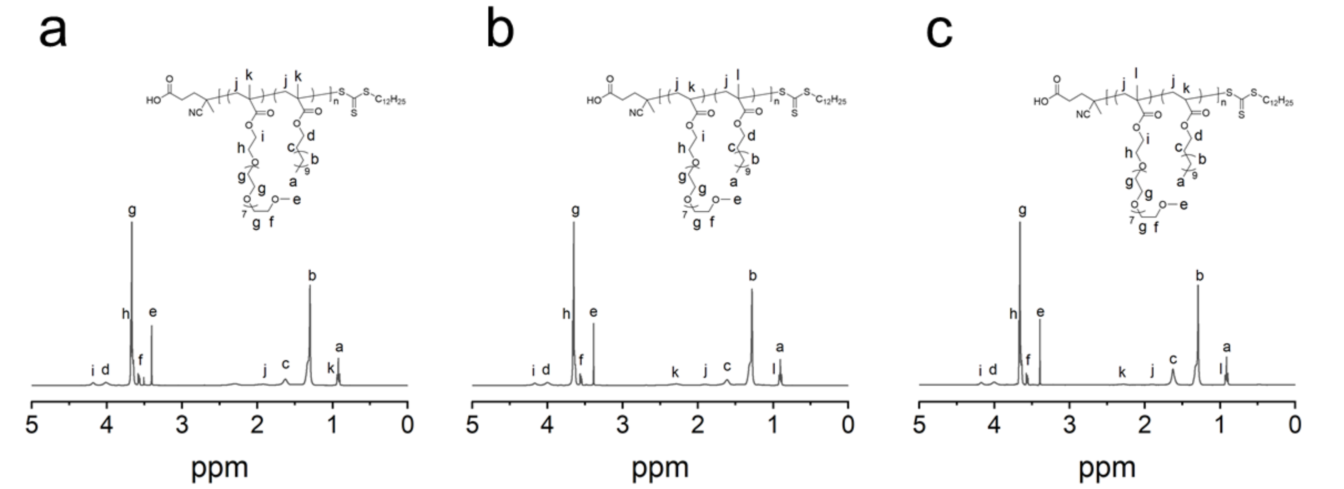

Supplementary Fig. 7. ^{1}H NMR spectra of P(D(M)A_x-*co*-PEG(M)A_y) (400 MHz, $CDCl_3$, 20 °C). (a) P(DMA_{106}-*r*-$PEGMA_{86}$). (b) P(DMA_{144}-*co*-$PEGA_{84}$). (c) P(DA_{106}-*co*-$PEGMA_{95}$).

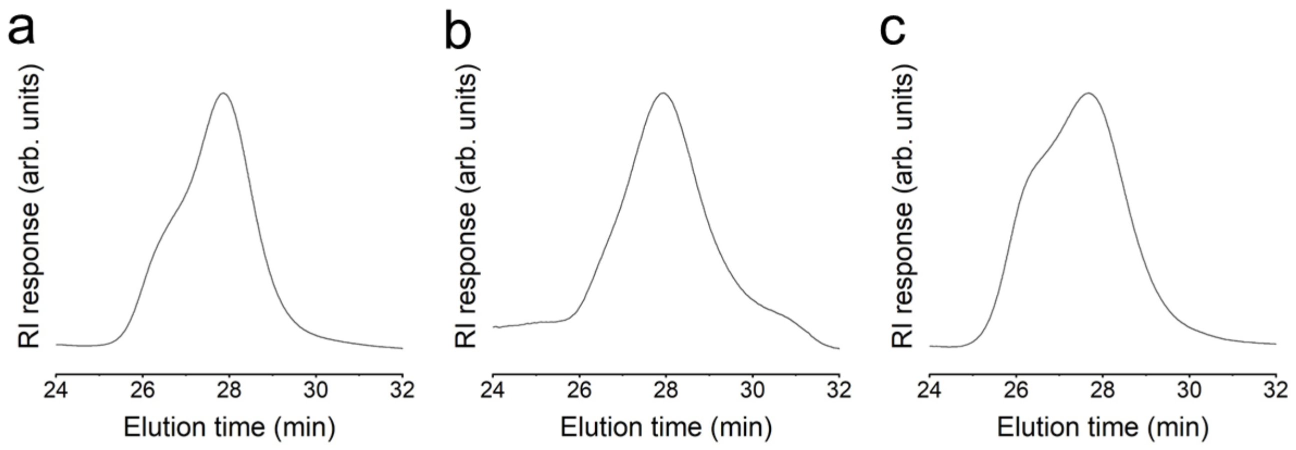

Supplementary Fig. 8. SEC traces of P(D(M)A_x-*co*-PEG(M)A_y)s (DMF with 0.05 M LiBr, 1 mL min^{-1}, 45 °C). (a) P(DMA_{106}-*r*-$PEGMA_{86}$). (b) P(DMA_{144}-*co*-$PEGA_{84}$). (c) P(DA_{106}-*co*-$PEGMA_{95}$).

S2.1. Aqueous solutions of P(DA-*r*-PEGA)s with i_{DA} = 55%

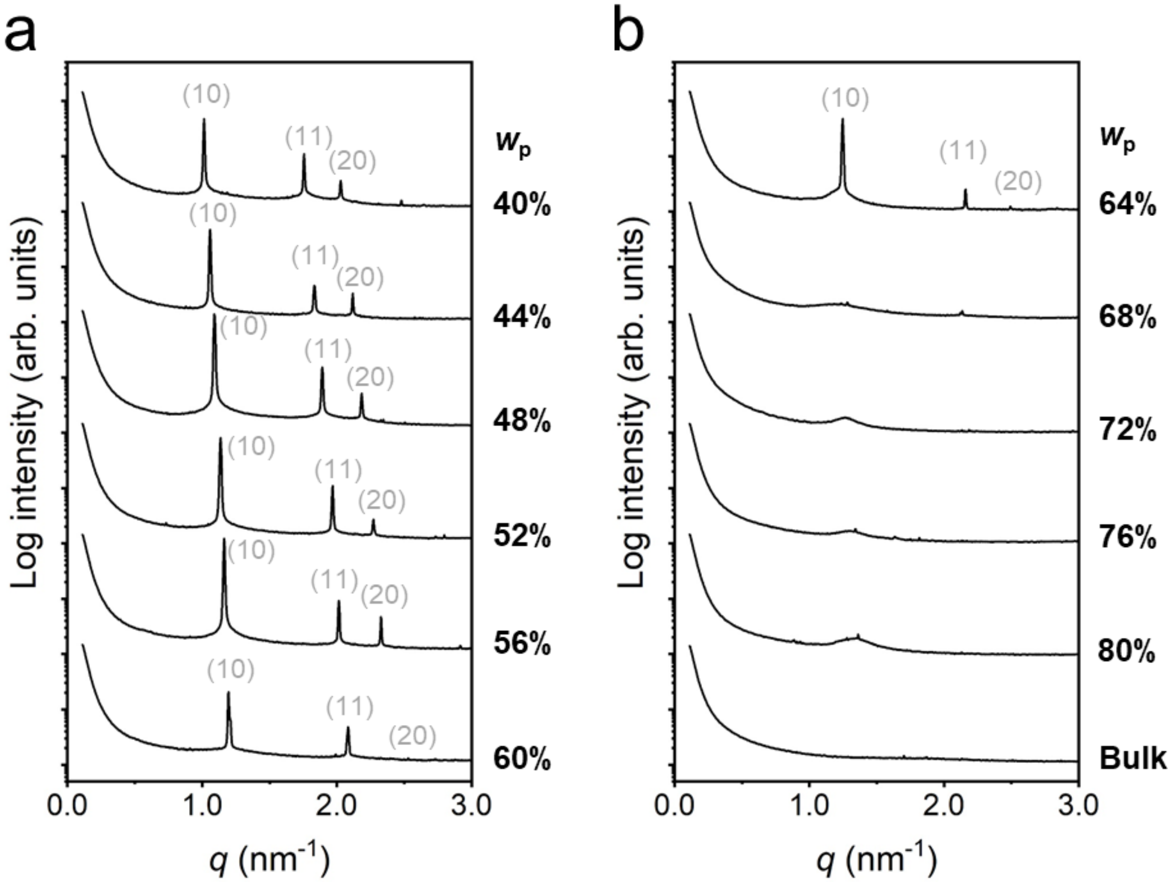

Supplementary Fig. 9. 1D SAXS data of C12E9 aqueous solutions at different concentrations. (a) 40 to 60 wt%. (b) 64 to 100 wt% (bulk). Miller indices for the following unit cell structure are assigned to the selected peaks in the plot: H_I (hexagonal, grey).

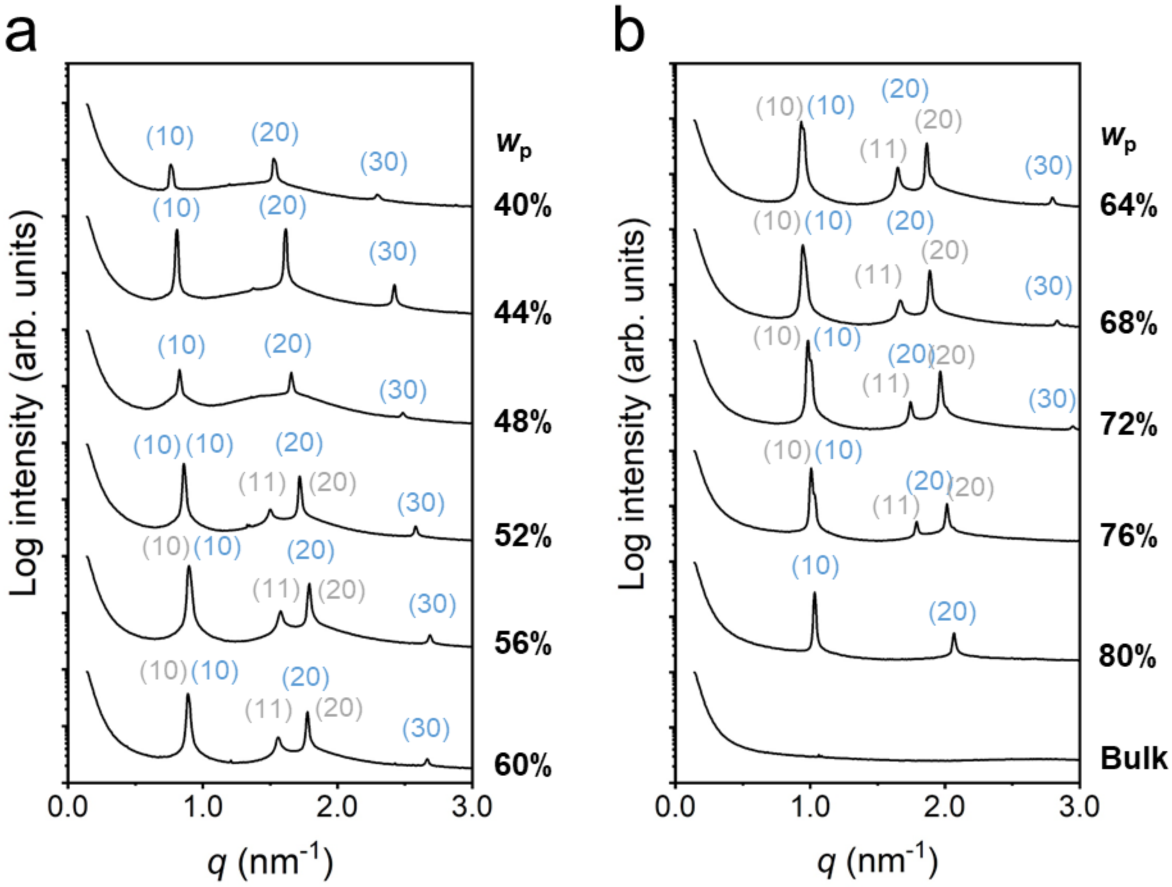

Supplementary Fig. 10. 1D SAXS data of P(DA$_{16}$-*r*-PEGA$_{12}$) (i_{DA} = 55%, N = 28) aqueous solutions at different concentrations. (a) 40 to 60 wt%. (b) 64 to 100 wt% (bulk). Miller indices for the following unit cell structure are assigned to the selected peaks in the plot: L_a (fluidic multilamellar, blue); H_I (hexagonal, grey).

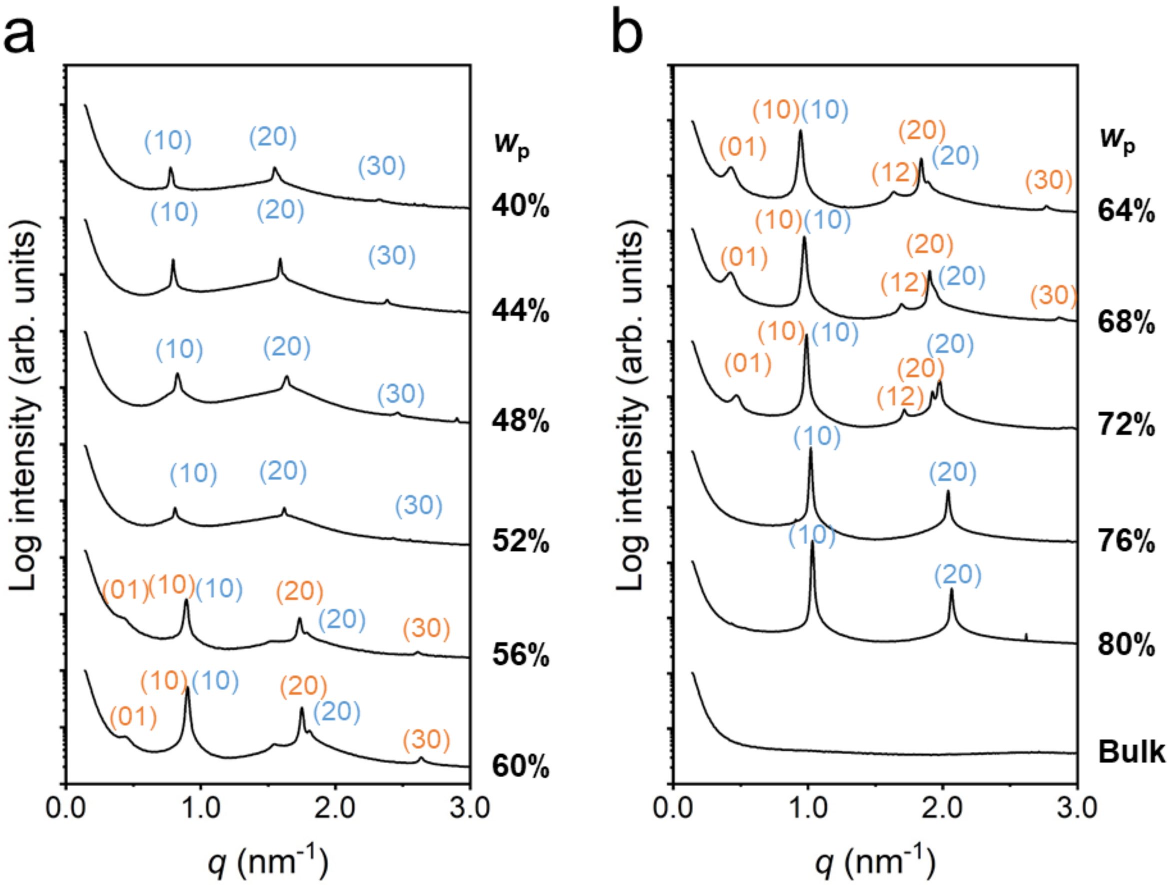

Supplementary Fig. 11. 1D SAXS data of P(DA_{31}-*r*-$PEGA_{26}$) (i_{DA} = 55%, N = 57) aqueous solutions at different concentrations. (a) 40 to 60 wt%. (b) 64 to 100 wt% (bulk). Miller indices for the following unit cell structure are assigned to the selected peaks in the plot: L_a (fluidic multilamellar, blue); L_f (bilayer-folded lamellar, orange).

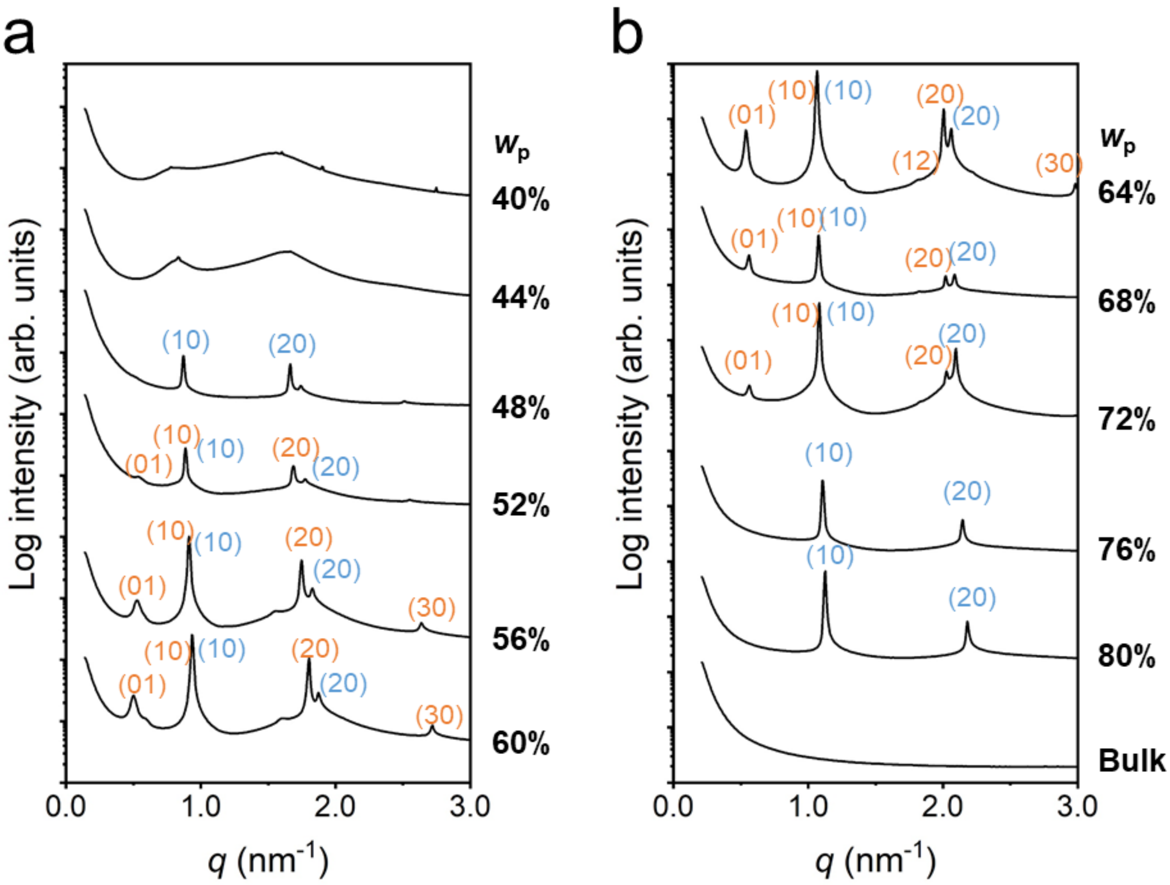

Supplementary Fig. 12. 1D SAXS data of P(DA_{73}-*r*-$PEGA_{59}$) (i_{DA} = 55%, *N* = 132) aqueous solutions at different concentrations. (a) 40 to 60 wt%. (b) 64 to 100 wt% (bulk). Miller indices for the following unit cell structure are assigned to the selected peaks in the plot: L_a (fluidic multilamellar, blue); L_f (bilayer-folded lamellar, orange).

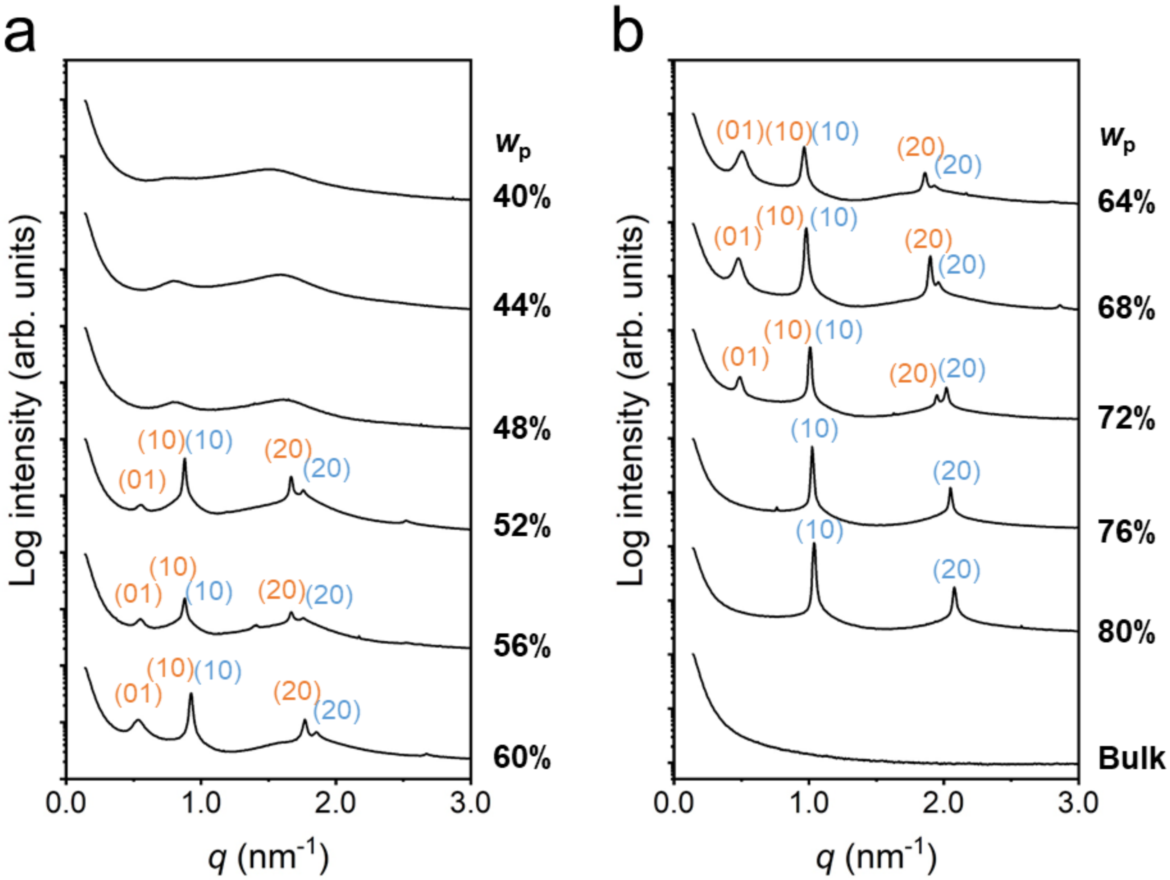

Supplementary Fig. 13. 1D SAXS data of P(DA$_{96}$-*r*-PEGA$_{79}$) (i_{DA} = 55%, N = 175) aqueous solutions at different concentrations. (a) 40 to 60 wt%. (b) 64 to 100 wt% (bulk). Miller indices for the following unit cell structure are assigned to the selected peaks in the plot: L_a (fluidic multilamellar, blue); L_f (bilayer-folded lamellar, orange).

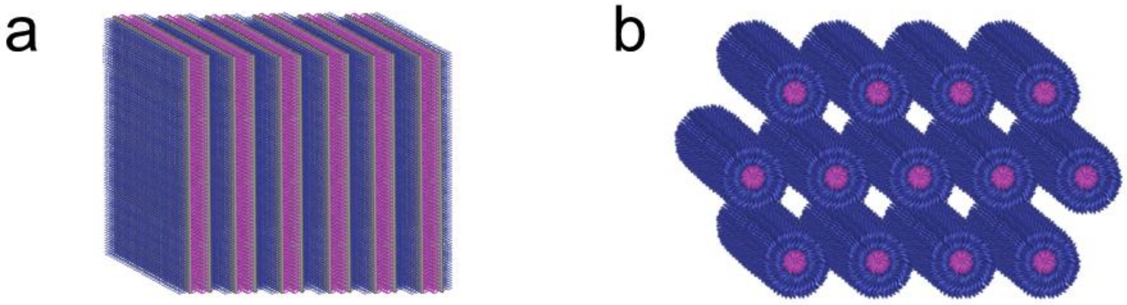

Supplementary Fig. 14. Schematic depictions of the molecular packing found in the $L_α$ (a) and H_I (b) phases.

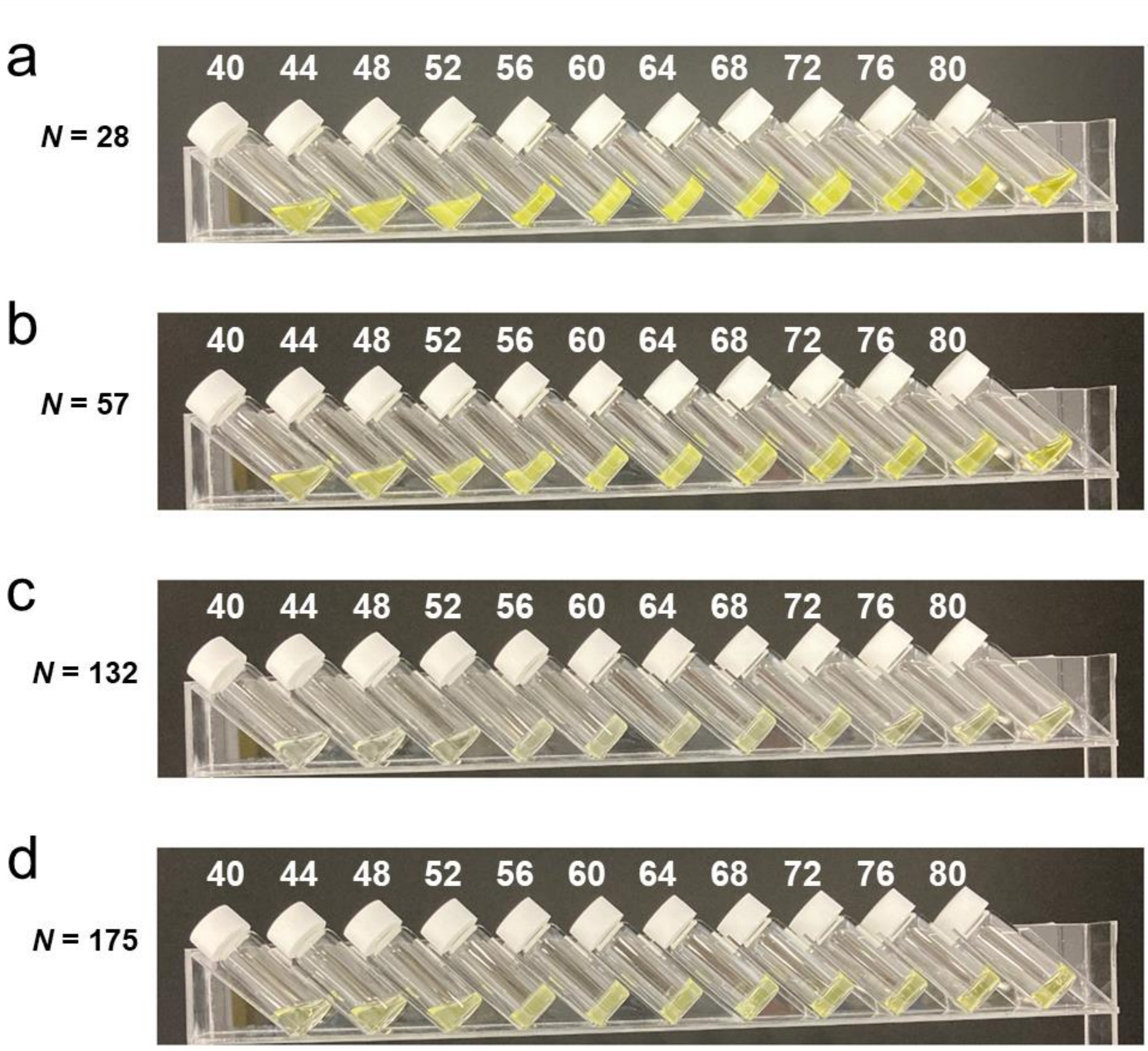

Supplementary Fig. 15. Photos of aqueous solutions of P(DA-*r*-PEGA) with i_{DA} = 55% at room temperature. (a) N = 28. (b) N = 57. (c) N = 132. (d) N = 175. The concentration range for the gelation determined by the inverted vial method was 56 to 72 wt%.

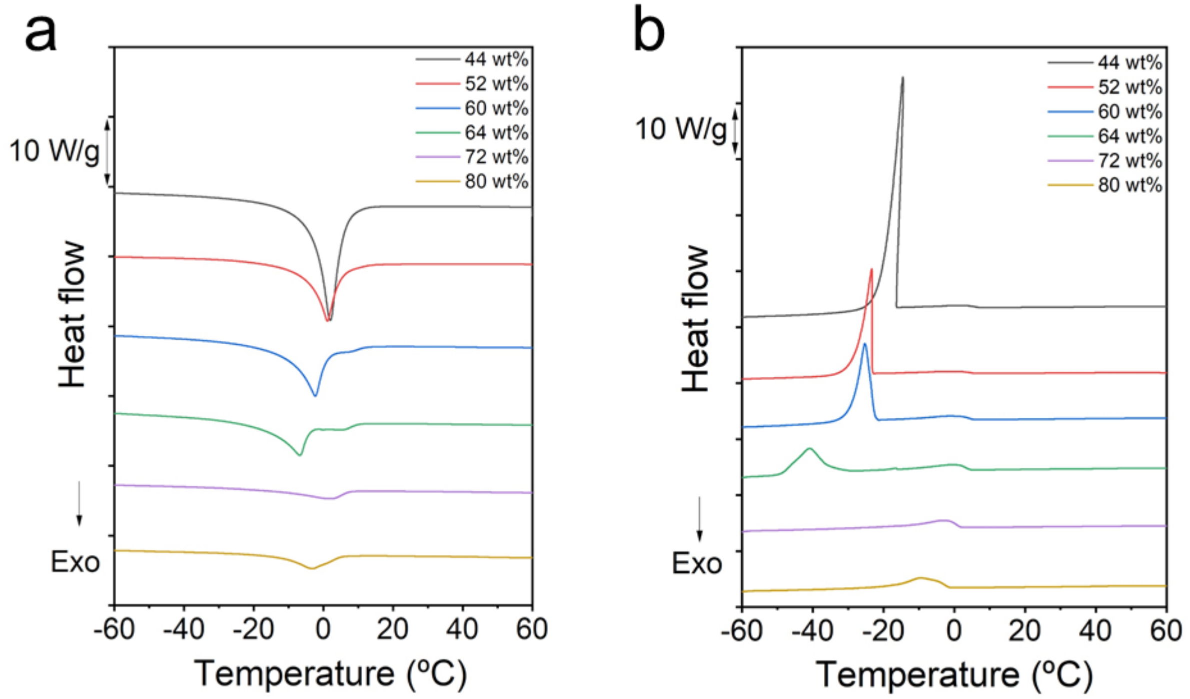

Supplementary Fig. 16. DSC thermograms of aqueous solutions of P(DA-*r*-PEGA) with i_{DA} = 55% and N = 175 during the second heating/cooling cycle. (a) Heating. (b) Cooling. In the cooling scan, the strong exothermic transition below -10 °C was assigned to the crystallization of the PEGA side chains swollen with water. The relatively weak transition appearing around 0 °C was attributed to the solidification of the DA side chains.

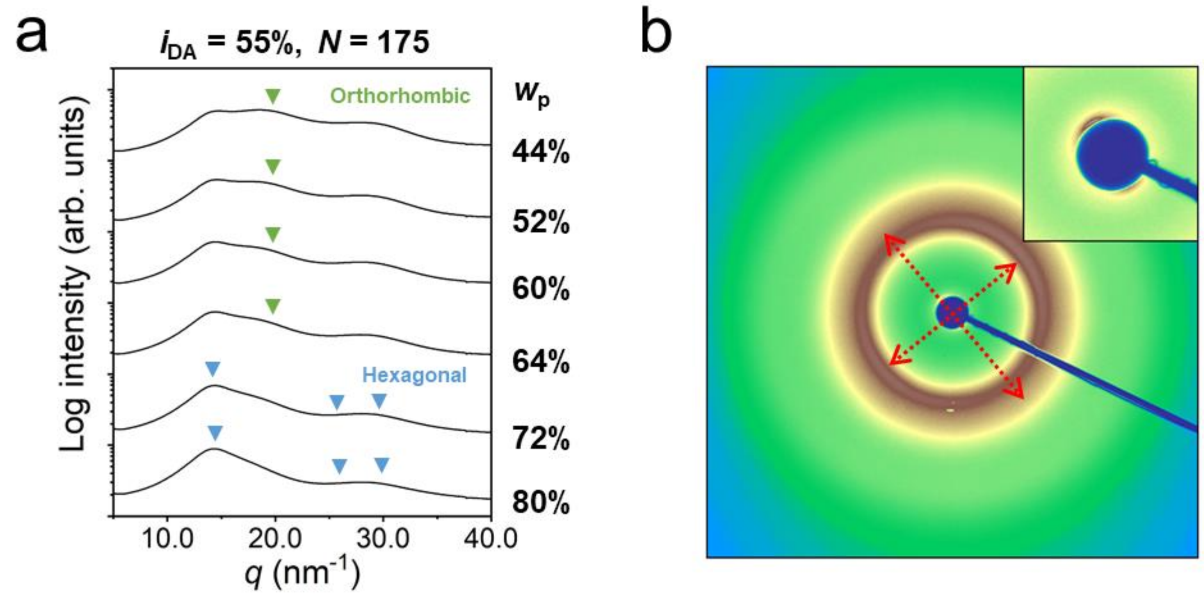

Supplementary Fig. 17. (a) 1D WAXS data of aqueous solutions of P(DA-*r*-PEGA) with i_{DA} = 55% and N = 175 with different concentrations at room temperature. Diffraction peaks for the orthorhombic and hexagonal alkyl chain packings are marked with green and blue triangles, respectively. (b) 2D WAXS pattern at 64 wt% concentration. An inset shows an enlarged image at the beam stop, showing the direction of the micellar lamellae.

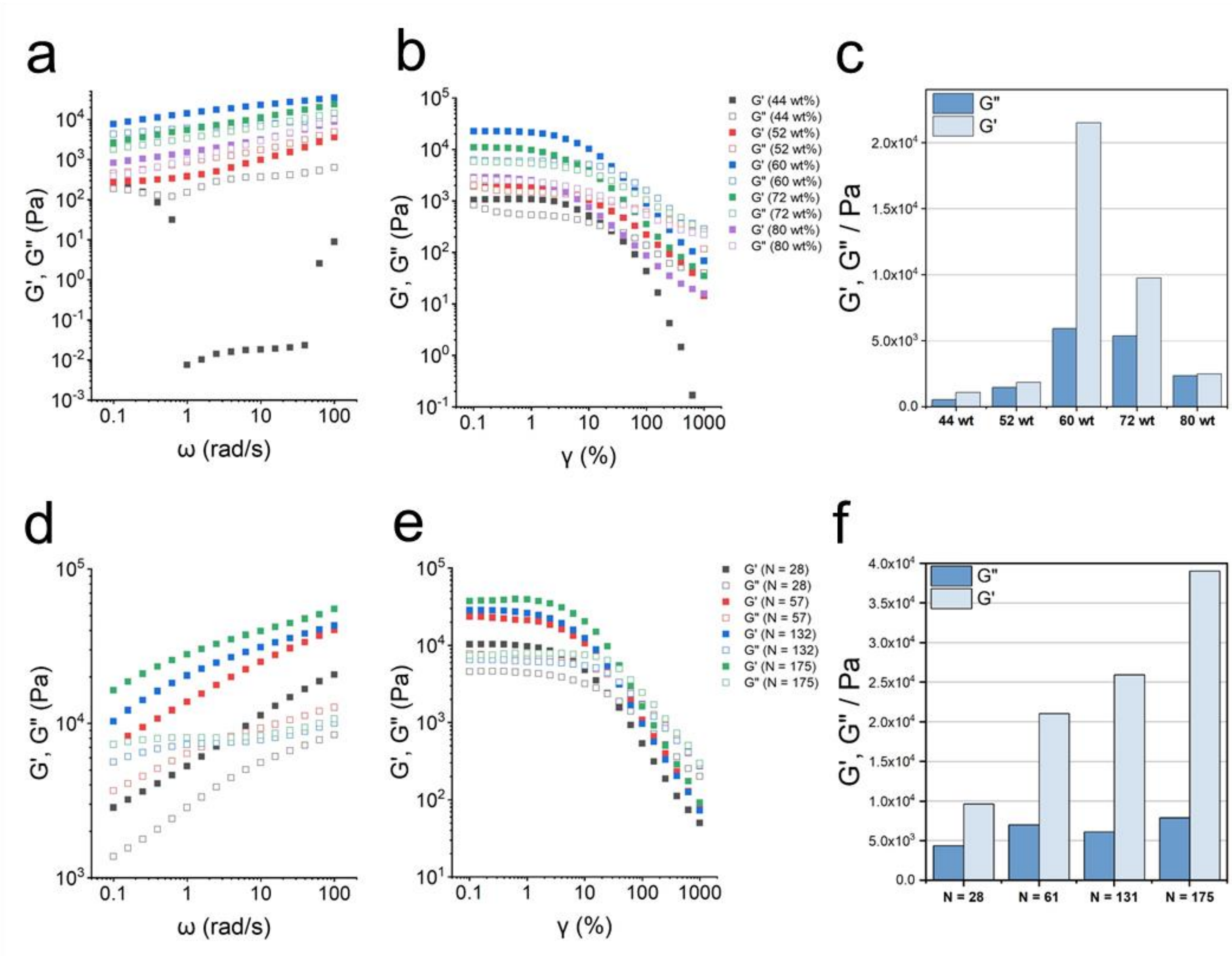

Supplementary Fig. 18. Dynamic oscillatory shear measurements of aqueous solutions of P(DA-*r*-PEGA)s with i_{DA} = 55%. The data were recorded at room temperature. (a-c) Variation in concentration. A polymer with N = 175 was used. (d-f) Variation in N. Concentration was fixed as 64 wt%.

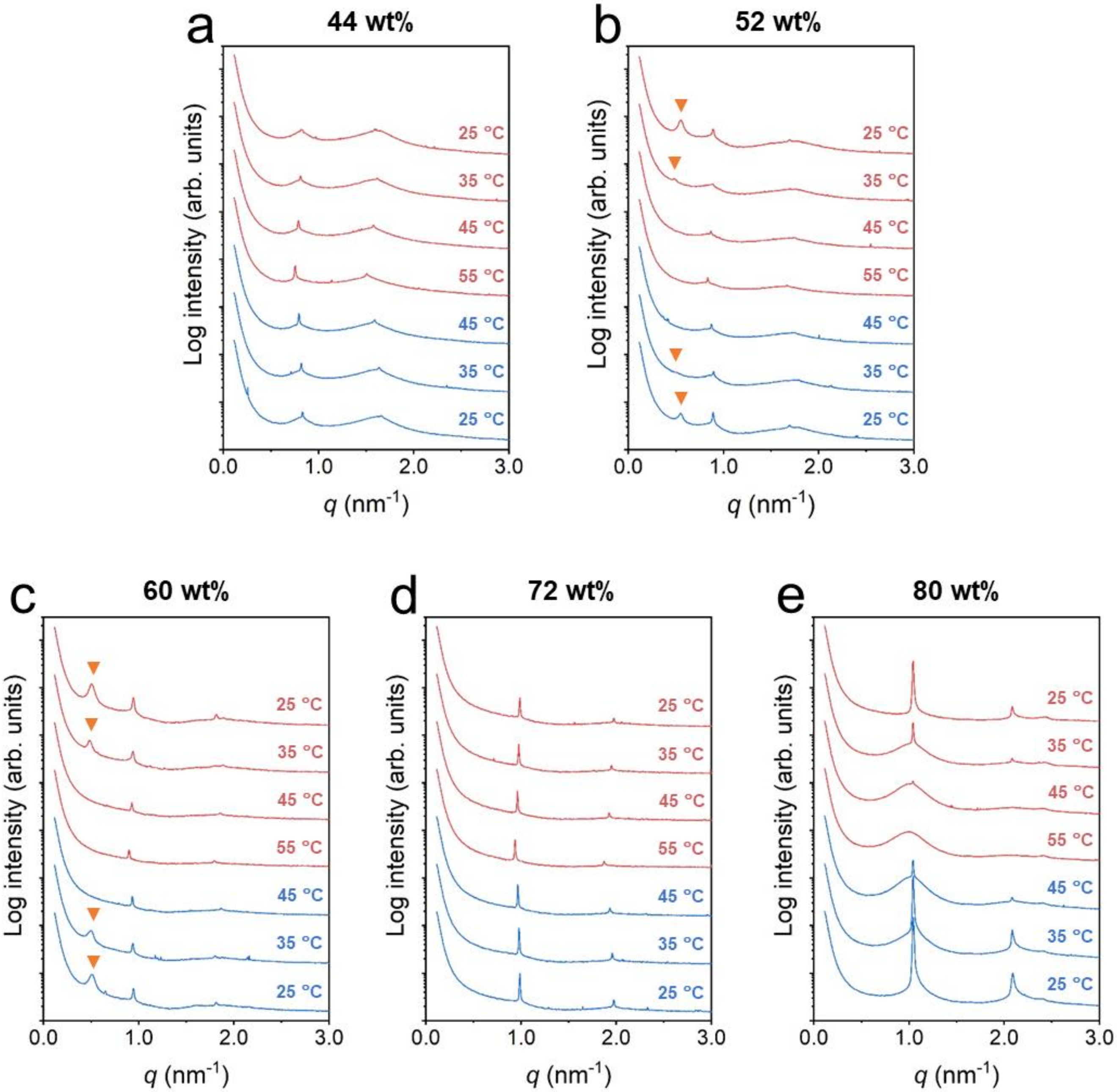

Supplementary Fig. 19. Temperature-dependent 1D SAXS data of aqueous solutions of P(DA-*r*-PEGA) with i_{DA} = 55% and N = 175. Scattering peaks related to the L_f phase are marked with the orange triangles.

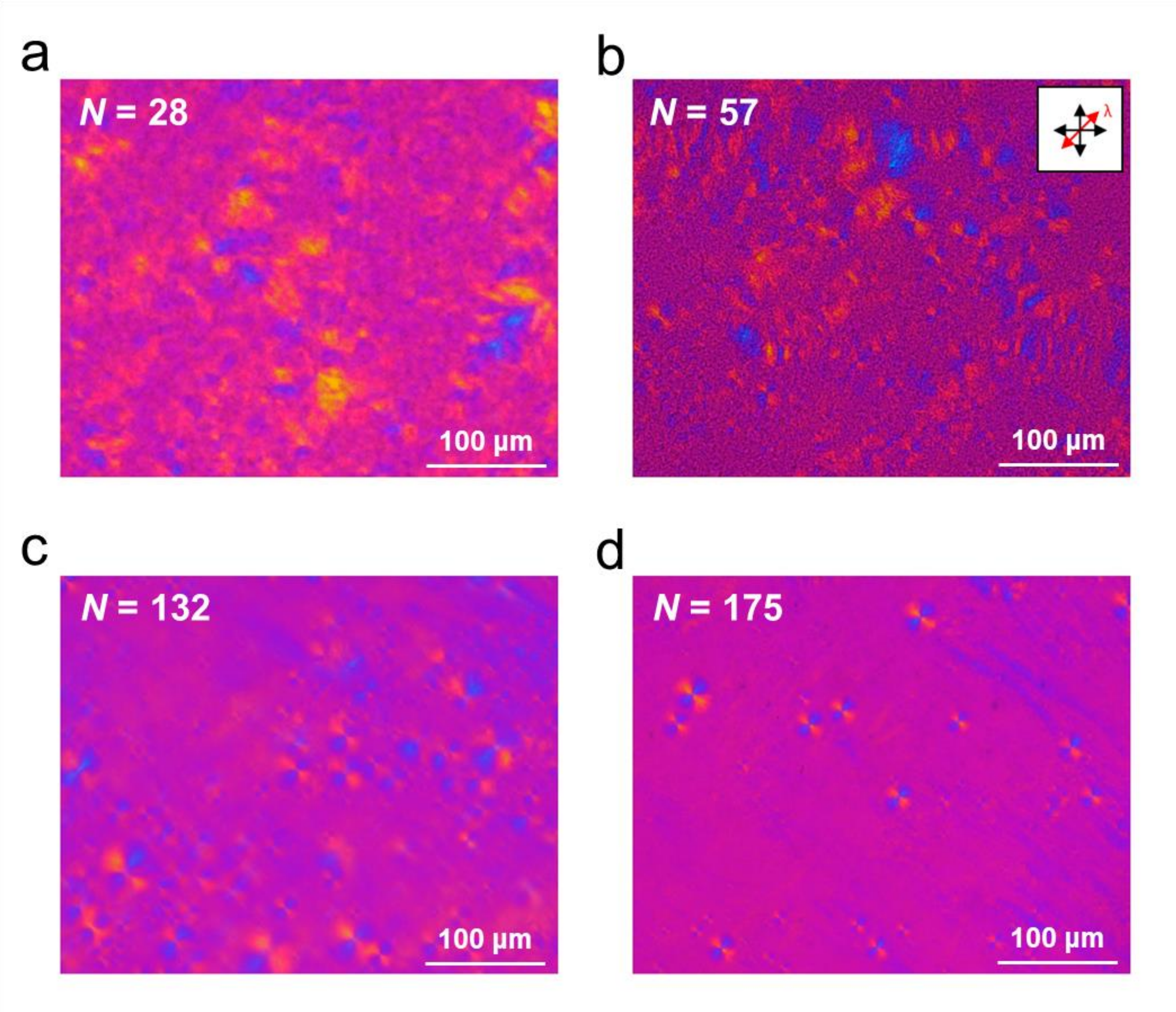

Supplementary Fig. 20. Polarized optical micrographs of aqueous solutions of P(DA-*r*-PEGA)s with i_{DA} = 55% and different *N*. The images were obtained upon slow cooling (0.5 °C/min) from the isotropic state at 65 °C. (a) *N* = 28. (b) *N* = 57. (c) *N* = 132. (d) *N* = 175. Spherulites appear as Maltese cross patterns with blue and bright purple colors.

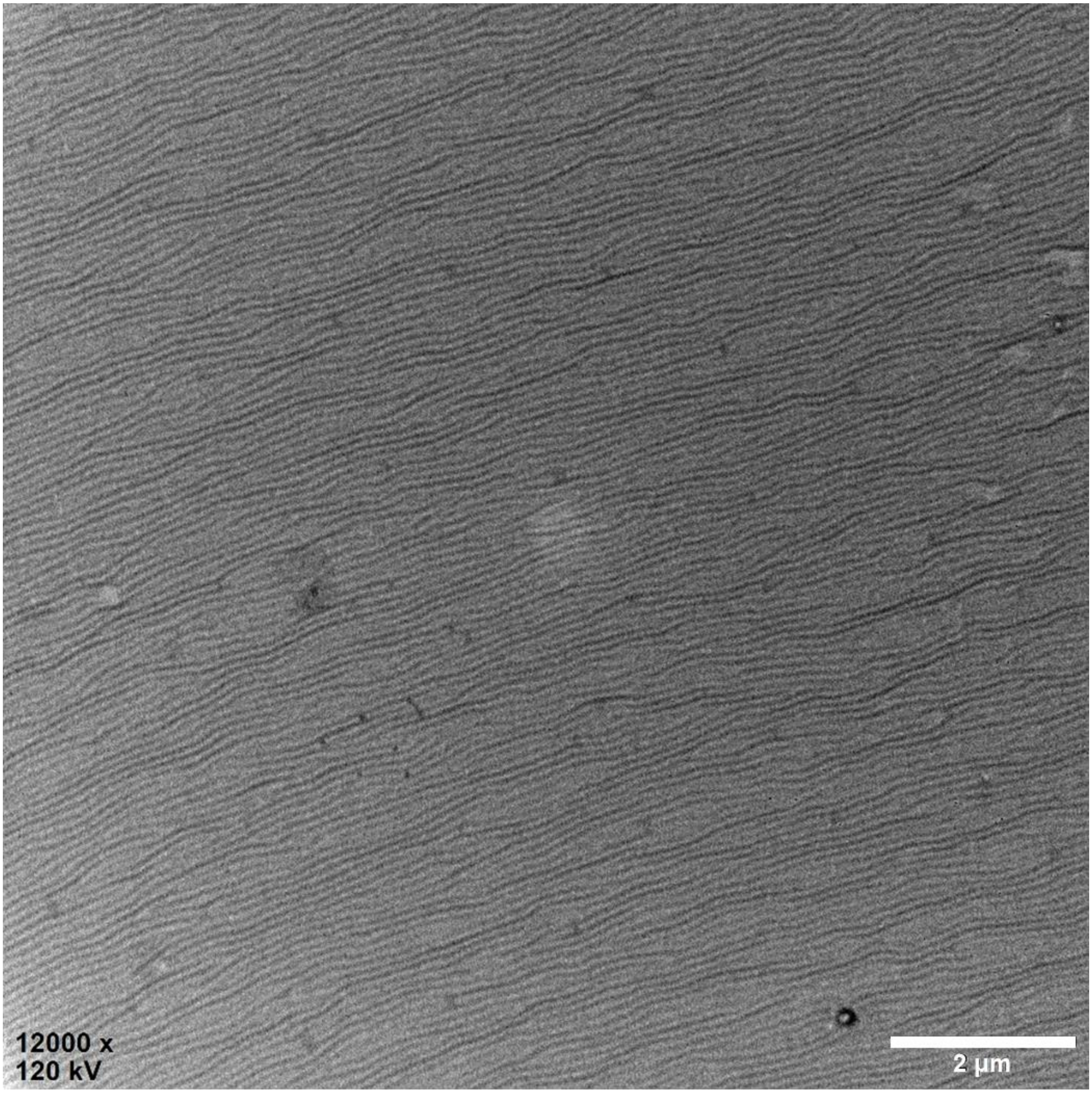

Supplementary Fig. 21. Low-magnification TEM image of the vitrified solution of P(DA_{73}-*r*-$PEGA_{59}$) (i_{DA} = 55%, N = 132) at 60 wt% concentration. Fig. 2f is a part of this image. The solution contained 5 wt% of sodium silicate and 0.2 M HCl. The microtomed film was further stained with RuO_4.

S2.2. Aqueous solutions of P(DA-*r*-PEGA)s with i_{DA} = 60% and 50%

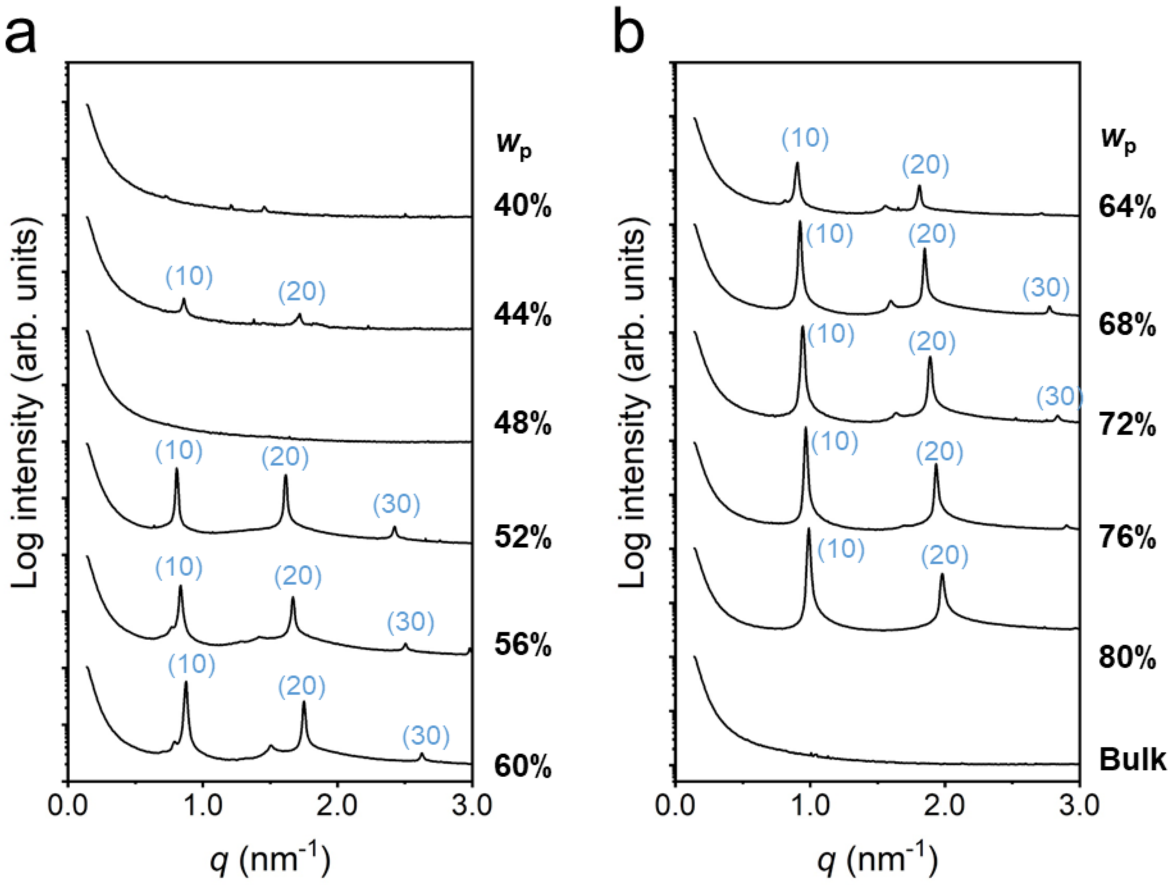

Supplementary Fig. 22. 1D SAXS data of P(DA$_{21}$-*r*-PEGA$_{14}$) (i_{DA} = 60%, *N* = 35) aqueous solutions at different concentrations. (a) 40 to 60 wt%. (b) 64 to 100 wt% (bulk). Miller indices for the following unit cell structure are assigned to the selected peaks in the plot: L$_a$ (fluidic multilamellar, blue).

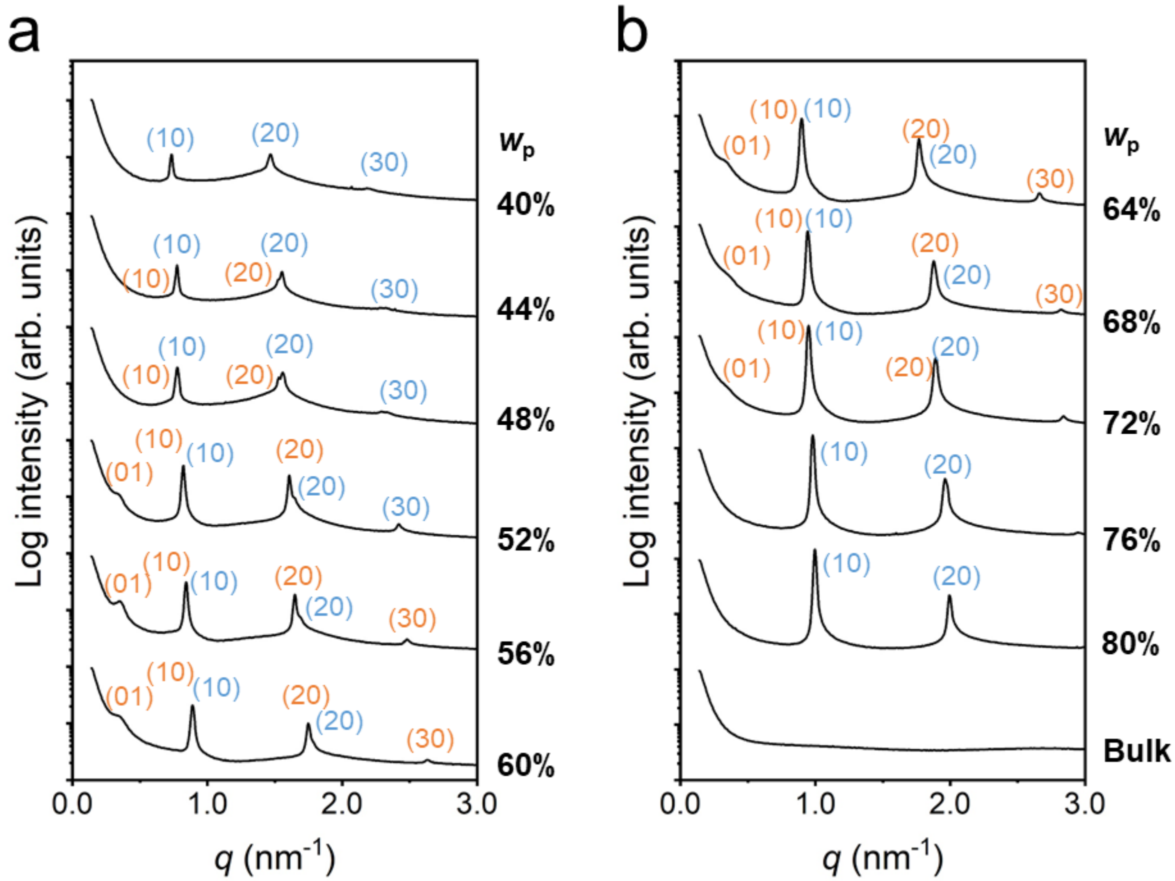

Supplementary Fig. 23. 1D SAXS data of P(DA$_{41}$-*r*-PEGA$_{27}$) (i_{DA} = 60%, N = 68) aqueous solutions at different concentrations. (a) 40 to 60 wt%. (b) 64 to 100 wt% (bulk). Miller indices for the following unit cell structure are assigned to the selected peaks in the plot: L$_a$ (fluidic multilamellar, blue); L$_f$ (bilayer-folded lamellar, orange).

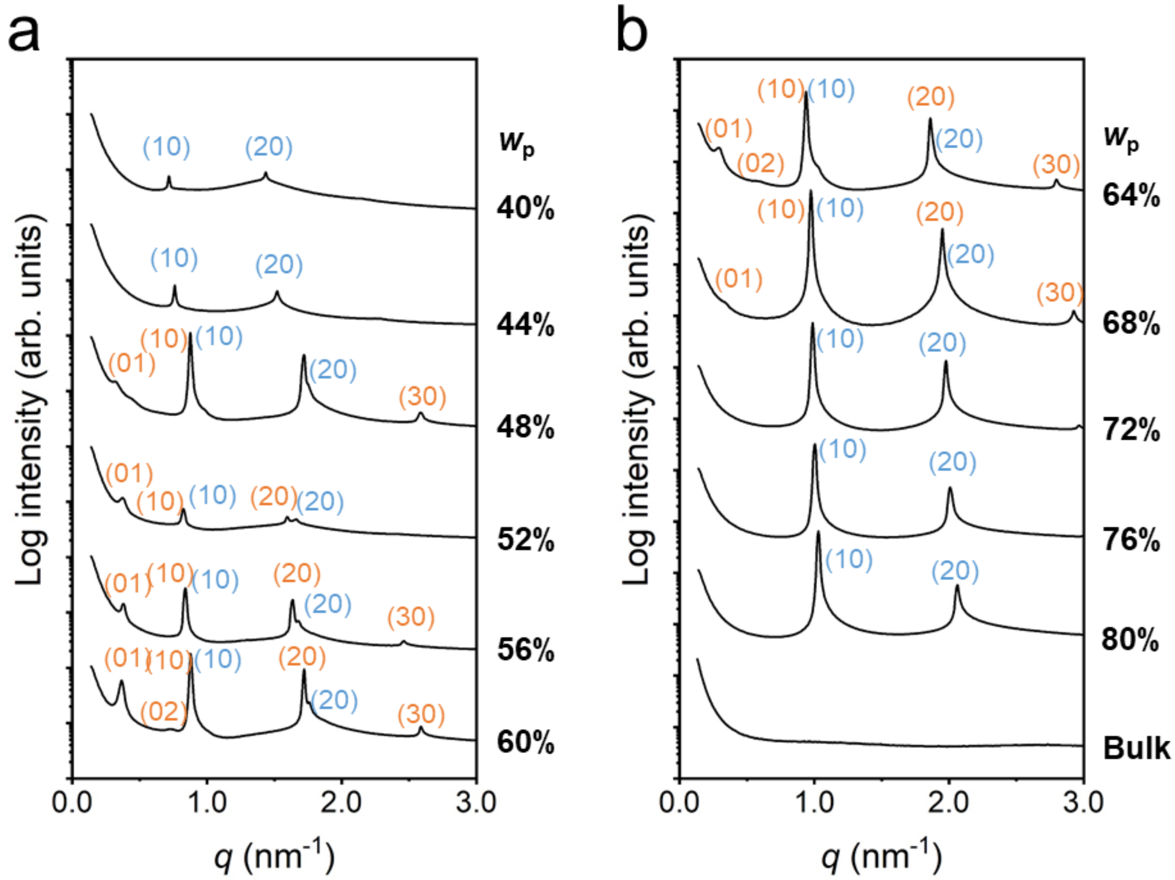

Supplementary Fig. 24. 1D SAXS data of P(DA_{73}-*r*-$PEGA_{48}$) (i_{DA} = 60%, N = 121) aqueous solutions at different concentrations. (a) 40 to 60 wt%. (b) 64 to 100 wt% (bulk). Miller indices for the following unit cell structure are assigned to the selected peaks in the plot: L_a (fluidic multilamellar, blue); L_f (bilayer-folded lamellar, orange).

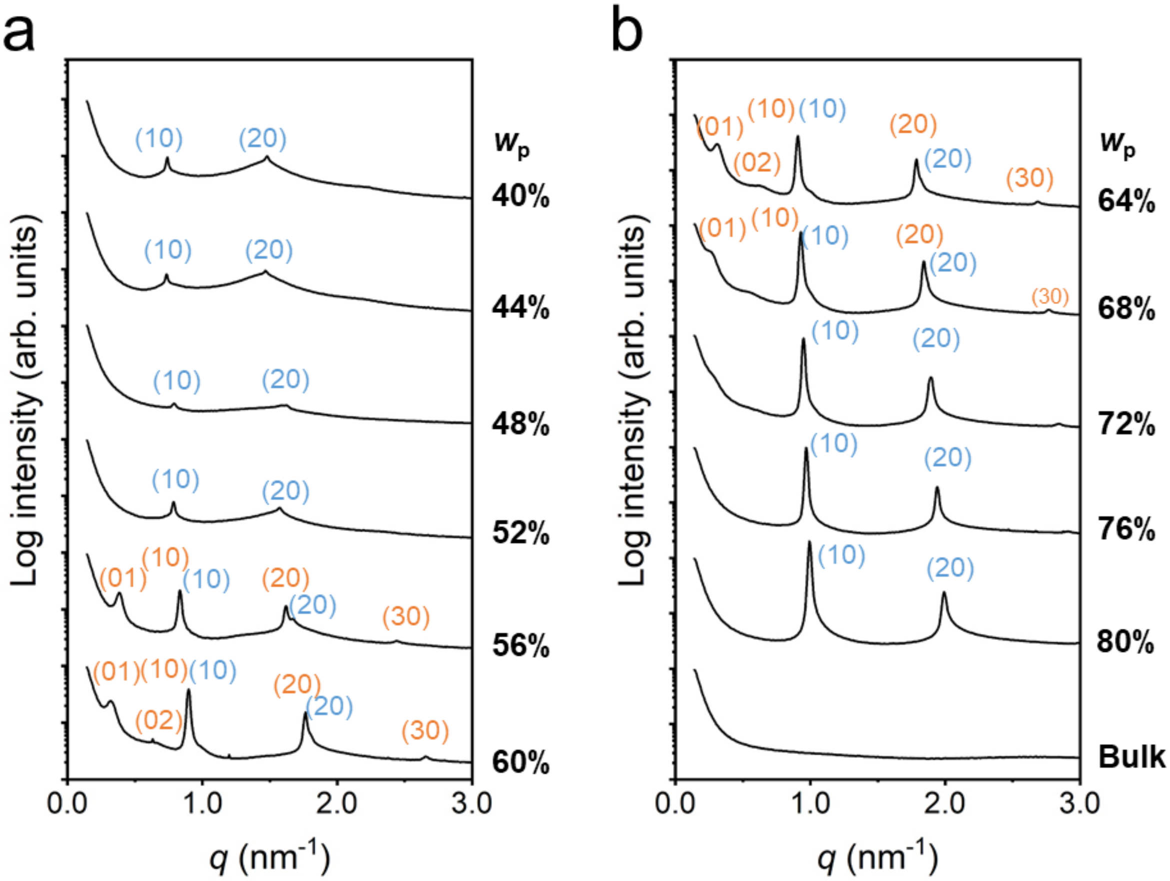

Supplementary Fig. 25. 1D SAXS data of P(DA_{115}-*r*-$PEGA_{76}$) (i_{DA} = 60%, N = 191) aqueous solutions at different concentrations. (a) 40 to 60 wt%. (b) 64 to 100 wt% (bulk). Miller indices for the following unit cell structure are assigned to the selected peaks in the plot: L_a (fluidic multilamellar, blue); L_f (bilayer-folded lamellar, orange).

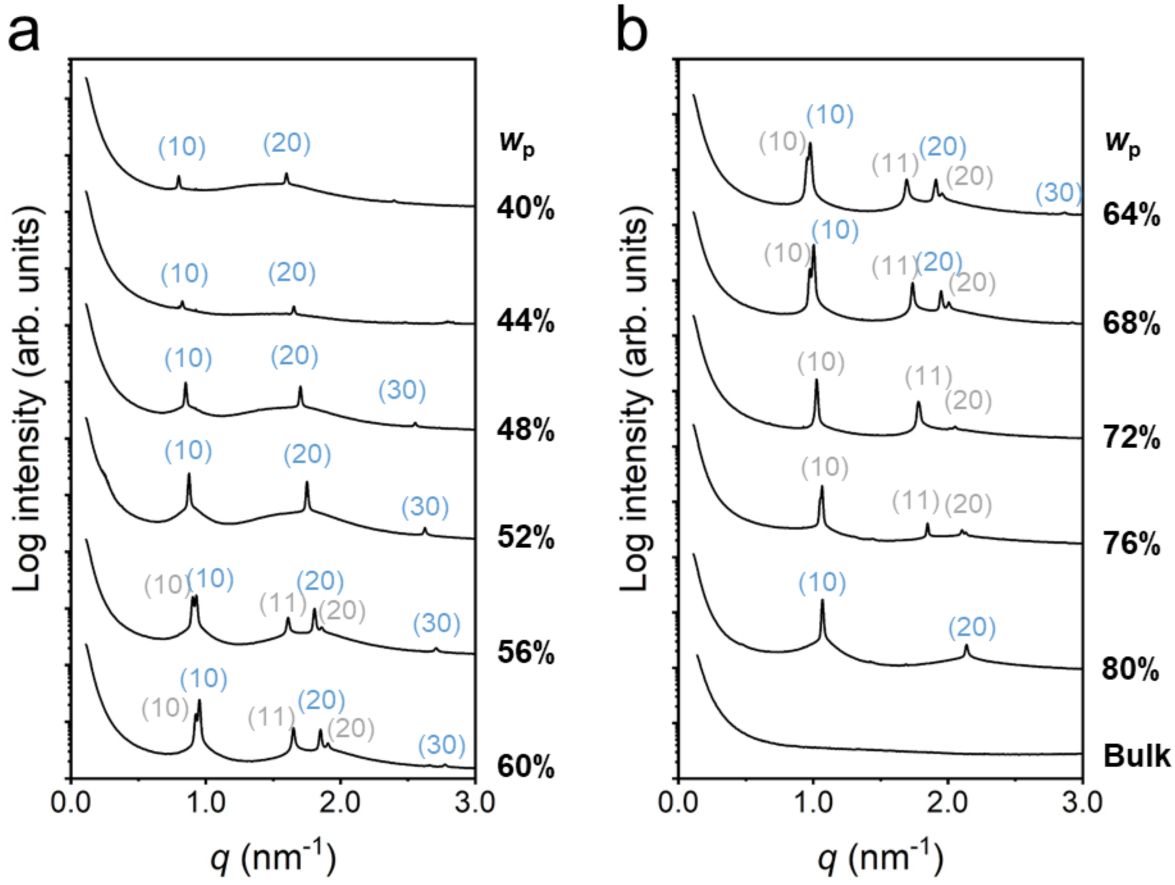

Supplementary Fig. 26. 1D SAXS data of P(DA_{18}-*r*-$PEGA_{18}$) (i_{DA} = 50%, N = 36) aqueous solutions at different concentrations. (a) 40 to 60 wt%. (b) 64 to 100 wt% (bulk). Miller indices for the following unit cell structure are assigned to the selected peaks in the plot: L_a (fluidic multilamellar, blue); H_I (hexagonal, grey).

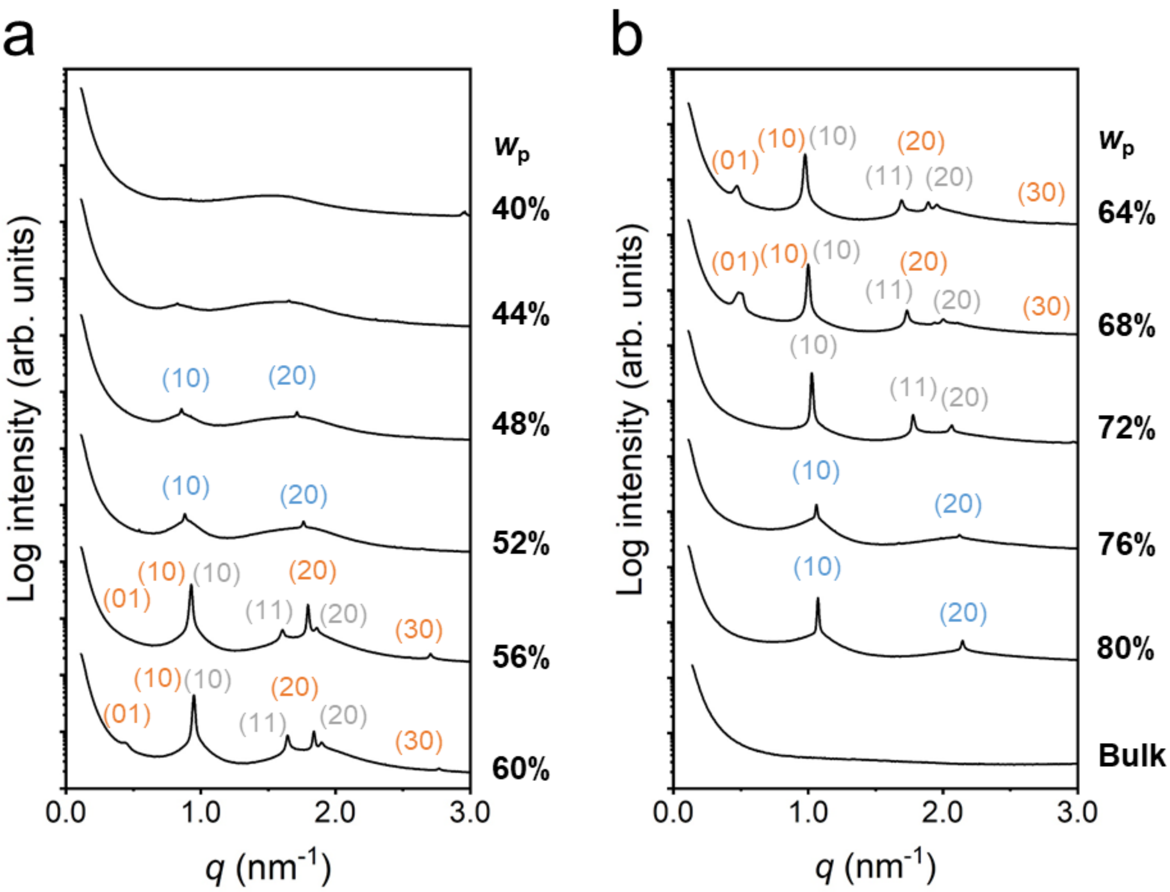

Supplementary Fig. 27. 1D SAXS data of P(DA$_{33}$-*r*-PEGA$_{33}$) (i_{DA} = 50%, *N* = 66) aqueous solutions at different concentrations. (a) 40 to 60 wt%. (b) 64 to 100 wt% (bulk). Miller indices for the following unit cell structure are assigned to the selected peaks in the plot: L_a (fluidic multilamellar, blue); H_I (hexagonal, grey); L_f (bilayer-folded lamellar, orange).

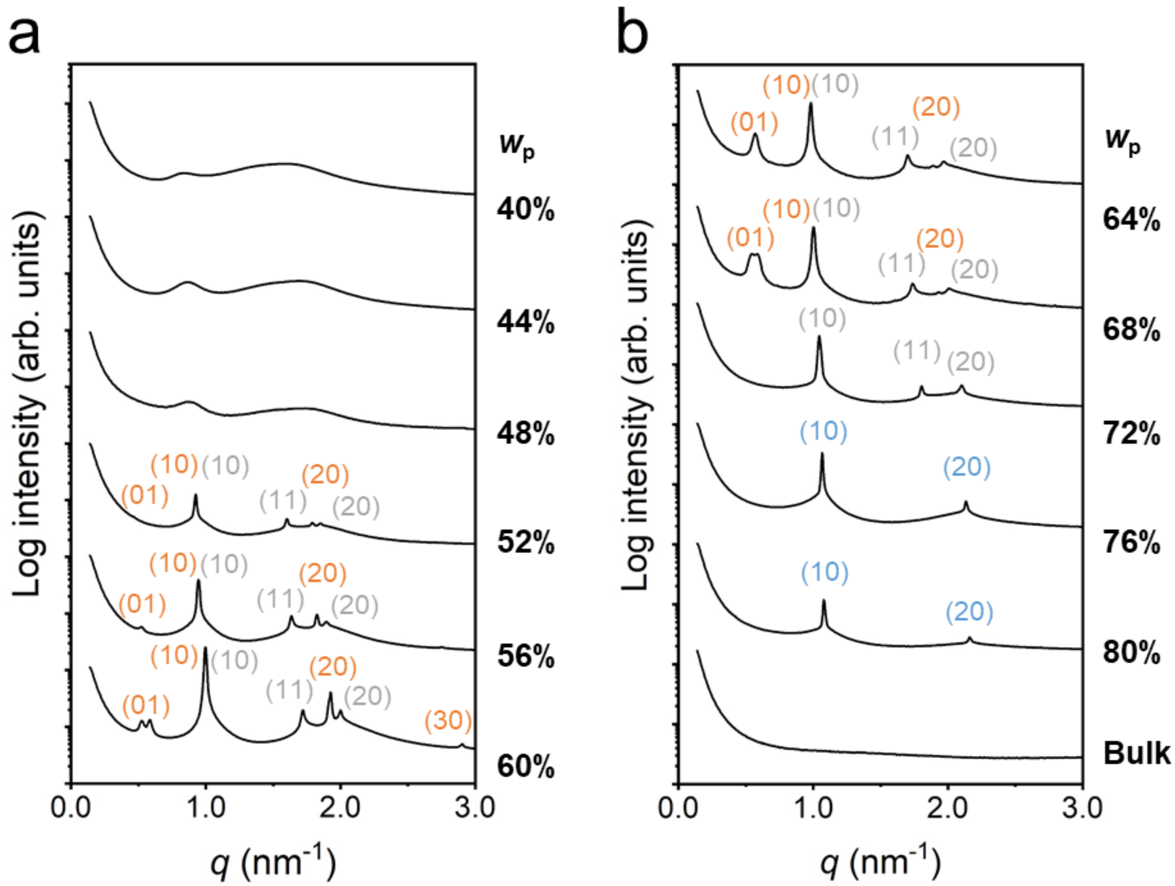

Supplementary Fig. 28. 1D SAXS data of P(DA$_{62}$-*r*-PEGA$_{62}$) (i_{DA} = 50%, N = 124) aqueous solutions at different concentrations. (a) 40 to 60 wt%. (b) 64 to 100 wt% (bulk). Miller indices for the following unit cell structure are assigned to the selected peaks in the plot: L$_a$ (fluidic multilamellar, blue); H$_I$ (hexagonal, grey); L$_f$ (bilayer-folded lamellar, orange).

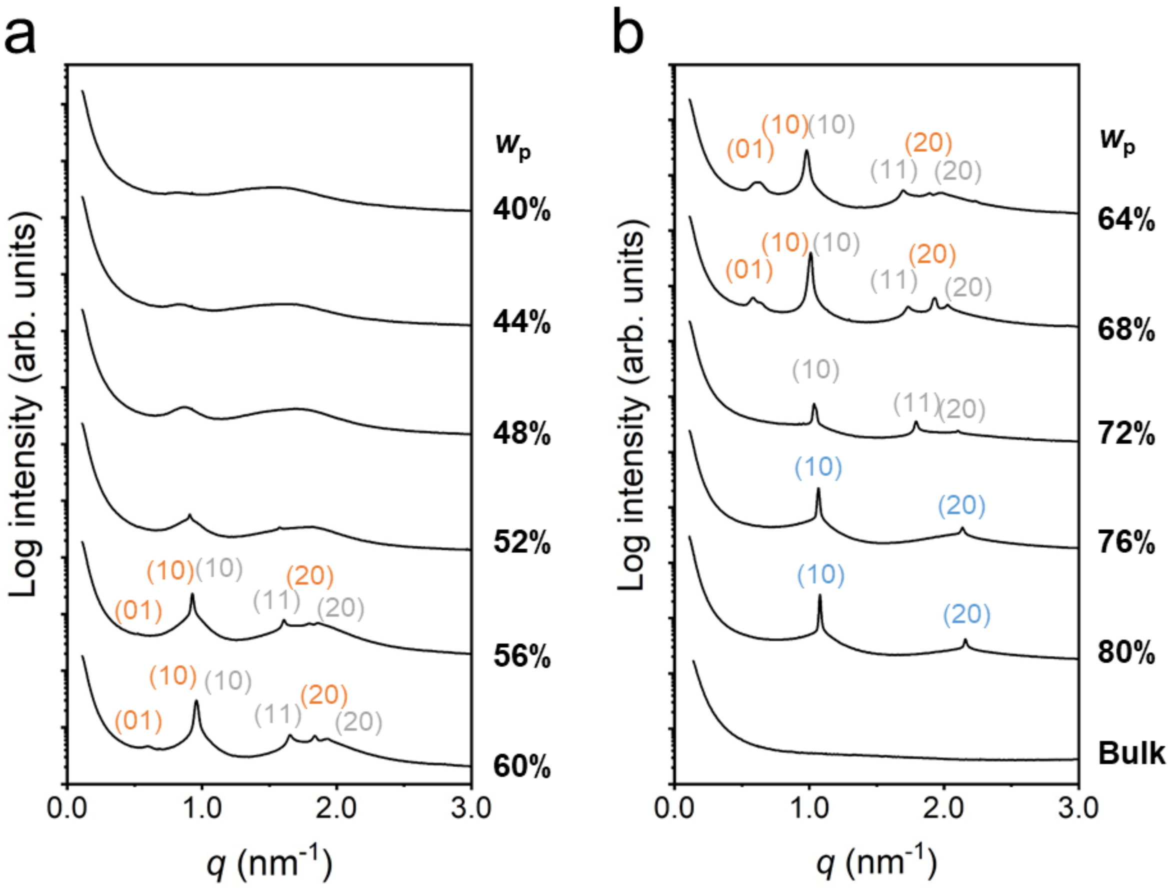

Supplementary Fig. 29. 1D SAXS data of P(DA$_{99}$-*r*-PEGA$_{99}$) (i_{DA} = 50%, *N* = 198) aqueous solutions at different concentrations. (a) 40 to 60 wt%. (b) 64 to 100 wt% (bulk). Miller indices for the following unit cell structure are assigned to the selected peaks in the plot: L_a (fluidic multilamellar, blue); H_I (hexagonal, grey); L_f (bilayer-folded lamellar, orange).

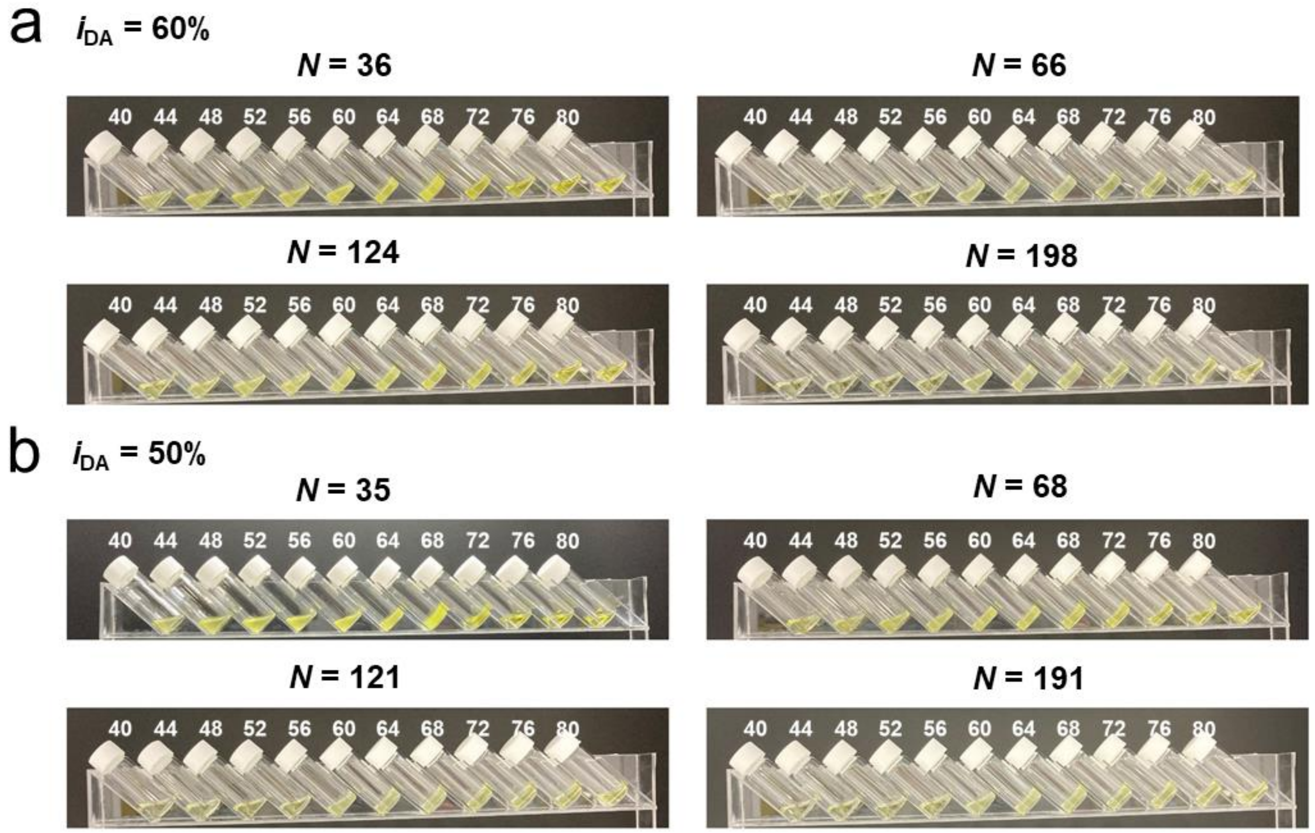

Supplementary Fig. 30. Photos of P(DA-*r*-PEGA) aqueous solutions at room temperature. (a) i_{DA} = 60%. (b) i_{DA} = 50%. The concentration range for the gelation determined by the inverted vial method was 56 to 72 wt%.

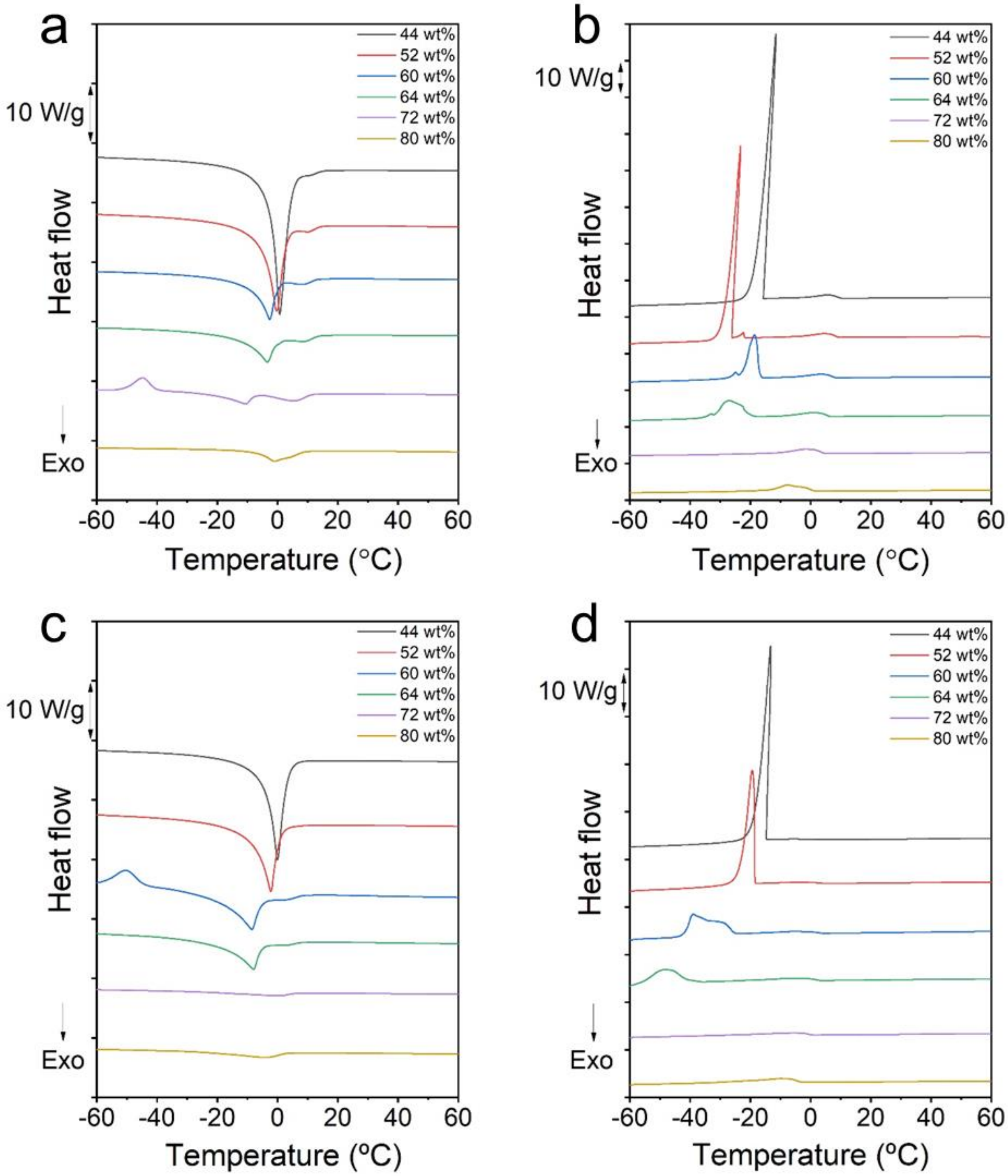

Supplementary Fig. 31. DSC thermograms P(DA-*r*-PEGA) aqueous solutions during the second cycle of heating (left)/cooling (right). (a-b) i_{DA} = 60%, N = 191. (c-d) i_{DA} = 50%, N = 198. In the cooling scan, the strong exothermic transition below -10 °C was assigned to the crystallization of the PEGA side chains swollen with water. The relatively weak transition appearing around 0 °C was attributed to the solidification of the DA side chains.

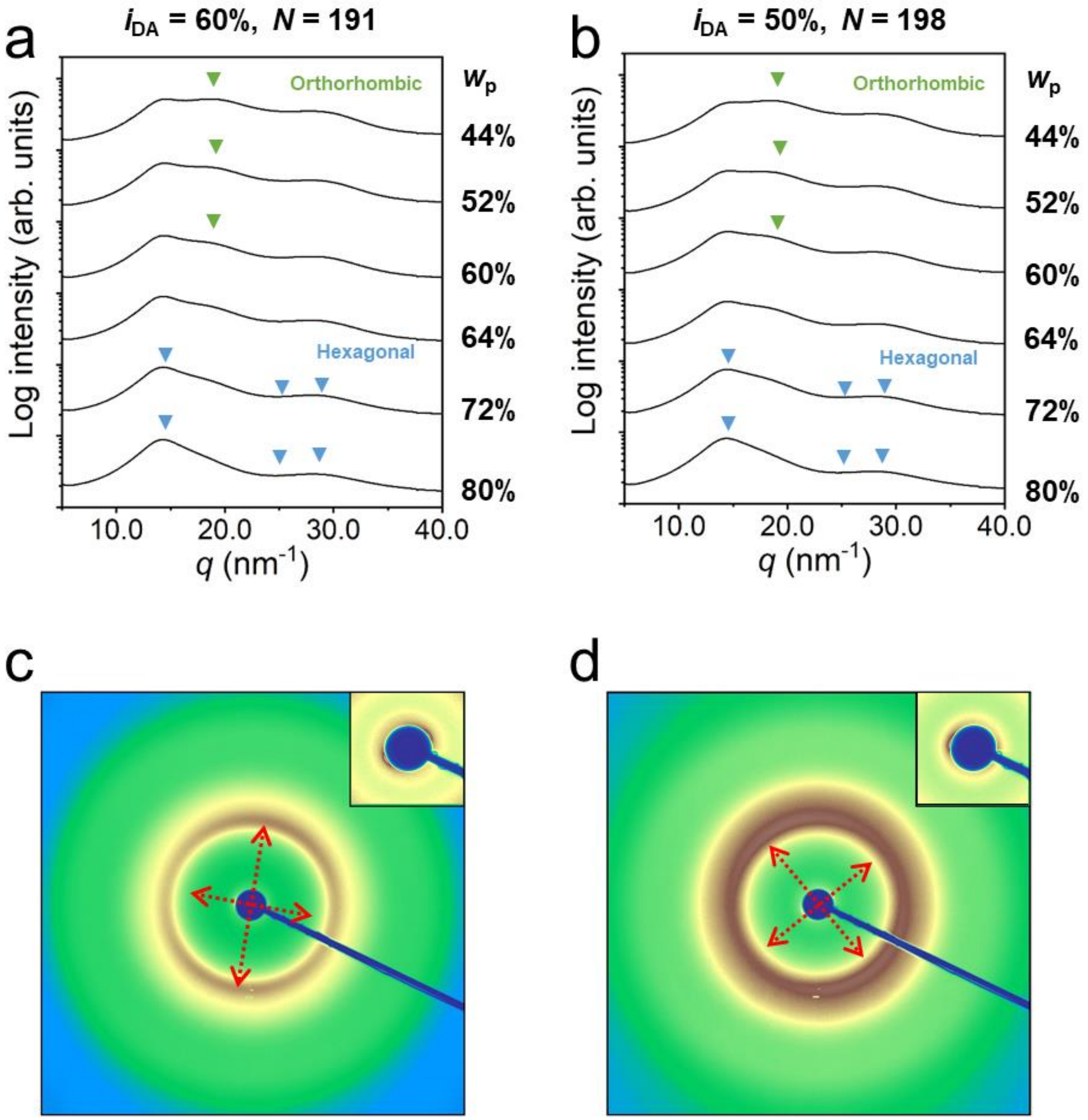

Supplementary Fig. 32. (a-b) 1D WAXS data of P(DA-*r*-PEGA) aqueous solutions with different concentrations at room temperature. (a) i_{DA} = 60%, N = 191. (b) i_{DA} = 50%, N = 198. Diffraction peaks for the orthorhombic and hexagonal alkyl chain packings are marked with green and blue triangles, respectively. (c-d) 2D WAXS patterns of P(DA-*r*-PEGA) solutions at 64 wt% concentration. An inset shows an enlarged image at the beam stop, showing direction of the micellar lamellae. (c) i_{DA} = 60%, N = 191. (d) i_{DA} = 50%, N = 198.

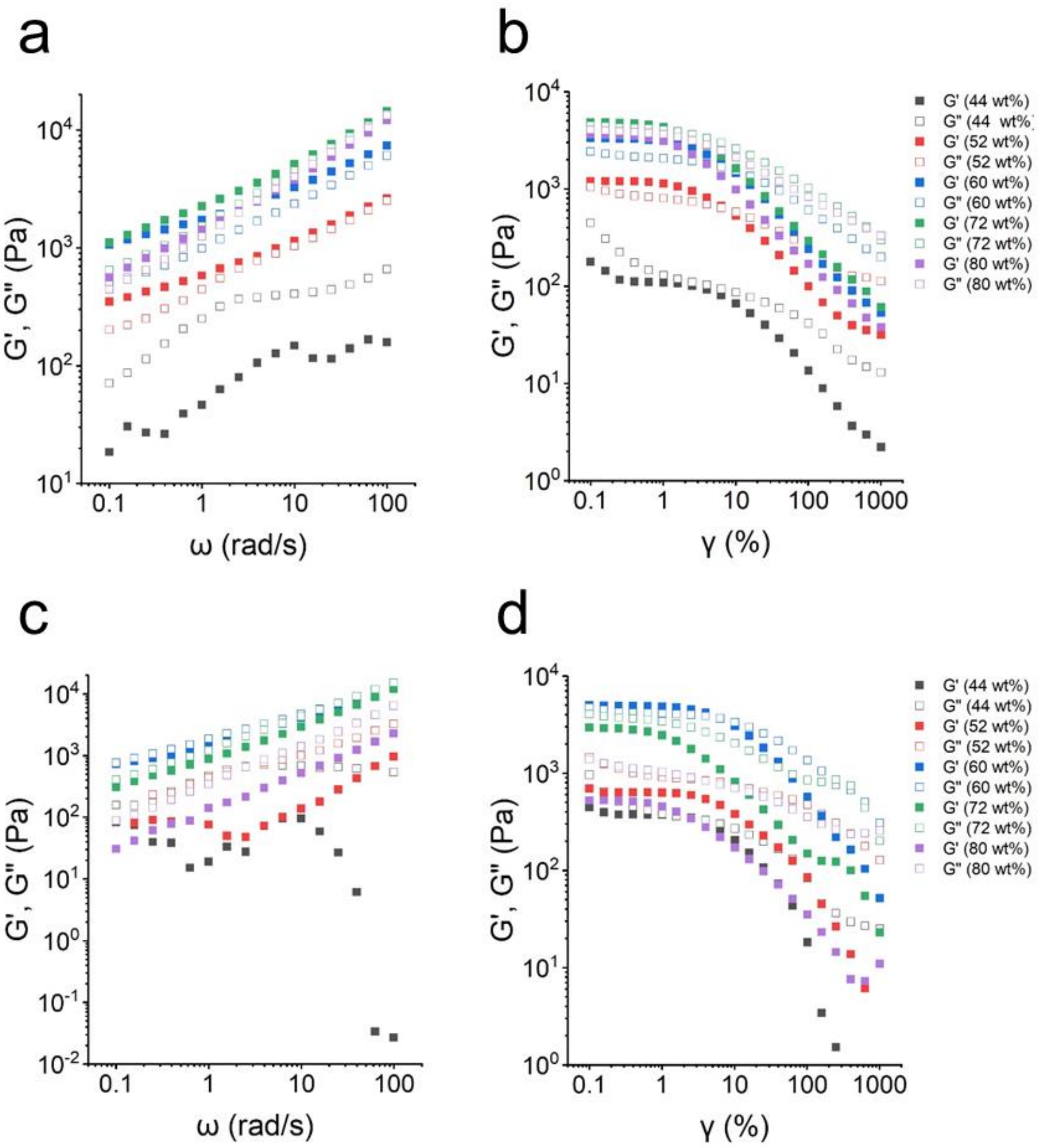

Supplementary Fig. 33. Dynamic oscillatory shear measurements of aqueous solutions of P(DA-*r*-PEGA)s with i_{DA} = 60%, N = 191 (a-b) and 50% (c-d), N = 198. The data were recorded at room temperature.

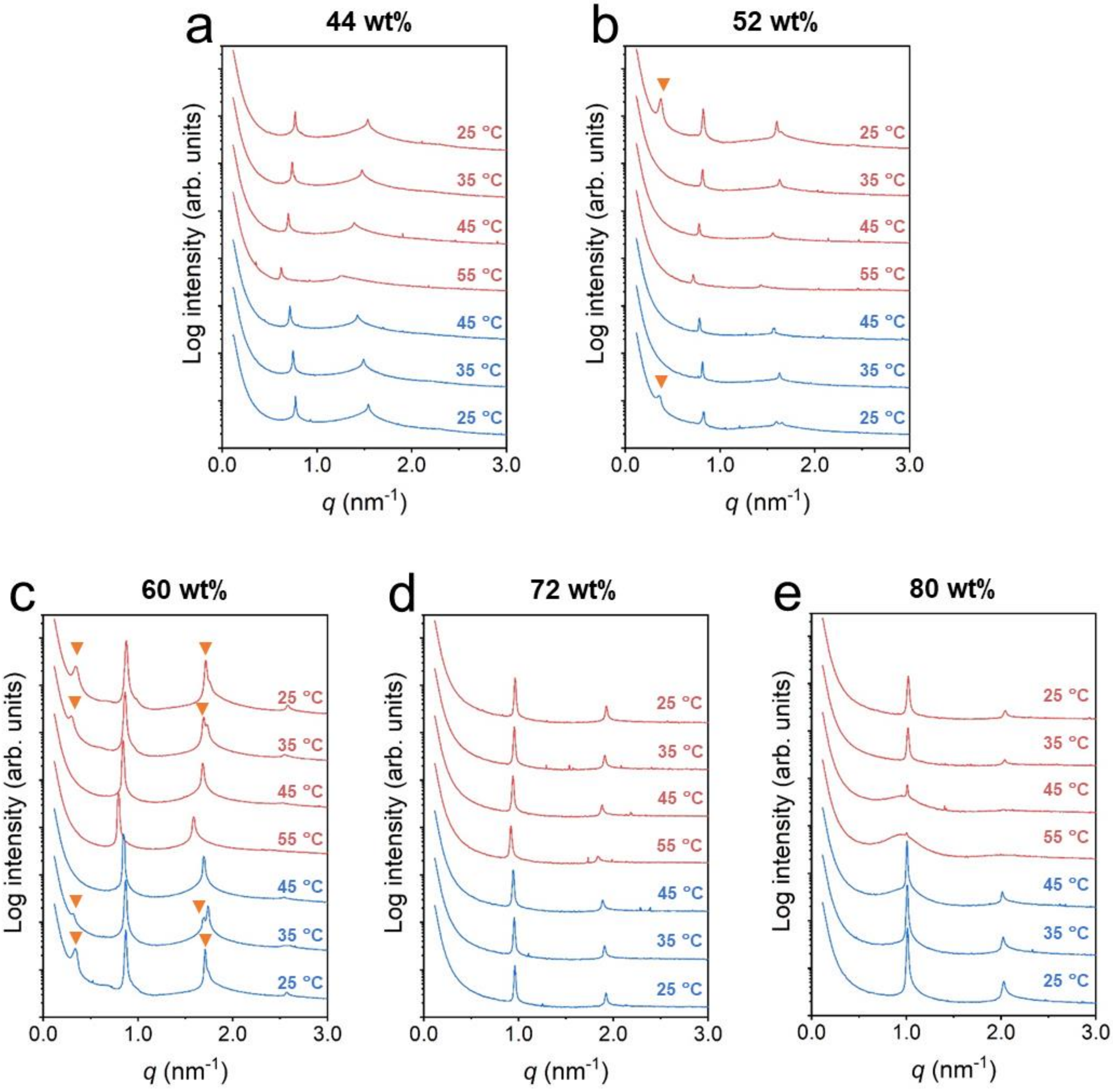

Supplementary Fig. 34. Temperature-dependent 1D SAXS data of aqueous solutions of P(DA-*r*-PEGA) with i_{DA} = 60%, N = 191. Scattering peaks related to the L_f phase are marked with orange triangles.

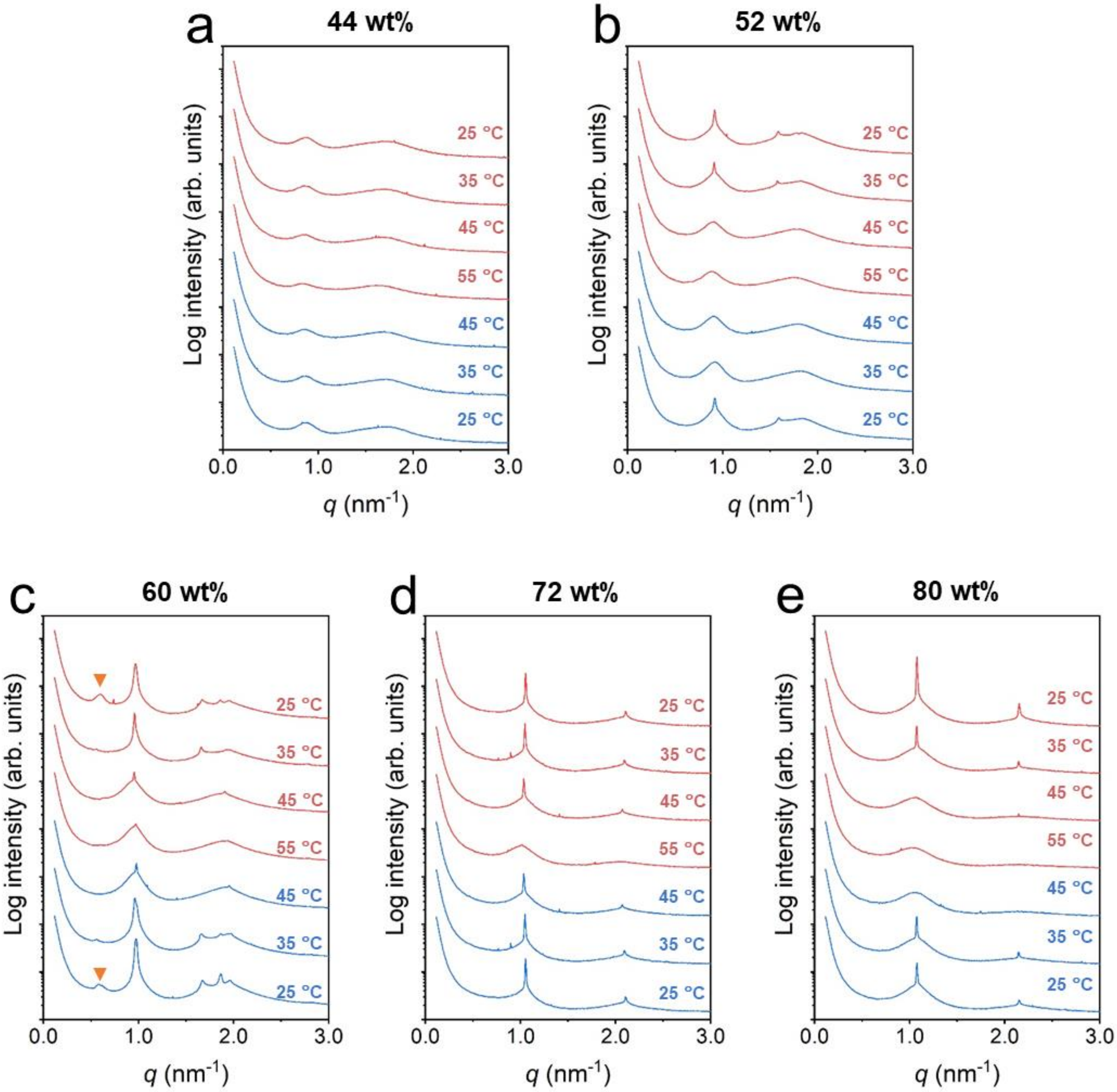

Supplementary Fig. 35. Temperature-dependent 1D SAXS data of aqueous solutions of P(DA-*r*-PEGA) with i_{DA} = 50%, N = 198. Scattering peaks related to the L_f phase are marked with orange triangles.

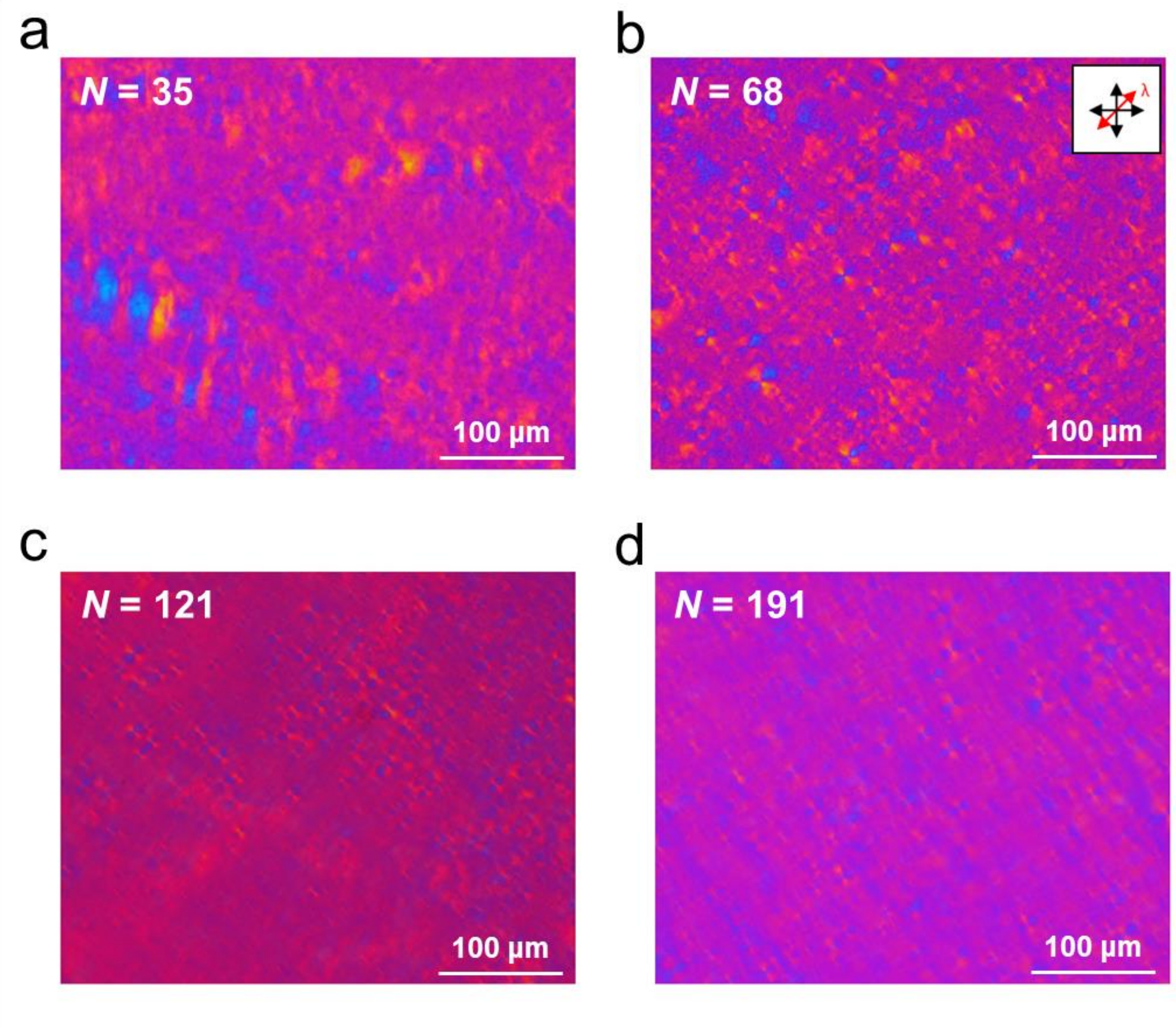

Supplementary Fig. 36. Polarized optical micrographs of aqueous solutions of P(DA-*r*-PEGA)s with i_{DA} = 60% and different N. The images were obtained upon slow cooling (0.5 °C/min) from the isotropic state at 65 °C. (a) N = 35. (b) N = 68. (c) N = 121. (d) N = 191. Spherulites appear as Maltese cross patterns with blue and bright purple colors.

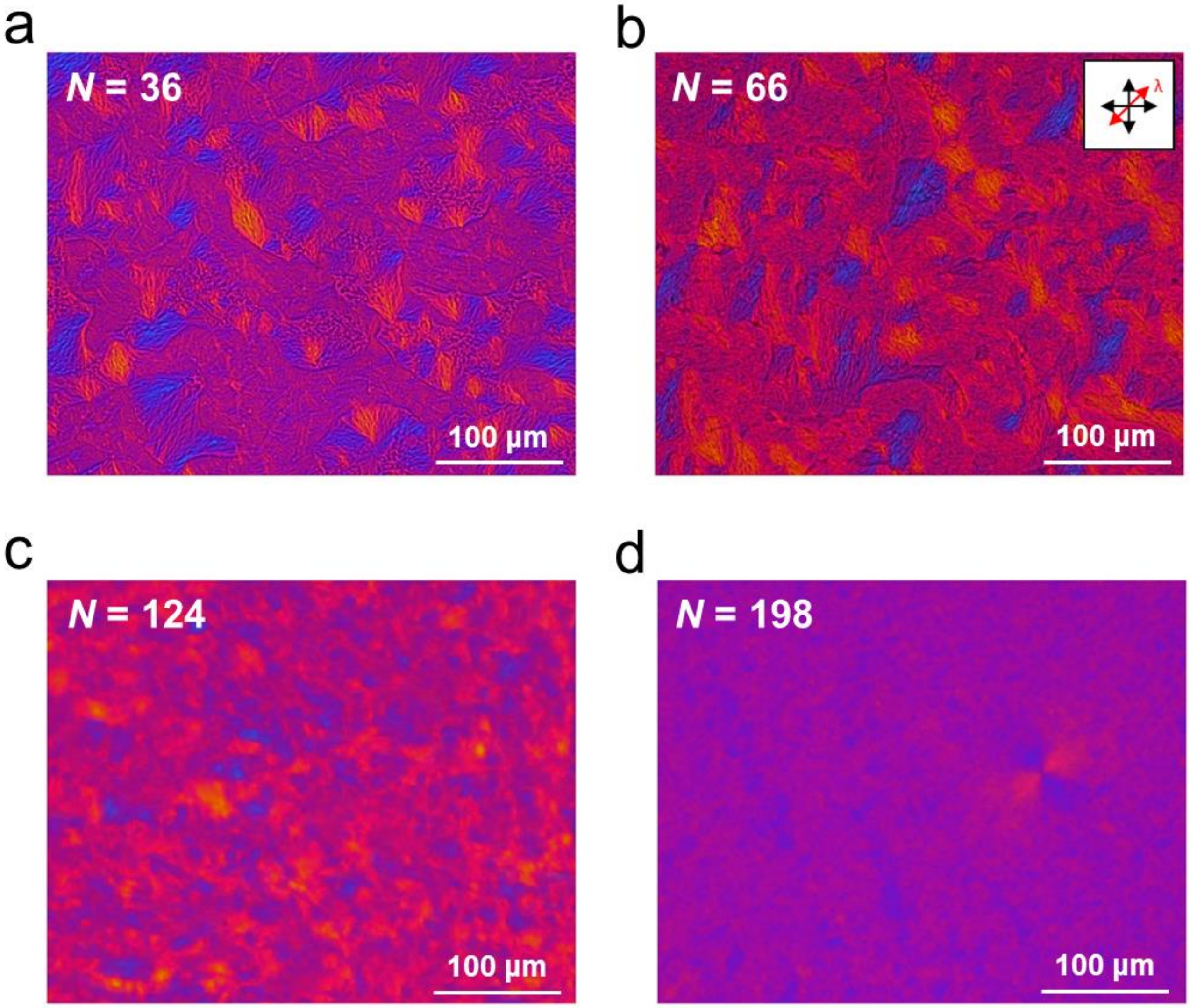

Supplementary Fig. 37. Polarized optical micrographs of aqueous solutions of P(DA-*r*-PEGA)s with i_{DA} = 60% and different N. The images were obtained upon slow cooling (0.5 °C/min) from the isotropic state at 55 °C. (a) N = 36. (b) N = 66. (c) N = 124. (d) N = 198. Spherulites appear as Maltese cross patterns with blue and bright purple colors.

S2.3. Oscillatory shear-aligned 2D SAXS data and additional TEM/SAXS analysis

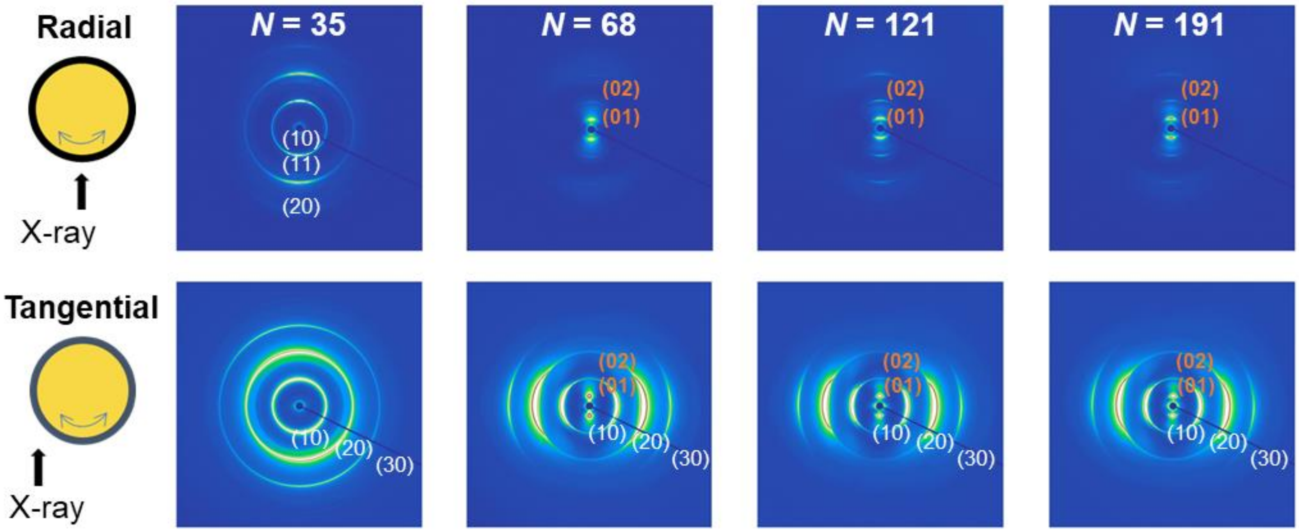

Supplementary Fig. S38. Oscillatory shear-aligned 2D SAXS pattern of P(DA-*r*-PEGA) solutions with i_{DA} = 60%.

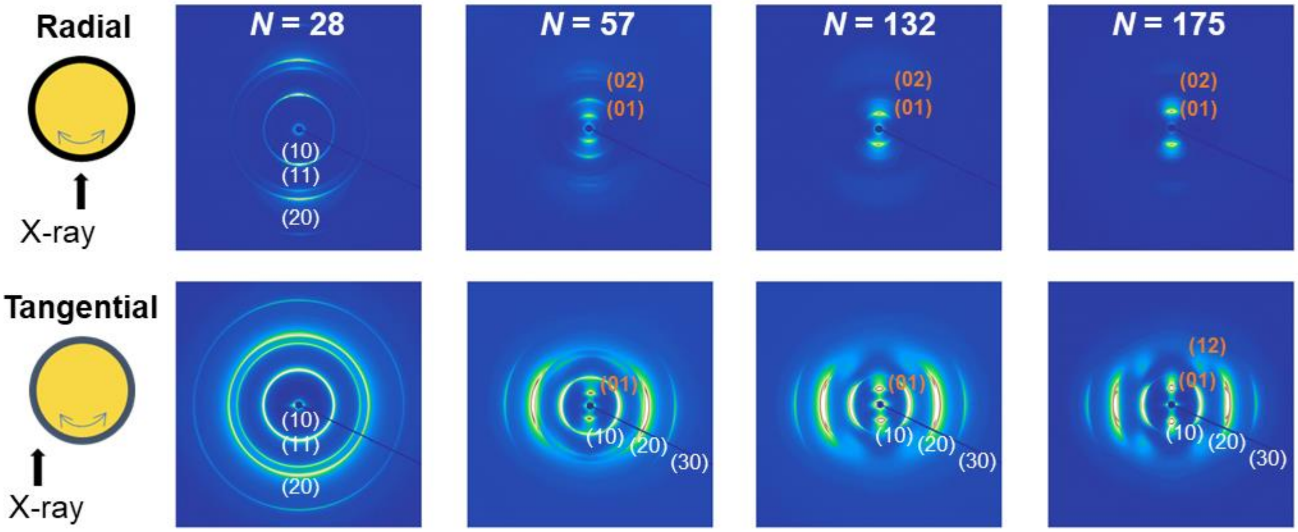

Supplementary Fig. 39. Oscillatory shear-aligned 2D SAXS pattern of P(DA-*r*-PEGA) solutions with i_{DA} = 55%.

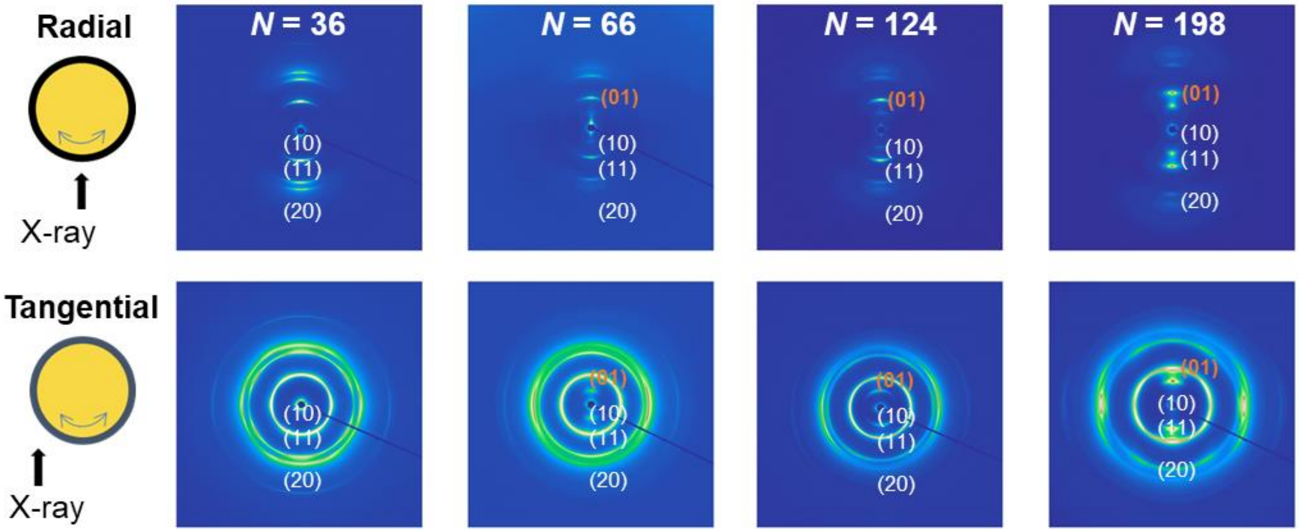

Supplementary Fig. 40. Oscillatory shear-aligned 2D SAXS pattern of P(DA-*r*-PEGA) solutions with i_{DA} = 50%.

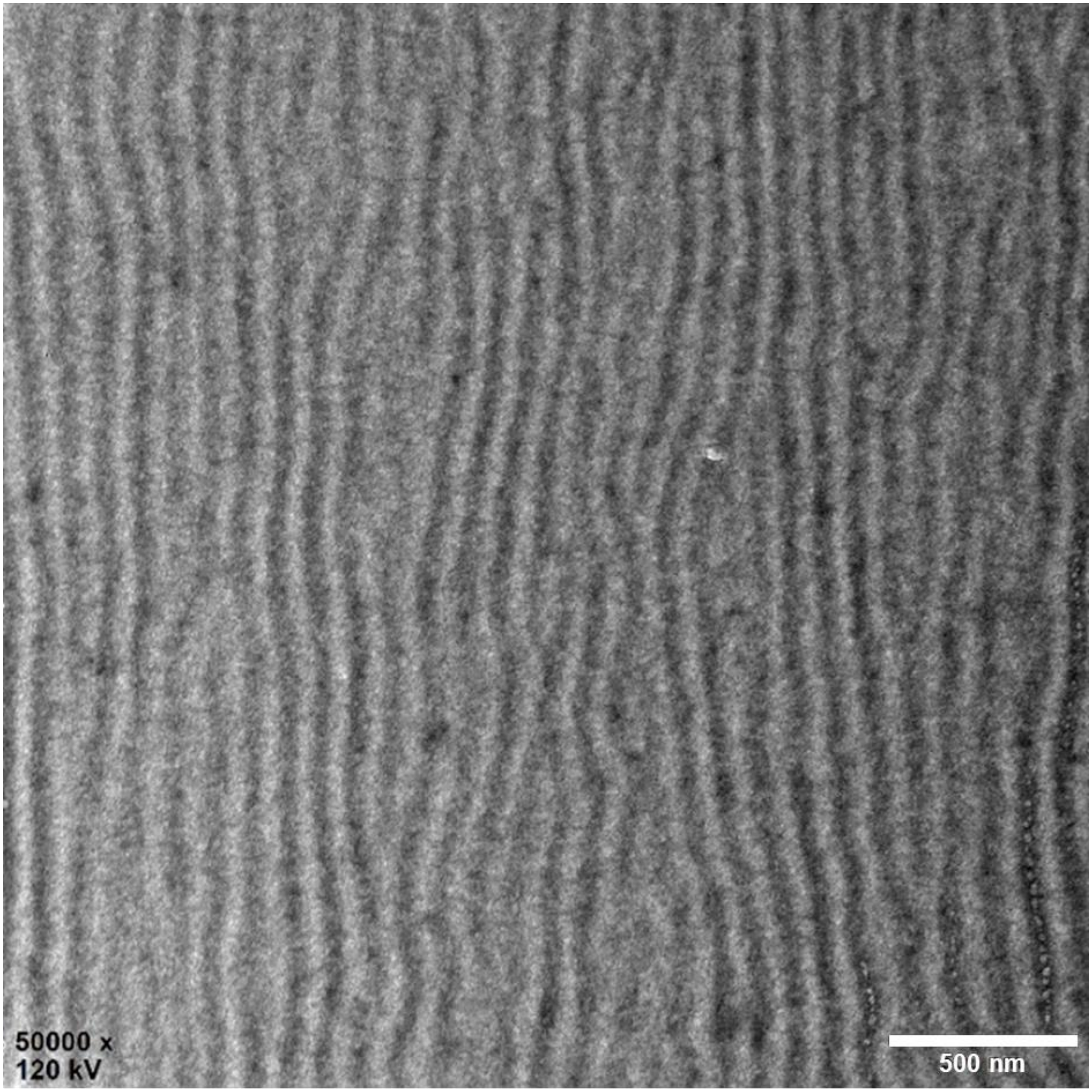

Supplementary Fig. 41. Low-magnification TEM image of the vitrified solution of P(DA_{73}-*r*-$PEGA_{48}$) (i_{DA} = 60%, N = 140) at 64 wt% concentration. The solution contained 2 wt% of $Pb(NO_3)_2$. The microtomed film was further stained with RuO_4.

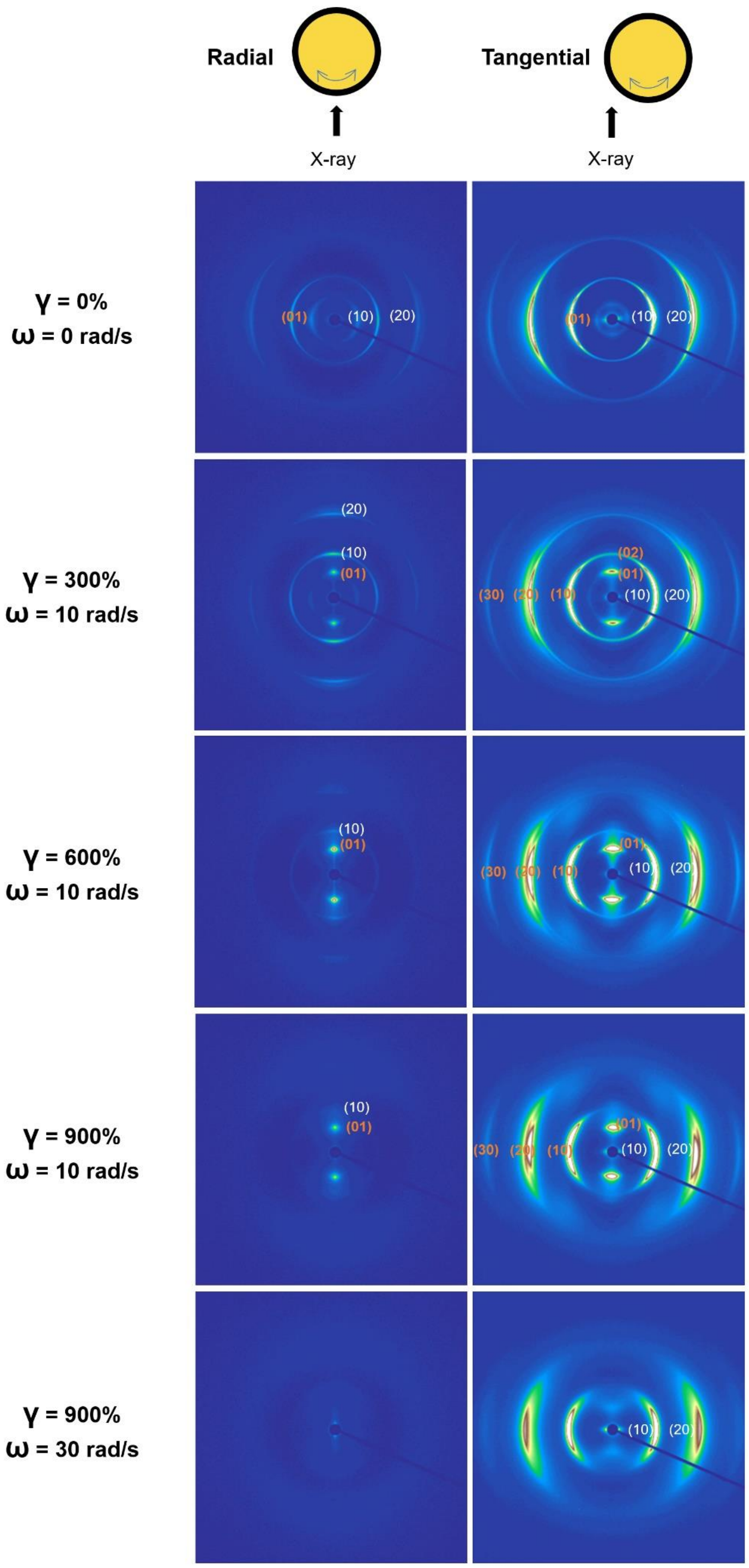

Supplementary Fig. 42. 2D SAXS pattern of P(DA$_{96}$-*r*-PEGA$_{79}$) under shear.

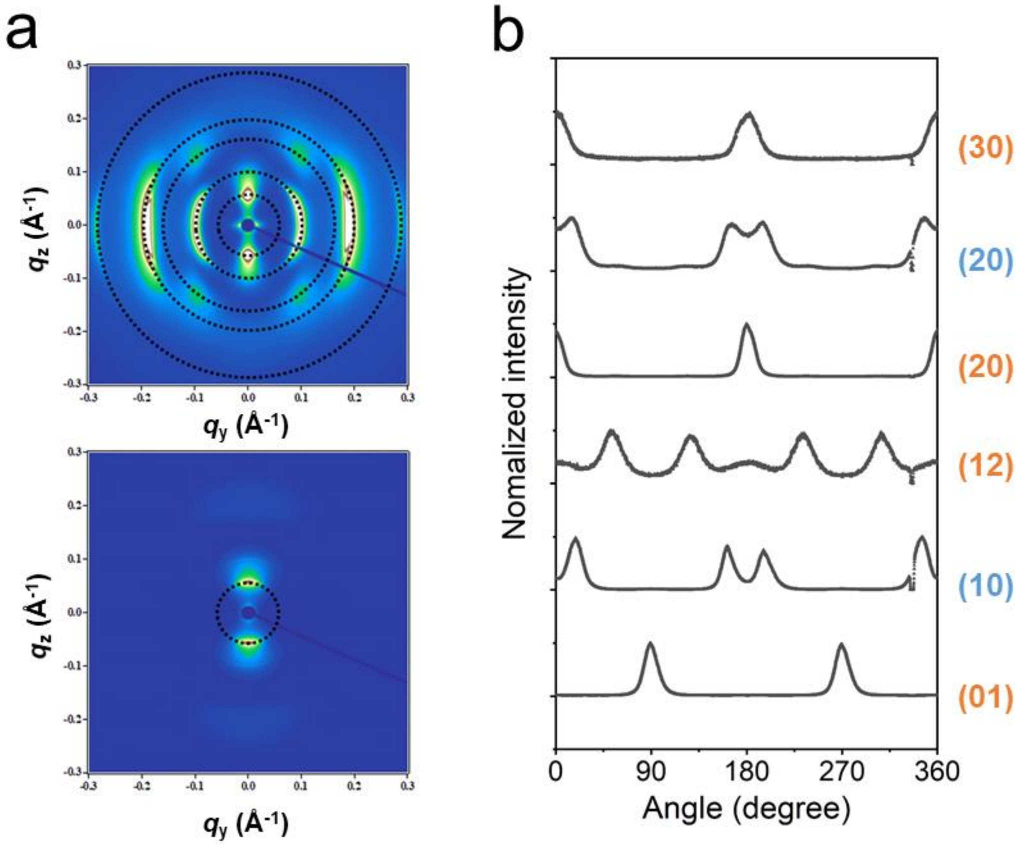

Supplementary Fig. 43. (a) Shear-aligned 2D SAXS pattern of P(DA$_{96}$-*r*-PEGA$_{79}$) shown in Fig. 3(e-f). (b) Azimuthal circular-cut of the data shown in A at different *q* positions.

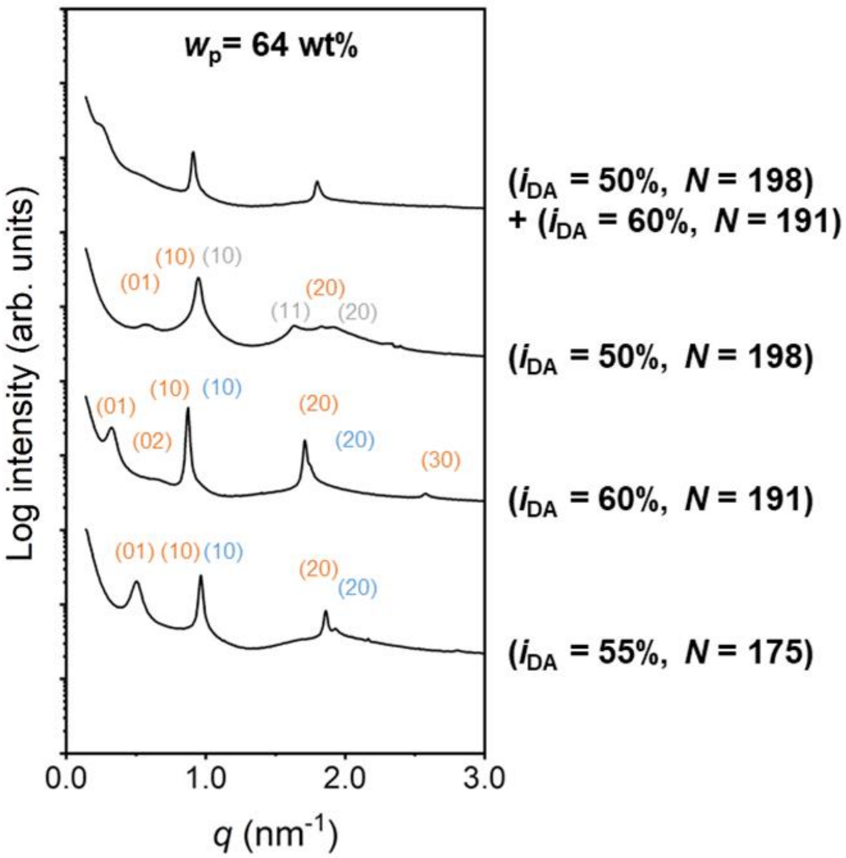

Supplementary Fig. 44. 1D SAXS data of a mixture of P(DA$_{99}$-r-PEGA$_{99}$) (i_{DA} = 50%, N = 198) and P(DA$_{115}$-r-PEGA$_{76}$) (i_{DA} = 60%, N = 191) in water. Equal amounts of the 64 wt% aqueous solutions were mixed. The SAXS patterns of P(DA$_{99}$-r-PEGA$_{99}$), P(DA$_{115}$-r-PEGA$_{76}$), and P(DA$_{96}$-r-PEGA$_{79}$) (i_{DA} = 55%, N = 175) aqueous solutions at 64 wt% concentrations are also shown as references. Miller indices for the following unit cell structure are assigned to the selected peaks in the plot: L_a (fluidic multilamellar, blue); H_I (hexagonal, grey); L_f (bilayer-folded lamellar, orange).

Supplementary Table 2. Characterization of P(DA-*grad*-PEGA)s

Sample name	Polymerization mixture composition [DA]:[PEGA]	Feed composition [DA]:[PEGA]	Conv[a]. $_{DA}$ (%)	Conv[a]. $_{PEGA}$ (%)	$M_{n,theo}$[a] (kg/mol)	i_{DA}[b] (mol %)	$M_{n,SEC}$[c] (kg/mol)	*Đ*
P(DA_{93}-*grad*-$PEGA_{92}$) (0:100)	[0]:[95]	[116]:[0]	80	97	67	50	45	1.96
P(DA_{109}-*grad*-$PEGA_{91}$) (5:95)	[6]:[90]	[106]:[5]	94	96	70	55	45	1.62
P(DA_{111}-*grad*-$PEGA_{90}$) (15:85)	[14]:[81]	[102]:[14]	96	95	70	55	43	1.86
P(DA_{108}-*grad*-$PEGA_{88}$) (25:75)	[29]:[71]	[87]:[24]	93	93	69	55	48	1.82
P(DA_{110}-*grad*-$PEGA_{90}$) (35:65)	[40]:[62]	[76]:[33]	95	95	70	55	42	1.79

[a]Determined by ^{1}H NMR spectroscopy

[b]Determined by ^{1}H NMR spectroscopy

[c]Determined by SEC analysis based on linear PMMA standards using DMF (LiBr) as an eluent

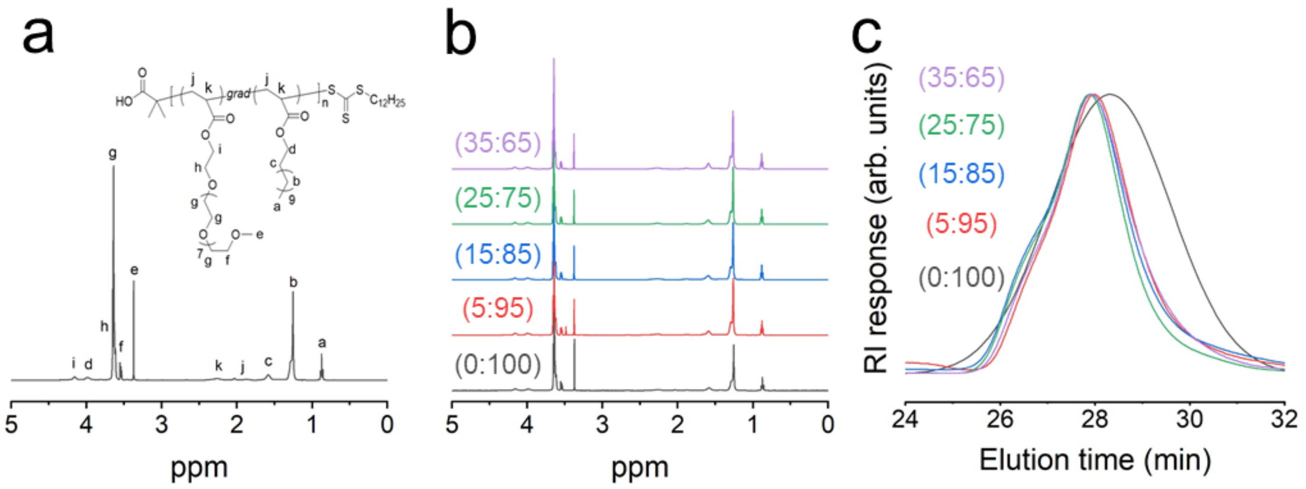

Supplementary Fig. 45. Characterization of P(DA-*grad*-PEGA)s. (a) ^{1}H NMR spectrum of P(DA_{93}-*grad*-$PEGA_{92}$)(100:0) (400 MHz, $CDCl_3$, 20 °C). (b) ^{1}H NMR spectra of P(DA-*grad*-PEGA)s. (c) SEC traces of P(DA-*grad*-PEGA)s (DMF with 0.05 M LiBr, 1 mL min^{-1}, 45 °C). The initial [DA]:[PEGA] composition in the polymerization mixture is given in the parentheses. The overall DA fraction of 55% and *N* of 210 were targeted. The i_{DA} and *N* values of the synthesized polymers were in the range of 50 – 55% and 180 – 200, respectively.

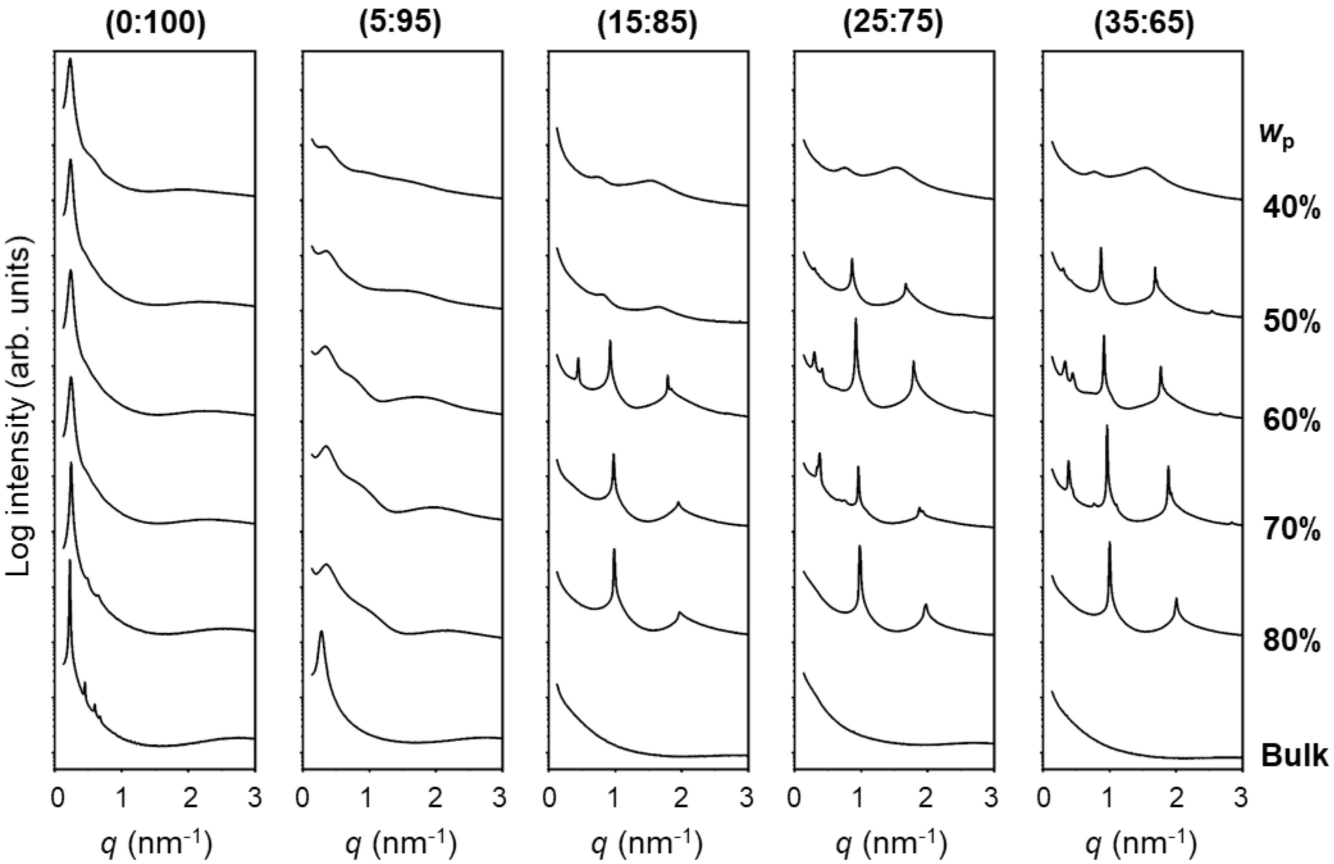

Supplementary Fig. 46. 1D SAXS data of P(DA-*grad*-PEGA) aqueous solutions at different concentrations from 40 to 100% (bulk). The initial [DA]:[PEGA] composition in the polymerization mixture is given in the parentheses. We note that the scattering peak corresponding to the bilayer-folded lamellae is split at 64 wt% solution of P(DA-*grad*-PEGA) (25:75) and (35:65). While we are not certain of its origin, the spatial composition gradient along the polymer backbone may be responsible for different folded heights.

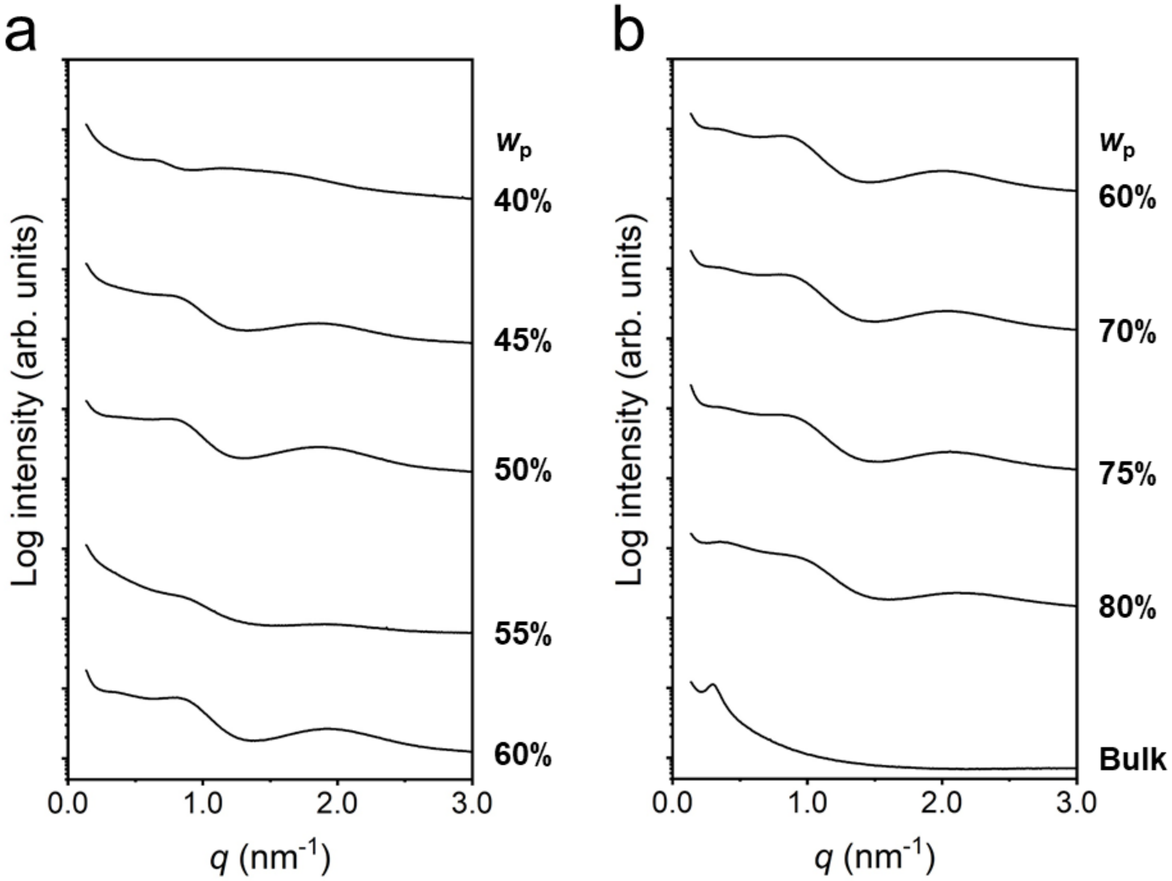

Supplementary Fig. 47. 1D SAXS data of P(DA_{144}-*co*-$PEGMA_{84}$) (i_{DA} = 65%, N = 228) aqueous solutions at different concentrations. (a) 40 to 60 wt%. (b) 65 to 100 wt% (bulk).

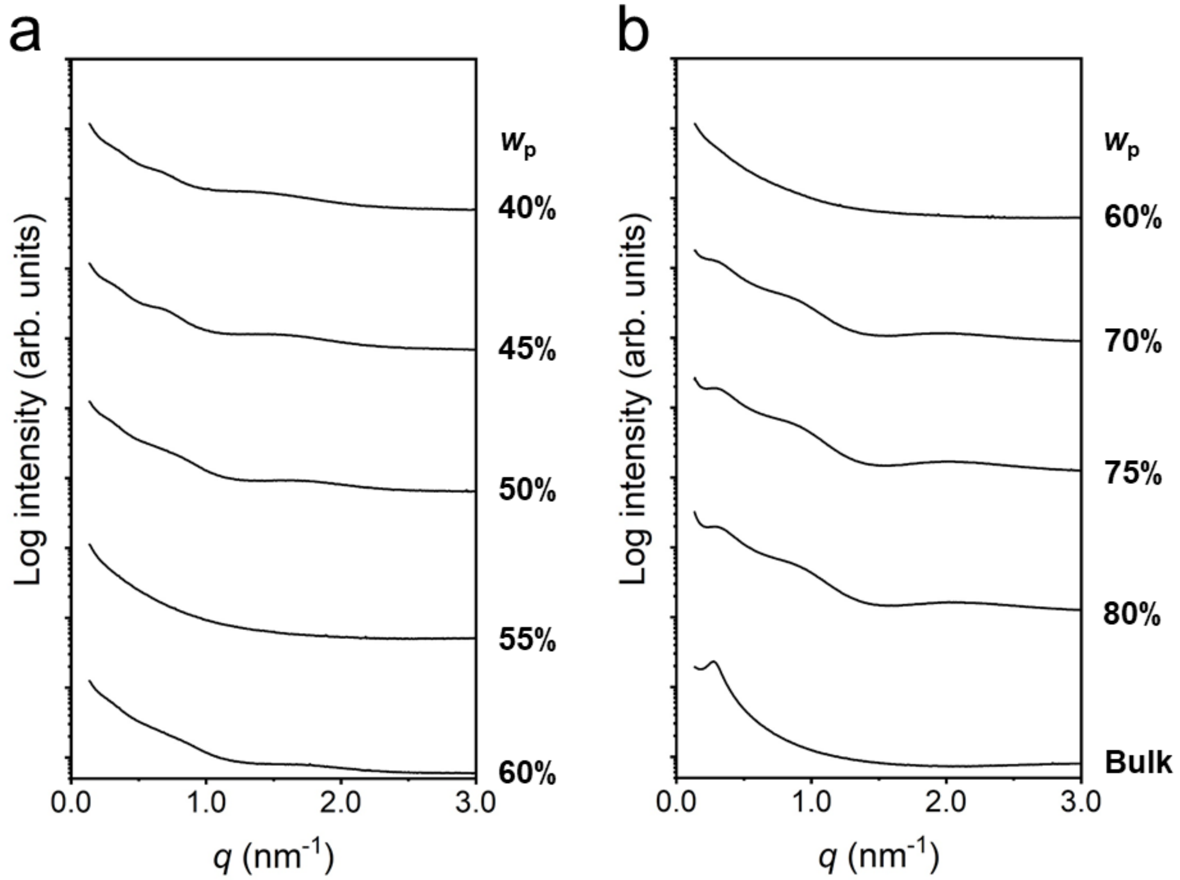

Supplementary Fig. 48. 1D SAXS data of P(DMA$_{106}$-*co*-PEGA$_{95}$) (i_{DMA} = 53%, N = 201) aqueous solutions at different concentrations. (a) 40 to 60 wt%. (b) 64 to 100 wt% (bulk).

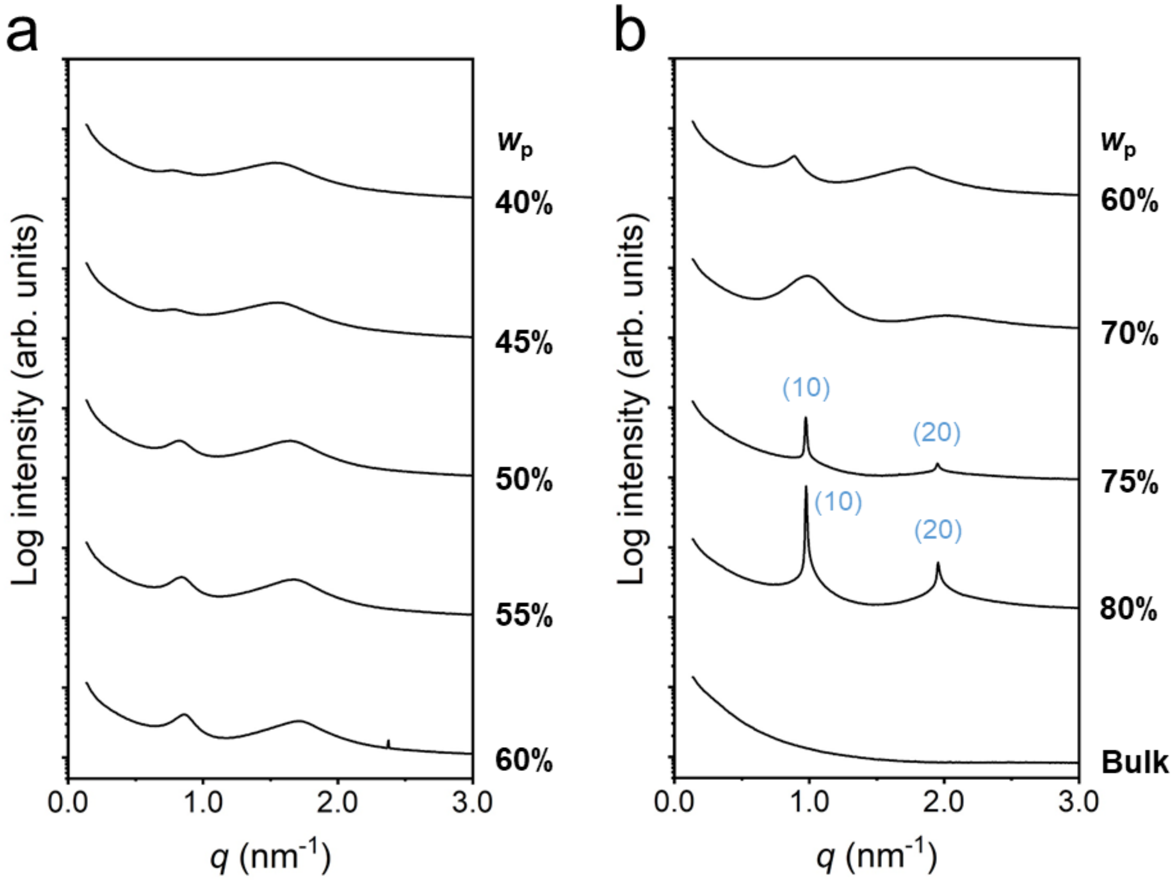

Supplementary Fig. 49. 1D SAXS data of P(DMA$_{106}$-*r*-PEGMA$_{86}$) (i_{DMA} = 55%, N = 192) aqueous solutions at different concentrations. (a) 40 to 60 wt%. (b) 65 to 100 wt% (bulk). Miller indices for the following unit cell structure are assigned to the selected peaks in the plot: L_a (fluidic multilamellar, blue).

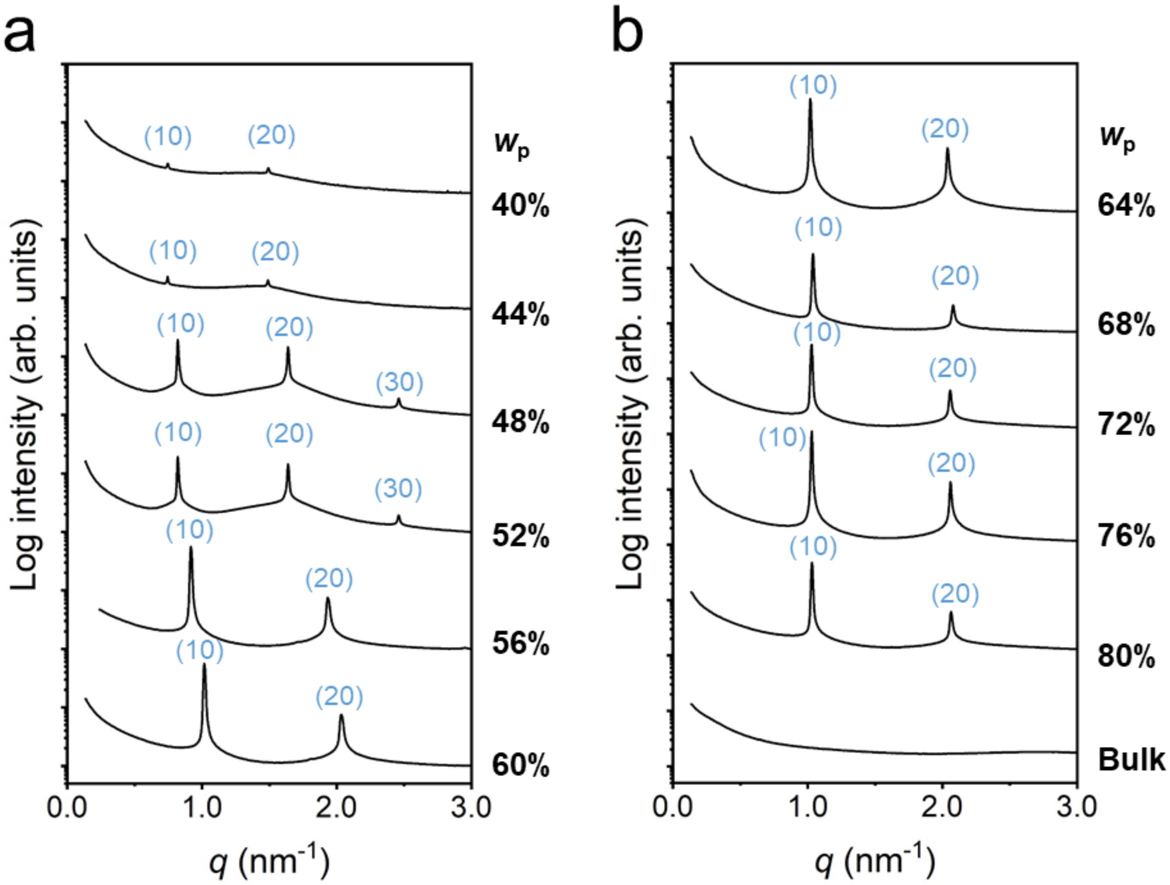

Supplementary Fig. 50. 1D SAXS data of P(DA_{21}-*r*-$PEGA_{17}$) (i_{DA} = 55%, N = 38) aqueous solutions at different concentrations. (a) 40 to 60 wt%. (b) 64 to 100 wt% (bulk). Miller indices for the following unit cell structure are assigned to the selected peaks in the plot: L_a (fluidic multilamellar, blue).

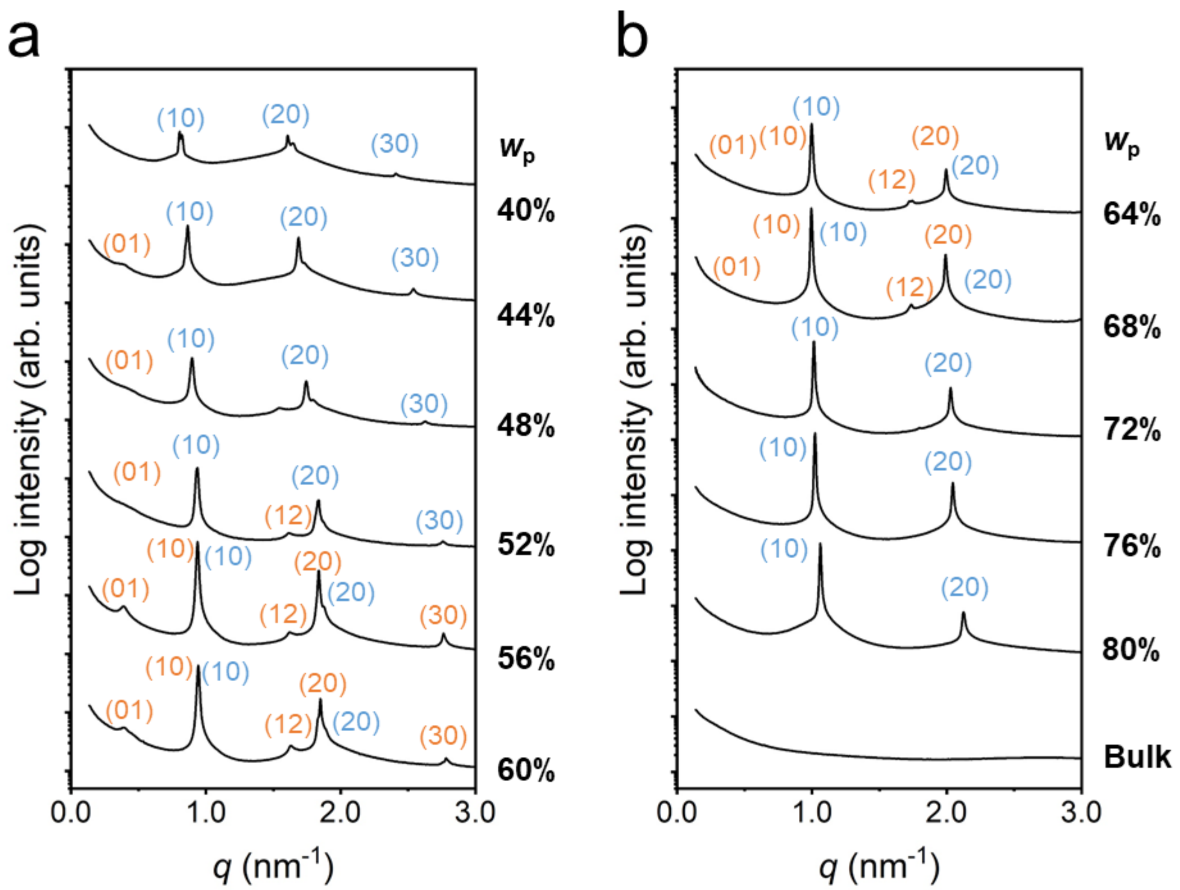

Supplementary Fig. 51. 1D SAXS data of P(DA_{21}-*r*-$PEGA_{17}$) (i_{DA} = 55%, N = 49) aqueous solutions at different concentrations. (a) 40 to 60 wt%. (b) 64 to 100 wt% (bulk). Miller indices for the following unit cell structure are assigned to the selected peaks in the plot: L_a (fluidic multilamellar, blue); L_f (bilayer-folded lamellar, orange).

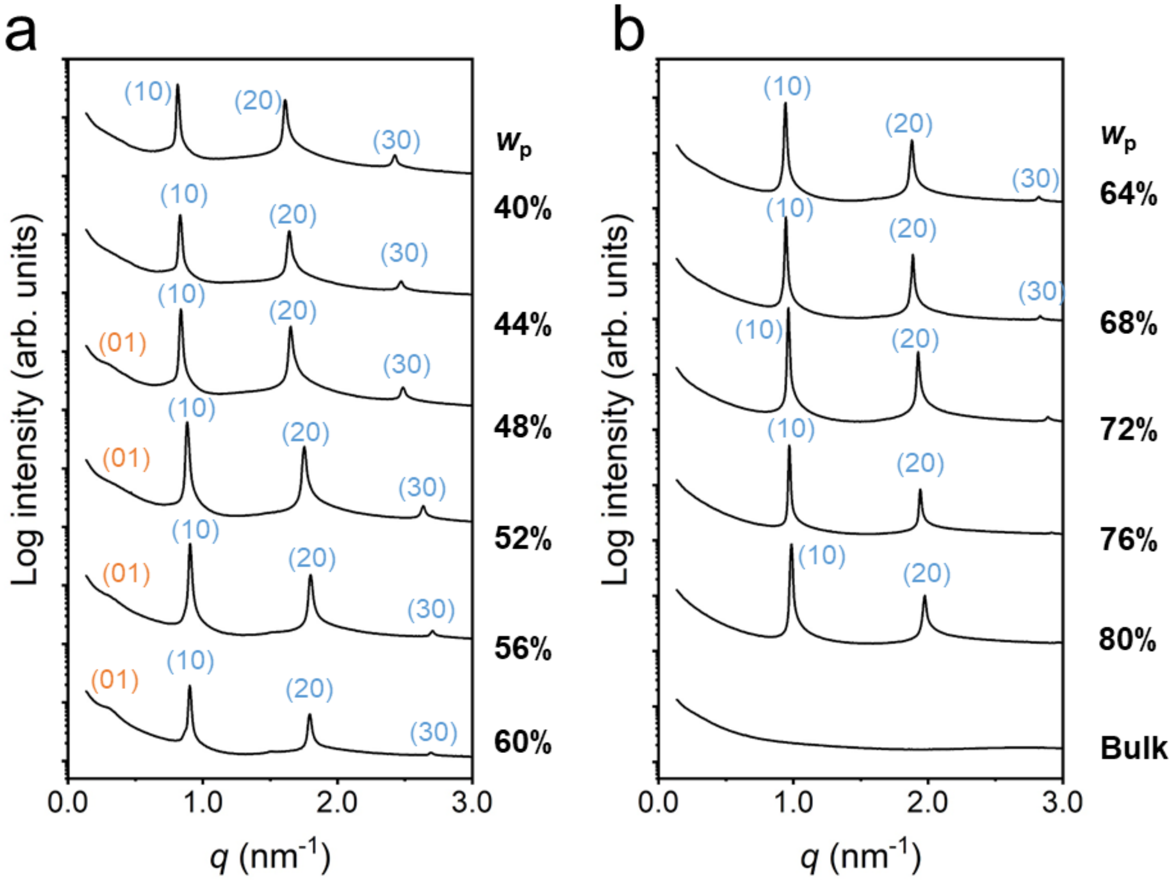

Supplementary Fig. 52. 1D SAXS data of P(DA_{29}-*r*-$PEGA_{20}$) (i_{DA} = 60%, *N* = 49) aqueous solutions at different concentrations. (a) 40 to 60 wt%. (b) 64 to 100 wt% (bulk). Miller indices for the following unit cell structure are assigned to the selected peaks in the plot: L_a (fluidic multilamellar, blue); L_f (bilayer-folded lamellar, orange).

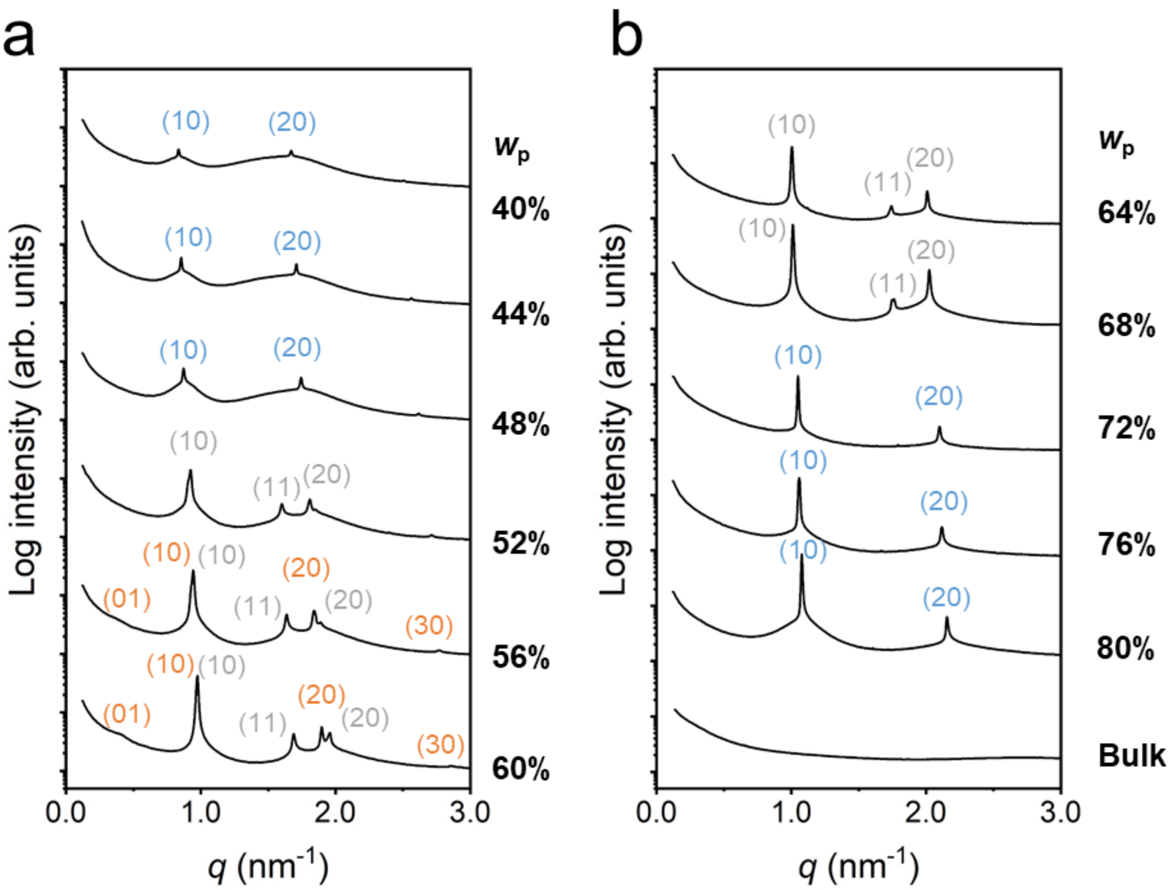

Supplementary Fig. 53. 1D SAXS data of P(DA_{25}-*r*-$PEGA_{25}$) (i_{DA} = 50%, *N* = 50) aqueous solutions at different concentrations. (a) 40 to 60 wt%. (b) 64 to 100 wt% (bulk). Miller indices for the following unit cell structure are assigned to the selected peaks in the plot: L_a (fluidic multilamellar, blue); H_I (hexagonal, grey); L_f (bilayer-folded lamellar, orange).
